# Design and Semisynthesis of Biselectrophile-Functionalized Ubiquitin Probes To Investigate Transthioesterification Reactions

**DOI:** 10.1021/acs.orglett.4c01102

**Published:** 2024-05-23

**Authors:** Avelyn Mae V. Delos Reyes, Michaelyn C. Lux, Zachary S. Hann, Cheng Ji, Tomasz Kochańczyk, Mikaela DiBello, Christopher D. Lima, Derek S. Tan

**Affiliations:** a Pharmacology Program, Weill Cornell Graduate School of Medical Sciences, Memorial Sloan Kettering Cancer Center, New York, New York 10065, United States; b Chemical Biology Program, Sloan Kettering Institute, Memorial Sloan Kettering Cancer Center, New York, New York 10065, United States; c Tri-Institutional PhD Program in Chemical BiologyMemorial Sloan Kettering Cancer Center, New York, New York 10065, United States; g Tri-Institutional Research Program, Memorial Sloan Kettering Cancer Center, New York, New York 10065, United States; d Structural Biology Program, Sloan Kettering Institute, Memorial Sloan Kettering Cancer Center, New York, New York 10065, United States; e Gerstner Sloan Kettering Summer Undergraduate Research Program, Memorial Sloan Kettering Cancer Center, New York, New York 10065, United States; f Howard Hughes Medical Institute, 1275 York Avenue, New York, New York 10065, United States

## Abstract

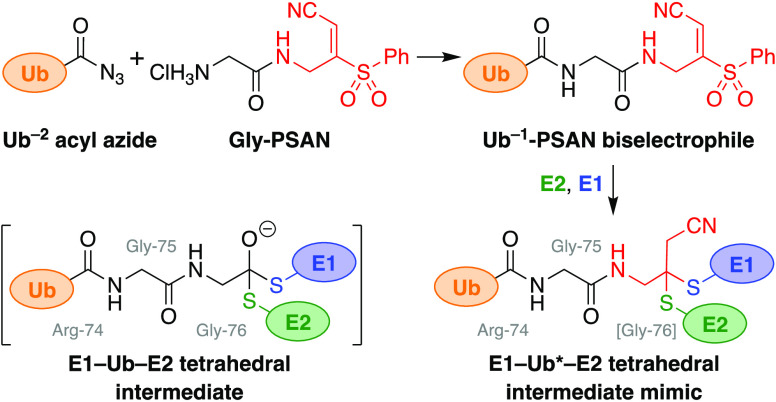

Ubiquitin (Ub) regulates a wide array of cellular processes through post-translational modification of protein substrates. Ub is conjugated at its C-terminus to target proteins via an enzymatic cascade in which covalently bound Ub thioesters are transferred from E1 activating enzymes to E2 conjugating enzymes, and then to certain E3 protein ligases. These transthioesterification reactions proceed via transient tetrahedral intermediates. A variety of chemical strategies have been used to capture E1–Ub–E2 and E2–Ub–E3 mimics, but these introduce modifications that disrupt atomic spacing at the linkage point relative to the native tetrahedral intermediate. We have developed a biselectrophilic PSAN warhead that can be installed in place of the conserved C-terminal glycine in Ub and used to form ternary protein complexes linked via cyanomethyldithioacetals that closely mimic the native tetrahedral intermediates. Investigation of the reactivity of the warhead and substituted analogues led to an effective semisynthetic route to Ub^–1^-PSAN, which was used to form a ternary E1–Ub*–E2 complex as a mimic of the transthioesterification intermediate.

Ubiquitin (Ub) is a 76-amino acid residue protein that is conjugated via its C-terminus to other proteins, primarily at lysine side chains to form isopeptide bonds. Post-translational modification with Ub and other ubiquitin-like modifier proteins can alter protein function, interactions, localization, and degradation, controlling a wide range of cellular processes.^1−3^ Ub conjugation proceeds by a reaction cascade involving three enzymes (Figure 1a).^4^ An E1 activating enzyme adenylates the C-terminus of Ub (**1**),^5^ then attacks the resulting Ub-AMP intermediate with its catalytic cysteine to form an E1∼Ub thioester (**2**).^6^ Binding of ATP·Mg^2+^ and a second Ub protein forms a doubly loaded E1 (not shown) that is most adept at recruiting an E2 conjugating enzyme, which then catalyzes transthioesterification via a tetrahedral intermediate (**3**) to form an E2∼Ub thioester (**4**). An E3 protein ligase then directs transfer of the Ub to the target protein (**4** → **7**). This can occur either by initial transthioesterification (via **5**) to an E3∼Ub thioester intermediate (**6**) in the case of HECT, RBR, and RCR-type E3s or via noncovalent scaffolding in the case of RING-type E3s.^7,8^

**Figure 1:**
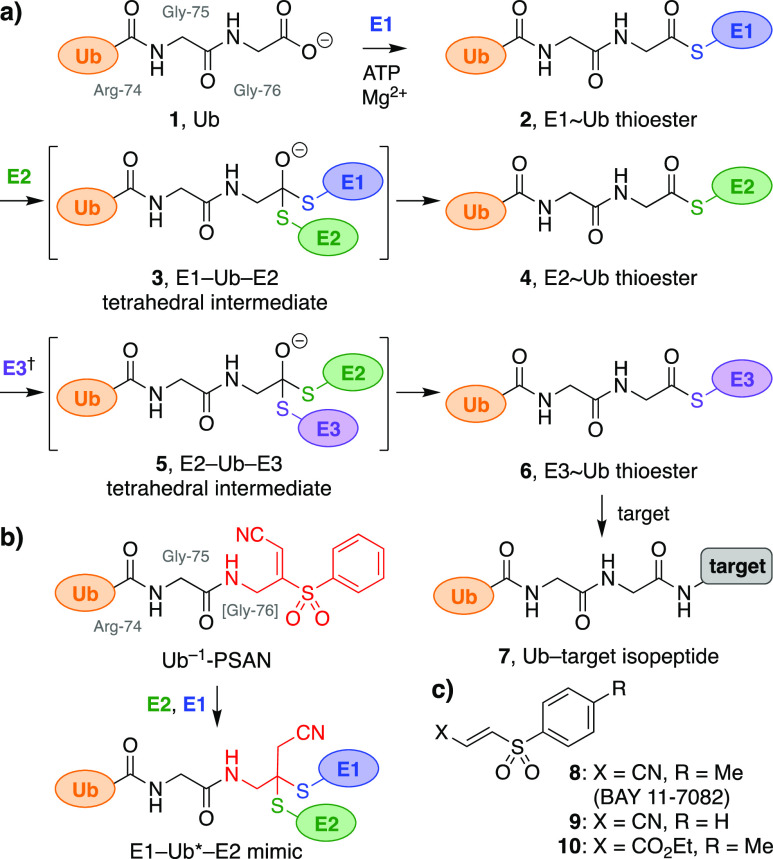
Ubiquitin conjugation cascade and probes. (a) Ub is transthioesterified from E1 activating enzymes to E2 conjugating enzymes and from E2s to thioester-forming E3 protein ligases via tetrahedral intermediates **3** and **5** (^†^RING-type E3s noncovalently catalyze Ub transfer from E2∼Ub thioesters directly to targets (**4** → **7**)). (b) Biselectrophilic Ub^–1^-PSAN probe used herein to form an E1–Ub*–E2 conjugate as mimic of tetrahedral intermediate **3** (Ub* = cyanomethyl-modified Ub). (c) Structures of related small-molecule biselectrophiles.

The biochemistry of the Ub conjugation cascade was elucidated in the 1980s,^1^ but the molecular mechanisms by which E1, E2, and E3 enzymes catalyze these reactions remain of fundamental interest.^4^ Further, the Ub pathway has become a focus in the development of E1 inhibitors and E3-recruiting molecular glues and PROTACs for therapeutic applications.^9^ We have previously used Ub-based chemical probes to inhibit E1 enzymes, uncovering dramatic enzyme remodeling during E1-catalyzed Ub adenylation and thioesterification.^10−12^ To expand upon those studies, we report herein the development of new Ub-based biselectrophilic probes that can be used to investigate downstream transthioesterification reactions to E2s and thioester-forming E3s. In contrast to previous approaches, these Ub^–1^-PSAN (3-[phenylsulfonyl]-4-aminobut-2-enenitrile)^13^ probes form ternary complexes that are designed to mimic faithfully the atomic spacing of the transthioesterification tetrahedral intermediates (Figure 1b). The PSAN warhead replaces the conserved C-terminal Gly-76 of Ub, and inclusion of the penultimate Gly-75 in the warhead fragment is critical to provide sufficient reactivity for semisynthetic installation at the C-terminus of a truncated Ub acyl azide. Kinetic studies with model substrates indicate that PSAN probes react quickly with the first cysteine nucleophile, but that the second addition of the second cysteine nucleophile requires protein mediation.

Previous studies of E1–E2 and E2–E3 transthioesterification reactions have used a variety of approaches to trap mimics of the E1–Ub–E2 or E2–Ub–E3 tetrahedral intermediates (Figure S1a).^4,14^ Lima and co-workers formed a direct disulfide linkage between the catalytic cysteines of an E1 and E2 (Figure S1b).^15^ The structure illuminated E1–E2 interactions, but the absence of the Ub substrate limited insights into the molecular mechanism of catalysis.

Recognizing the potential utility of biselectrophiles to probe these transthioesterification reactions, Virdee and co-workers investigated the reactivity of 3-tosylacrylonitrile (**8**, BAY 11-7082), and methyl 3-tosyl acrylate (**10**) (Figure 1c).^16^ BAY 11-7082 was originally discovered as a putative kinase inhibitor,^17^ but later shown to act via covalent inhibition of the human E2s Ubc13 and Ubc7, leading to inhibition of an E3 called LUBAC.^18^ Notably, the chemical reactivity of 3-(phenylsulfonyl)acrylonitrile (**9**) had also been investigated separately in studies that demonstrated regiospecific bis-addition of thiols.^19^ While the analogous secondary addition of the LUBAC E3 cysteine had not been proposed for BAY 11-7082,^18^ Virdee and co-workers demonstrated that this compound could be used to trap both an E2 cysteine and an E1 cysteine.^16^ The resulting dithioacetal was envisioned as a stable mimic of the transthioesterification tetrahedral intermediate. However, these complexes still lacked the Ub component and, indeed, the inability to form an analogous BAY 11-7082-bridged complex between an E2 and an E3 was attributed to a requirement for Ub-mediated conformational changes in the E3.

To pursue ternary complexes that included the Ub substrate, Virdee and co-workers installed the biselectrophiles at the C-terminus of Ub via Huisgen reaction.^20^ Installation of the warhead provided access to stable E2–Ub–E3 mimics,^8^ but introduced a triazole motif in place of the peptide backbone, resulting in a 2-atom extension while also omitting the Ub Arg-74 side chain, which is strictly conserved across eukaryotic evolution (Figure S1c). In another approach, Schulman and co-workers used an α-bromoenone warhead to capture ternary E2–Ub–E3 complexes.^21,22^ However, this resulted in a 3-atom insertion between the cysteine thiols compared to the native tetrahedral intermediate (Figure S1d). Recently, Liu and co-workers used a 2-((2-chloroethyl)amino)ethane-1-thiol linker to form ternary E2–Ub–E3 complexes.^23^ However, the E3 was conjugated via disulfide linkage to a non-native *N*-thioethyl side chain, resulting in separation of the two cysteine sulfurs by 6 atoms, precluding effective mimicry of the native tetrahedral intermediate (Figure S1e).

To address this problem, we sought to develop biselectrophilic warheads that would enable chemoselective capture of E1 and E2 (or E2 and E3) cysteine thiols to form more faithful mimics of the transthioesterification tetrahedral intermediates. In our own early investigations, pursued contemporaneously with the studies above, we investigated several potential biselectrophiles in model systems. Based on this work, and encouraged by the reactivity reported by Virdee, we focused on 3-phenylsulfonyl-4-aminobut-2-enenitrile (PSAN) warheads (Figure 1b). Replacement of the Ub C-terminal Gly-76 with this warhead would provide the exact atomic spacing of the native tetrahedral intermediate, replacing the oxyanion with a relatively small cyanomethyl motif (Figure S1f). However, the synthesis and reactivity of this trisubstituted olefin, compared to the disubstituted olefins studied previously, would need to be explored.

Thus, PSAN isomers *E***-14a** and *Z***-14a** were prepared from triphenylphosphonium ylide **11** by bromination, olefination,^24^ sulfonylation^25^ (*E*/*Z* isomers separable), and deprotection with *in situ* generated anhydrous HCl (Figure 2a). The resulting PSAN hydrochloride salts were water-soluble and stable to autoreaction (D_2_O, 24 h). Stereochemical configurations were tentatively assigned by analogy to literature precedent^24^ and confirmed by single-crystal X-ray analysis of *Z***-14a** (Figure S2).^26^

**Figure 2:**
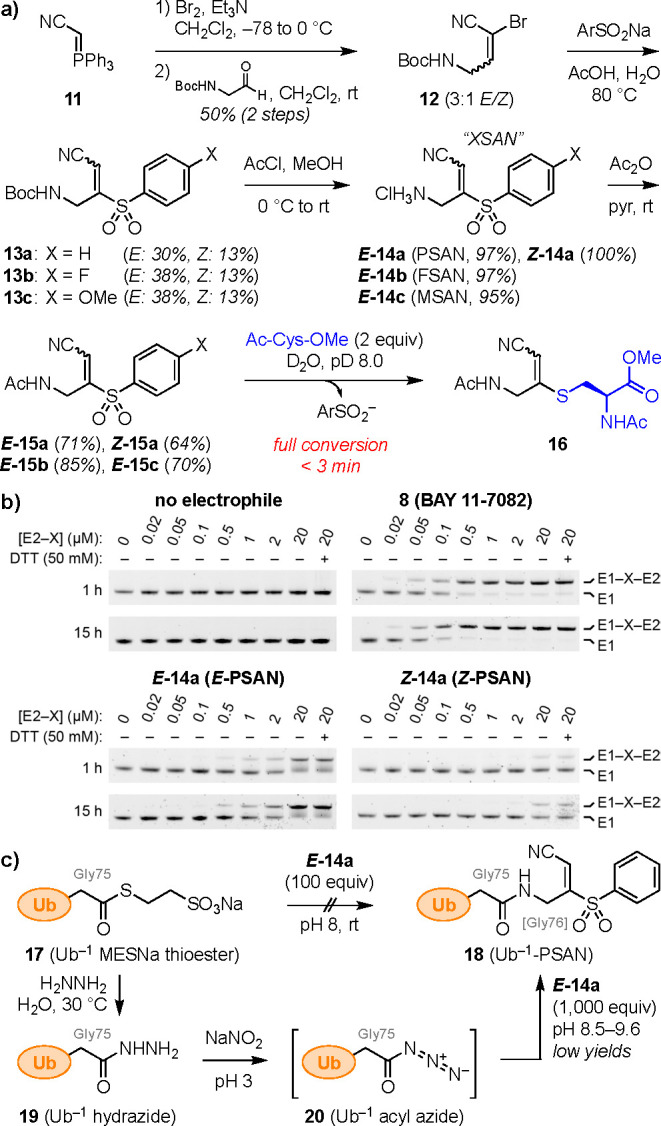
Synthesis and reactivity of XSAN warheads. (a) Synthesis of warheads **14** and model reactions with a cysteine nucleophile (blue). (b) Formation of cross-linked E1–X–E2 complexes with biselectrophiles (X).^26^ E1 = *Schizosaccharomyces pombe* Uba1 (50 nM); E2 = *S. pombe* Ubc13; X = biselectrophile; calculated MW: E1–X–E2 = 129 kDa, E1 = 112 kDa; SDS-PAGE, Sypro Ruby stain. (c) Attempted synthesis of Ub^–1^-PSAN (**18**) by aminolysis of Ub^–1^ MESNa thioester **17** (*S. pombe* Ub[1–75]; MESNa = mercaptoethanesulfonate, sodium salt) or an *in situ*-generated Ub^–1^ acyl azide **20**.

Next, we tested the reactivity of these trisubstituted olefin PSAN warheads *E***-14a** and *Z***-14a** in comparison to the disubstituted olefin BAY 11-7082 (**8**) (Figure 2b). The warheads were incubated with an E2 conjugating enzyme (*Schizosaccharomyces pombe* Ubc13) to form the initial E2–X adducts (where X is derived from the biselectrophile).^16^ This E2 was selected as it contains a single (catalytic) cysteine and represents a minimal E2 core structure.^15,16^ Addition of an E1 activating enzyme (*S. pombe* Uba1) resulted in new, higher-MW bands consistent with the E1–X–E2 conjugates, which were stable to dithiothreitol (DTT). The *E***-14a** warhead proved more reactive than *Z***-14a**, and the disubstituted olefin BAY 11-7082 (**8**) was more reactive than either trisubstituted olefin.

To investigate reactivity of the warheads further, we converted them to acetamide model systems (Ac-PSAN, *E*-**15a**, *Z*-**15a**) (Figure 2a). We also synthesized corresponding *p*-fluoro- (Ac-FSAN, *E***-15b**) and *p*-methoxy-substituted (Ac-MSAN, *E***-15c**) systems to probe the electronic effects of aromatic substituents on the initial addition–elimination reaction. In reactions with a protected cysteine, all four model systems reacted completely within 3 min (NMR) to form β-thioacrylonitriles **16**. Accordingly, we proceeded with further studies of the parent PSAN warhead *E***-14a**.

Direct aminolysis of Ub C-terminal thioesters has been used previously to install various warheads.^27^ Thus, we prepared the *S. pombe* Ub^–1^ MESNa (mercaptoethyl sulfonate, sodium salt) thioester **17**, truncated by one residue (Gly-76) at its C-terminus, via an intein-based approach (Figure 2c).^27^ However, attempted aminolysis with PSAN warhead *E***-14a** resulted in no reaction (UPLC-MS). In contrast, the thioester underwent successful transthioesterification with both protein and small-molecule thiol nucleophiles in positive control reactions (not shown).

As an alternative, we investigated aminolysis of Ub^–1^ acyl azide **20**.^12,28,29^ Thus, Ub^–1^ MESNa thioester **17** was converted to Ub^–1^ hydrazide **19**, which was then treated with NaNO_2_ to form Ub^–1^ acyl azide **20***in situ*. Aminolysis with triglycine as a model amine was achieved efficiently at elevated pH and low temperatures (Figures S3, S4). In contrast, the PSAN warhead *E***-14a** exhibited much lower reactivity, resulting in only limited amounts of the desired Ub^–1^-PSAN product **18**, insufficient for preparative applications. We posited that the phenylsulfonyl and acrylonitrile moieties of PSAN rendered the amine *E*-**14a** relatively non-nucleophilic, due to their electron-withdrawing character and/or steric hindrance.

Accordingly, we envisioned that incorporation of the penultimate Gly-75 into the warhead fragment would provide Gly-XSAN amines **22** with increased nucleophilicity and decreased steric hindrance (Figure 3). Thus, the XSAN amine hydrochloride salts **14a**–**c** were coupled with Boc-Gly-OH to afford protected intermediates **21a**–**c**, which were unstable to silica gel chromatography but could be purified by trituration. Deprotection with *in situ* generated anhydrous HCl formed the Gly-XSAN amine hydrochloride salts **22a**–**c**. Although these hydrochloride salts underwent undesired 6-*exo*-*trig* cyclization in aqueous solution (*t*_1/2_ ≈ 20 min), they were completely stable at −20 °C (amorphous solid, >3 years).

**Figure 3:**
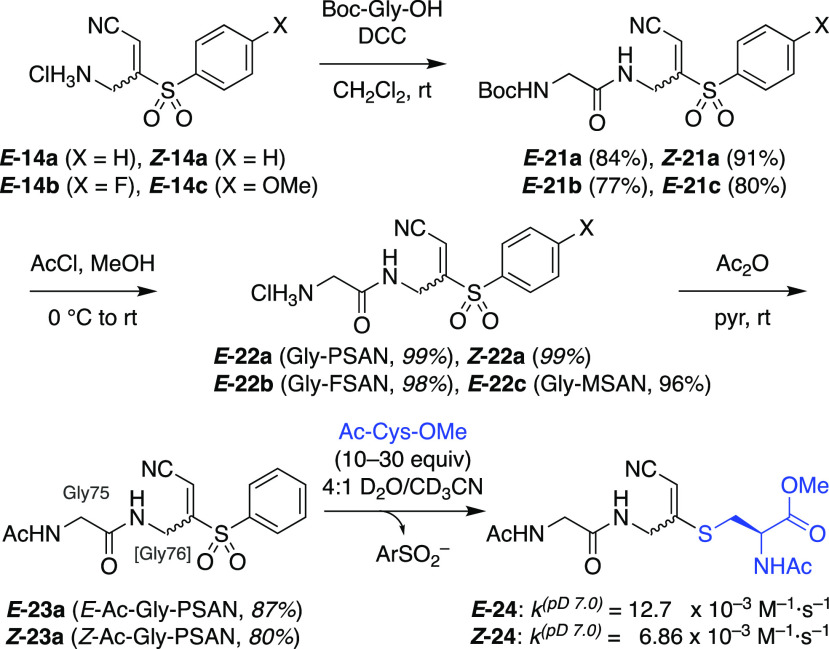
Synthesis and reactivity of Gly-XSAN warheads. Second-order rate constant for Ac-Cys-OMe addition to *E***-23a** and *Z***-23a** obtained via pseudo-first-order kinetic analysis with *k*_obs_ plotted against Ac-Cys-OMe concentration.^26^

To investigate the reactivity of these warheads in detail, we converted both isomers of Gly-PSAN (*E***-22a**, *Z***-22a**) to acetamide model systems (Ac-Gly-PSAN, *E***-23a**, *Z***-23a**) (Figure 3). Both reacted rapidly with Ac-Cys-OMe to form β-thioacrylonitriles **24** with complete conversion within 3 min at pD 8 (NMR). To enable differentiation of reaction rates of the two isomers, we lowered the pD to 7 to decrease the reactivity of the thiol, allowing pseudo-first-order kinetics to be analyzed by NMR (Figure S5).^26^ The *E* isomer (*E***-23a**) reacted nearly two times faster than the *Z* congener (*Z***-23a**) (Figure 3), consistent with the reactivity trends observed for the parent warheads **14a** above (Figure 2b). Notably, despite the use of excess thiol nucleophile (10–30 equiv), no conversion to the bis-adduct dithioacetals was observed, even upon raising the pD to 10 (Figure S6a). Moreover, under these conditions, treatment with ethane-1,2-dithiol resulted only in single addition to *E*-Ac-Gly-PSAN (*E***-23a**) as well as to BAY 11–7082 (**8**) (Figure S6b,c). In contrast, in organic solvent (4 equiv Et_3_N, CHCl_3_, rt, 2 h), BAY 11-7082 (**8**) underwent double addition to the corresponding dithiolane as previously reported,^19^ while *E*-Ac-Gly-PSAN (*E***-23a**) again underwent only single addition.^26^ This indicates marked differences in reactivity between the disubstituted olefin BAY 11-7082 (**8**) and the trisubstituted olefin *E*-Ac-Gly-PSAN (*E***-23a**), as well as solvent dependence of this reactivity.

We next turned to installation of the Gly-PSAN fragment *E*-**22a** at the C-terminus of Ub (Figure 4a). To maintain proper positioning of the warhead, the Ub construct was truncated by an additional residue (removing Gly-75 and Gly-76).^26^ The Ub^–2^ MESNa thioester **25** was converted to Ub^–2^ hydrazide **26**, followed by *in situ* formation of Ub^–2^ acyl azide **27**. Aminolysis with Gly-PSAN (*E***-22a**) afforded the desired Ub^–1^-PSAN probe **18a**, with Gly-75 derived from the warhead fragment and PSAN replacing Gly-76.^26^ The reaction proceeded effectively with 100 equiv Gly-PSAN, but use of 10 equiv Gly-PSAN led to increased hydrolysis (Figure S7). Increasing the pH from 8 to 9 to increase amine reactivity also increased hydrolysis (Figure S8). The Gly-FSAN (*E***-22b**) and Gly-MSAN (*E***-22c**) fragments were analogously coupled to the Ub^–2^ acyl azide **27** to afford Ub^–1^-FSAN (**18b**) and Ub^–1^-MSAN (**18c**) (Figures S9, S10). The Ub^–1^-XSAN probes were then purified by size-exclusion chromatography^26^ for further study.^12^ All three probes were stable in aqueous solution at rt over 28 h (Figure S11).

**Figure 4:**
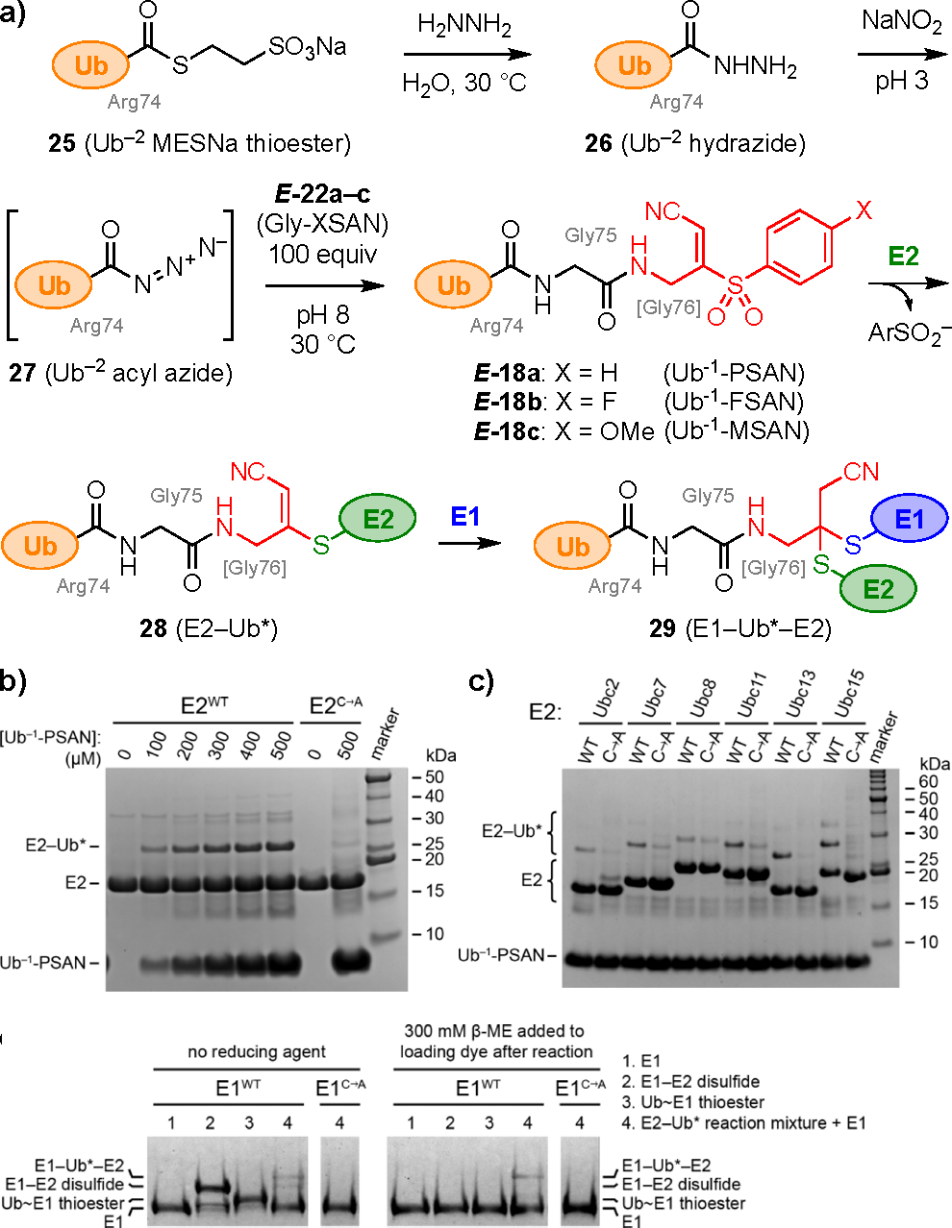
Synthesis and reactivity of Ub^–1^-XSAN probes.^26^ (a) Synthesis of Ub^–1^-XSAN probes **18a**–**c**^12^ and reaction with an E2 to form E2–Ub* intermediate **28**, then with an E1 to form E1–Ub*–E2 ternary complex **29** (Ub* = cyanomethylidiene- or cyanomethyl-modified Ub). (b) Ub^–1^-PSAN **18a** (0–500 μM) reacts with wild-type E2 (*S. pombe* Ubc13, 200 μΜ), but not an C86A mutant that lacks the catalytic cysteine, to form E2–Ub* intermediate **28**. (c) Reaction of Ub^–1^-PSAN **18a** (400 μM) with other E2s (200 μM) to form E2–Ub* intermediates **28**. (d) Reaction of the unpurified E2–Ub* intermediate **28** (*S. pombe* Ubc13, 60 μM total input to Ub^–1^-PSAN conjugation) with an E1 (*S. pombe* Uba1, 10 μM) forms E1–Ub*–E2 complex **29**. The tetrahedral intermediate mimic **29** (lanes 4) is not cleaved by β-mercaptoethanol (right), in contrast to E1–E2 disulfide and E1∼Ub thioester controls (lanes 2,3), and is not formed with an E1 C593A mutant lacking the catalytic cysteine. SDS-PAGE, Coomassie stain for all gels.

Next, we investigated the reactivity of the Ub^–1^-XSAN probes with E2 conjugating enzymes (Figure 4a). The probes were coupled with E2s first because many of these enzymes have only a single cysteine (the catalytic cysteine), in comparison to E1 activating enzymes (*S. pombe* Uba1 has 19 cysteines).^15^ Moreover, we envisioned that the resulting E2–Ub* complex **28** (Ub* represents cyanomethylidene-modified Ub) could then be coupled with either an E1 or an E3 protein ligase, providing access to both the E1–Ub*–E2 and E2–Ub*–E3 ternary complexes.^16^ Thus, Ub^–1^-PSAN (**18a**) was incubated with an E2 (*S. pombe* Ubc13), resulting in formation of new bands corresponding to the expected molecular weight of the E2–Ub* adduct (**28**) (Figure 4b). In contrast, the reaction did not proceed with an E2 (C86A) mutant, consistent with specific conjugation at the catalytic cysteine. Notably, most E2s do not have intrinsic affinity for Ub at the active site, so this reaction is driven solely by chemical reactivity. Evaluation of pH dependence identified pH 8 as optimal (Figure S12). In reactions of the Ub^–1^-FSAN (**18b**) and Ub^–1^-MSAN (**18c**) probes with two different E2s (*S. pombe* Ubc13 and Ubc15), all warheads reacted with comparable efficiency (Figure S13). Thus, further studies were pursued with the parent Ub^–1^-PSAN probe (**18a**).

We tested coupling of Ub^–1^-PSAN (**18a**) to a variety of E2 conjugating enzymes (*S. pombe* Ubc2, Ubc7, Ubc8, Ubc11, Ubc13, Ubc15) (Figure 4c). All of the E2s conjugated successfully with the probe, which was decreased or eliminated for the corresponding C → A catalytic cysteine mutants. Each of these E2s contains only a single reactive cysteine residue, thus, the observation of residual higher-MW bands with some of the C → A mutants suggests that the Ub^–1^-PSAN probe may react partially at other sites, indicating that further optimization may be required for applications to these E2s. Based on these results, we elected to proceed with Ubc13 for further cross-linking to E1.

Finally, we investigated additions of an E1 activating enzyme (*S. pombe* Uba1) to the E2–Ub* (Ubc13) intermediate (**28**). We observed formation of a new band having apparent MW consistent with that of the desired E1–Ub*–E2 complex (Figure S14), which could not be cleaved by β-mercaptoethanol, in contrast to E1–E2 disulfide and native E1∼Ub thioester controls (Figure 4d). Further, an E1 (C593A) mutant was unreactive, consistent with dithioacetal formation at the desired catalytic cysteine residue, and not any of the other 18 cysteines in the E1. Taken together, these results support the identity and structure of the new conjugate and demonstrate the feasibility of using PSAN-based probes to form ternary complexes that mimic the E1–Ub–E2 intermediate.

In summary, we have developed a biselectrophilic PSAN warhead that can be installed at the C-terminus of Ub and reacted sequentially with an E2 conjugating enzyme and an E1 activating enzyme to form a stable ternary complex that mimics the E1–Ub–E2 transthioesterification intermediate. This approach should also enable the preparation of related E2–Ub–E3 mimics. The resulting dithioacetal adduct precisely matches the atomic spacing of the native tetrahedral intermediate. The oxyanion is replaced by a cyanomethyl group, and it remains to be seen if this difference may impact interactions with the proteins that stabilize the tetrahedral intermediate. Chemical insights into the reactivity of the amino group in the warhead were essential to enabling effective conjugation with a Ub^–2^ acyl azide. Interestingly, in studies with small-molecule model systems, the second addition reaction did not proceed, even under forcing conditions, in contrast to the observed second addition of the E1 activating enzyme (Uba1) in the protein system. This suggests that the second addition is mediated by the protein, either through protein–protein interactions that increase the local concentration of the cysteine nucleophile, or through active-site catalysis. Consistent with this effect, the second reaction occurs selectively at the catalytic cysteine of the E1, and not at any of the 18 noncatalytic cysteines in Uba1. Analogous selectivity with BAY 11-7082 has been reported.^16^ Recently, we have developed further optimized protocols for preparation of this Ub^–1^-PSAN probe and its use to form E1–Ub*–E2 as well as E2–Ub*–E3 ternary complexes, enabling detailed structural and biochemical analyses that have provided new insights into the molecular mechanisms of these transthioesterification reactions and are reported separately.^30^

## Data Availability

The data underlying this study are available in the published article and its Supporting Information.

## Supporting Information Available

Supplementary figures, materials and methods, experimental protocols, analytical data, X-ray crystallographic analysis of *Z***-14a**, NMR spectra, MS spectra, and gel source images (PDF)
