# Supplementary material for: Design and Semisynthesis of Biselectrophile-Functionalized Ubiquitin Probes To Investigate Transthioesterification Reactions
Source: Org Lett. 2024 May 23;26(22):4594–9. doi: 10.1021/acs.orglett.4c01102 (PMC11165569; doi:10.1021/acs.orglett.4c01102)
Supplement: Supplementary file 1 — ol4c01102_si_001.pdf [file ol4c01102_si_001.pdf]

Design and Semisynthesis of Biselectrophile-functionalized Ubiquitin Probes to Investigate Transthioesterification Reactions

Avelyn Mae V. Delos Reyes,[†,a,b] Michaelyn C. Lux,[†,b,c] Zachary S. Hann,[†,c,d] Cheng Ji,[b] Tomasz Kochańczyk,[d] Mikaela DiBello,[b,e] Christopher D. Lima,*[,c,d,f] Derek S. Tan*[,a,b,c]

[a]Pharmacology Program, Weill Cornell Graduate School of Medical Sciences,
[b]Chemical Biology Program, Sloan Kettering Institute,
[c]Tri-Institutional PhD Program in Chemical Biology,
[d]Structural Biology Program, Sloan Kettering Institute,
[e]Gerstner Sloan Kettering Summer Undergraduate Research Program
Memorial Sloan Kettering Cancer Center
1275 York Avenue, New York, New York 10065

and

[f]Howard Hughes Medical Institute, 1275 York Avenue, New York, New York 10065 USA

[†]These authors contributed equally to this work

Supporting Information

A. Supplementary Figures S1–S14 S2
B. Materials and methods S13
C. Synthesis of XSAN (**14**) and Ac-XSAN (**15**) S15
D. Synthesis of Gly-XSAN (**22**) and Ac-Gly-XSAN (**23**) S21
E. Reactivity of Ac-XSAN probes (**15**) S25
F. Kinetic studies of thiol additions to Ac-Gly-PSAN (**23**) S26
G. X-ray crystallographic analysis of ***Z*-14a** S27
H. Cloning, expression, and protein purification S29
I. Formation of E1–X–E2 complexes from BAY 11-7082 (**8**) or PSAN (**14**) S30
J. Semisynthesis and conjugation of Ub^{-1}-XSAN probes S31
K. Supporting information references S33
L. ^{1}H-NMR and ^{13}C-NMR spectra S34
M. Mass spectra (full scans for Supplementary Figures S7–S11) S60
N. SDS-PAGE gels (source images for Figures 2b, 4b–d, S12, S13) S66

A. SUPPLEMENTARY FIGURES S1-S14

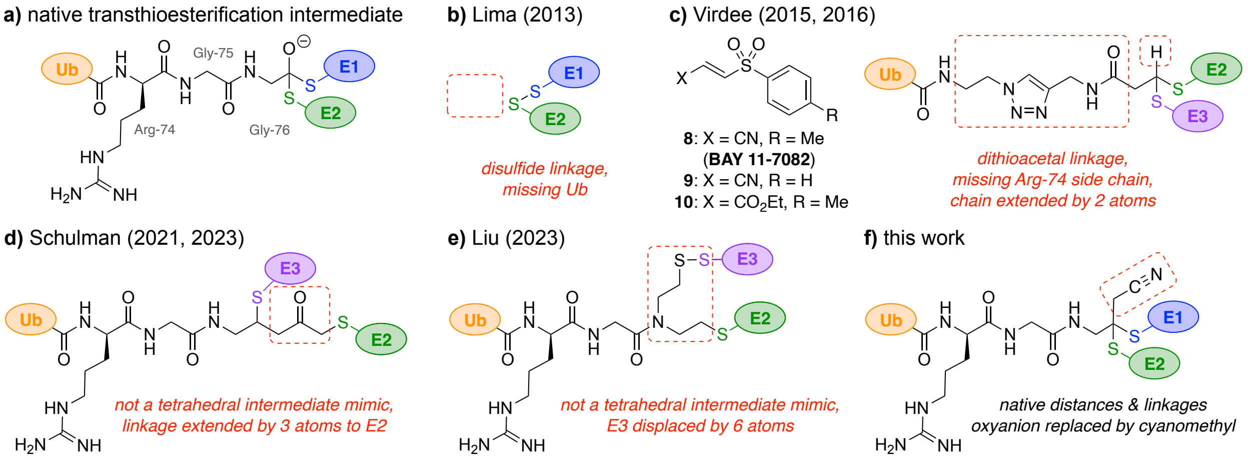

Figure S1. Mimics of tetrahedral intermediates. (a) Native tetrahedral intermediate in transthioesterification reactions (shown for E1–Ub–E2). **(b)** Binary E1–E2 complex with disulfide linkage between catalytic cysteines, but lacking Ub.[1] **(c)** Ternary E2–Ub–E3 complex based on biselectrophiles (**8**, **10**),[2] with dithioacetal mimic, but lacking Arg-74 side chain and extending main chain by 2 atoms.[3] **(d)** Ternary E2–Ub–E3 complex with native distance to E3, but 3-atom insertion to E2.[4,5] **(e)** Ternary E2–Ub–E3 complex with native distance to E2, but 6-atom displacement of E3 onto non-native *N*-thioethyl side chain.[6] **(f)** Ternary E1–Ub–E2 complex with native distance to dithioacetal mimic, and conservative replacement of oxyanion with cyanomethyl substituent, synthesized herein.

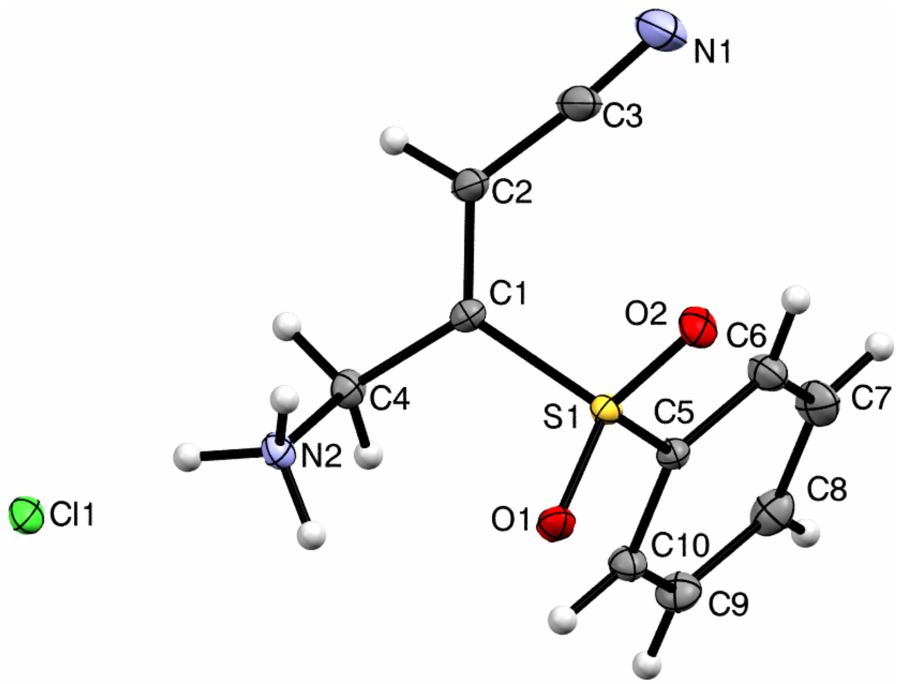

Figure S2. X-ray crystal structure of (*Z*)-PSAN warhead (*Z*-14a). Ellipsoid contour probability level = 50% (CCDC 2336089). See SECTION G for complete details.

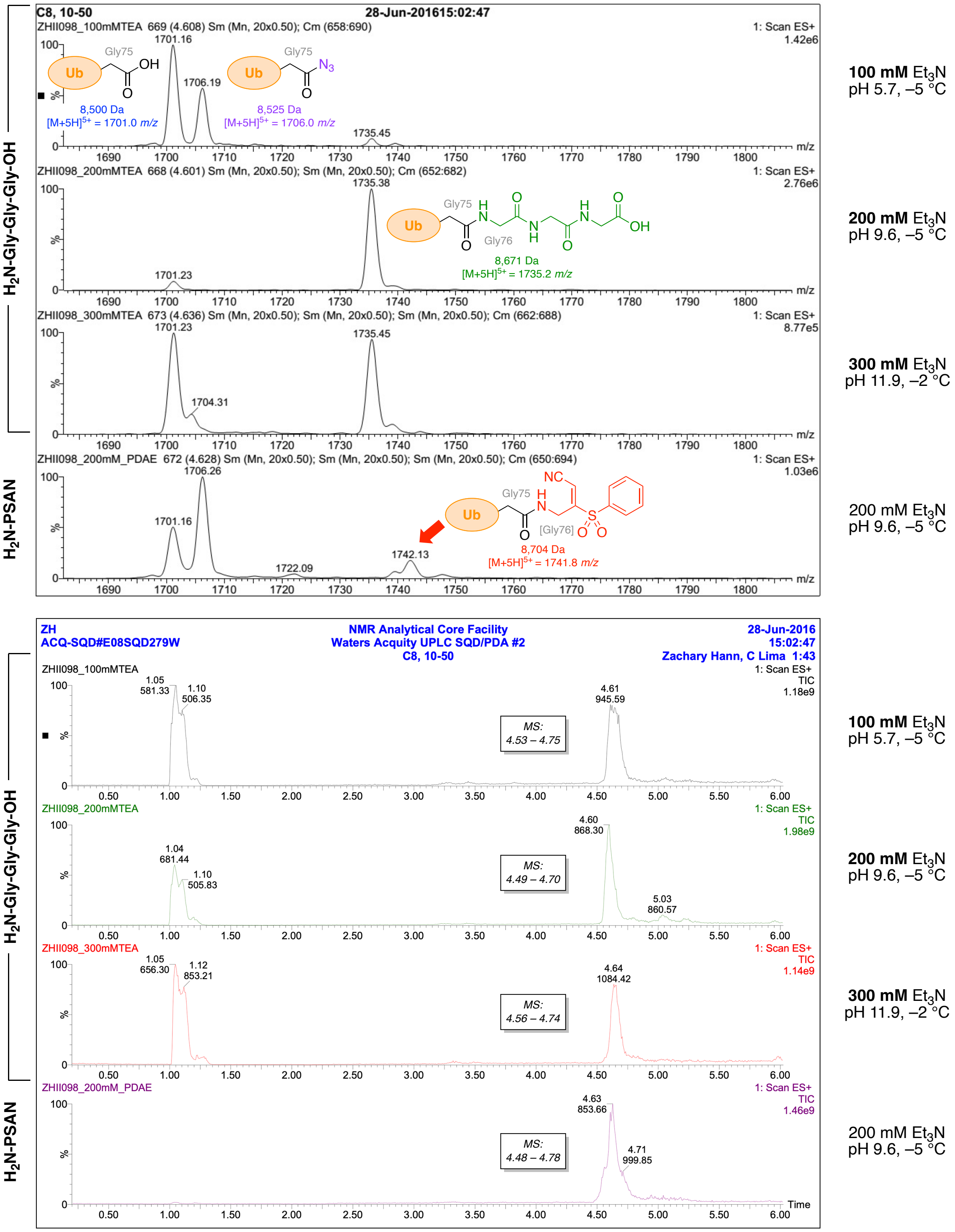

Figure S3. Aminolysis of Ub⁻¹ acyl azide (20) with triglycine at various Et_3N concentrations, or with PSAN amine (*E*-14a). Mass spectra (top) integrated over the HPLC range indicated (bottom). *Conditions*: 100 μM Ub⁻¹ hydrazide (**19**), 250 mM $NaNO_2$, 50 mM citrate, pH 3, –5 °C, 2 min; then 100 mM triglycine or PSAN amine·HCl (***E*-14a**), Et_3N as indicated (resulting pH as indicated), 2 min.

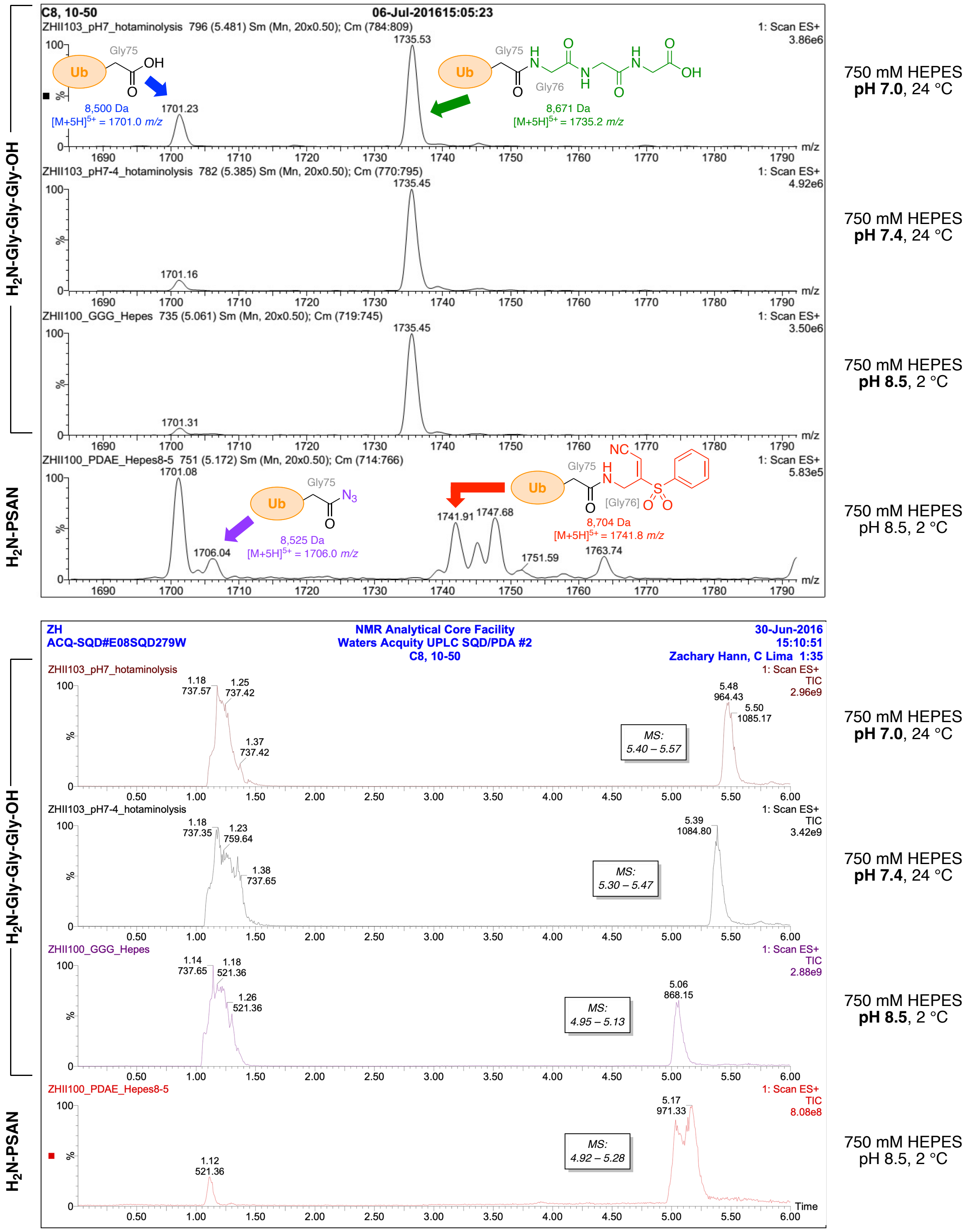

Figure S4. Aminolysis of Ub^{-1} acyl azide (20) with triglycine at various pH, or with PSAN amine (*E*-14a). Mass spectra (top) integrated over the HPLC range indicated (bottom). *Conditions*: 100 μM Ub^{-1} hydrazide (**19**), 250 mM $NaNO_2$, 50 mM citrate, pH 3, –5 °C, 2 min; then 100 mM triglycine or PSAN amine·HCl (***E*-14a**), 750 mM HEPES, pH as indicated, 2 min.

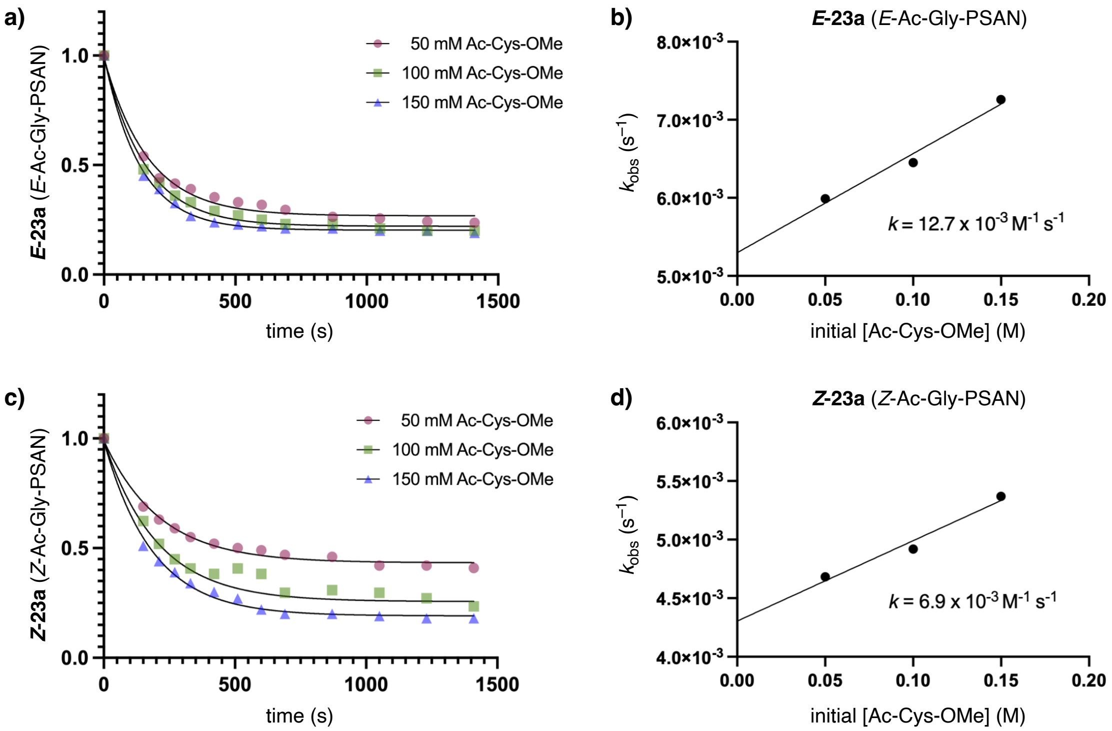

Figure S5. Pseudo first-order kinetic analysis. Rate of Ac-Cys-OMe addition to ***E*-23a** (**a**,**b**) and ***Z*-23a** (**c**,**d**) obtained via pseudo first-order kinetics with k_{obs} plotted against Ac-Cys-OMe concentration. *Conditions:* 5 mM **23a**, 4:1 PBS (D_2O)/CD_3CN (as described in Section F), pD 7.0 (conversion: *pD = pH meter reading + 0.40*).

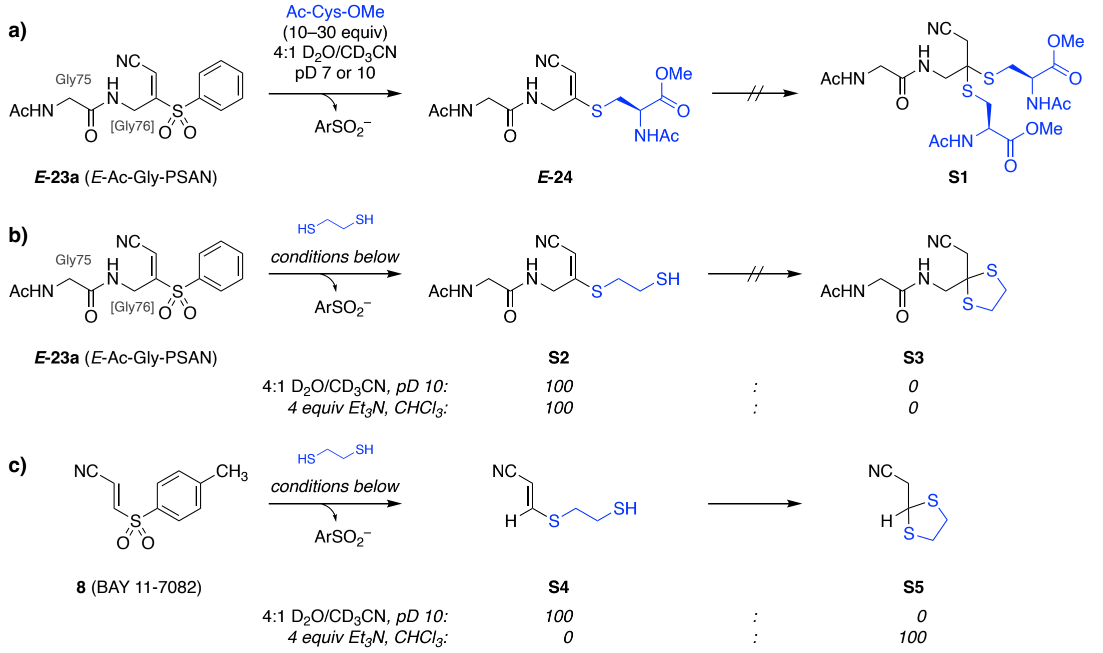

Figure S6. Reactions of biselectrophiles with small-molecule thiol nucleophiles. *Conditions (aqueous):* 5 mM biselectrophile, 50–150 mM Ac-Cys-OMe, 4:1 PBS (D_2O)/CD_3CN (as described in Section F.), pD as indicated (conversion: *pD = pH meter reading + 0.40*).

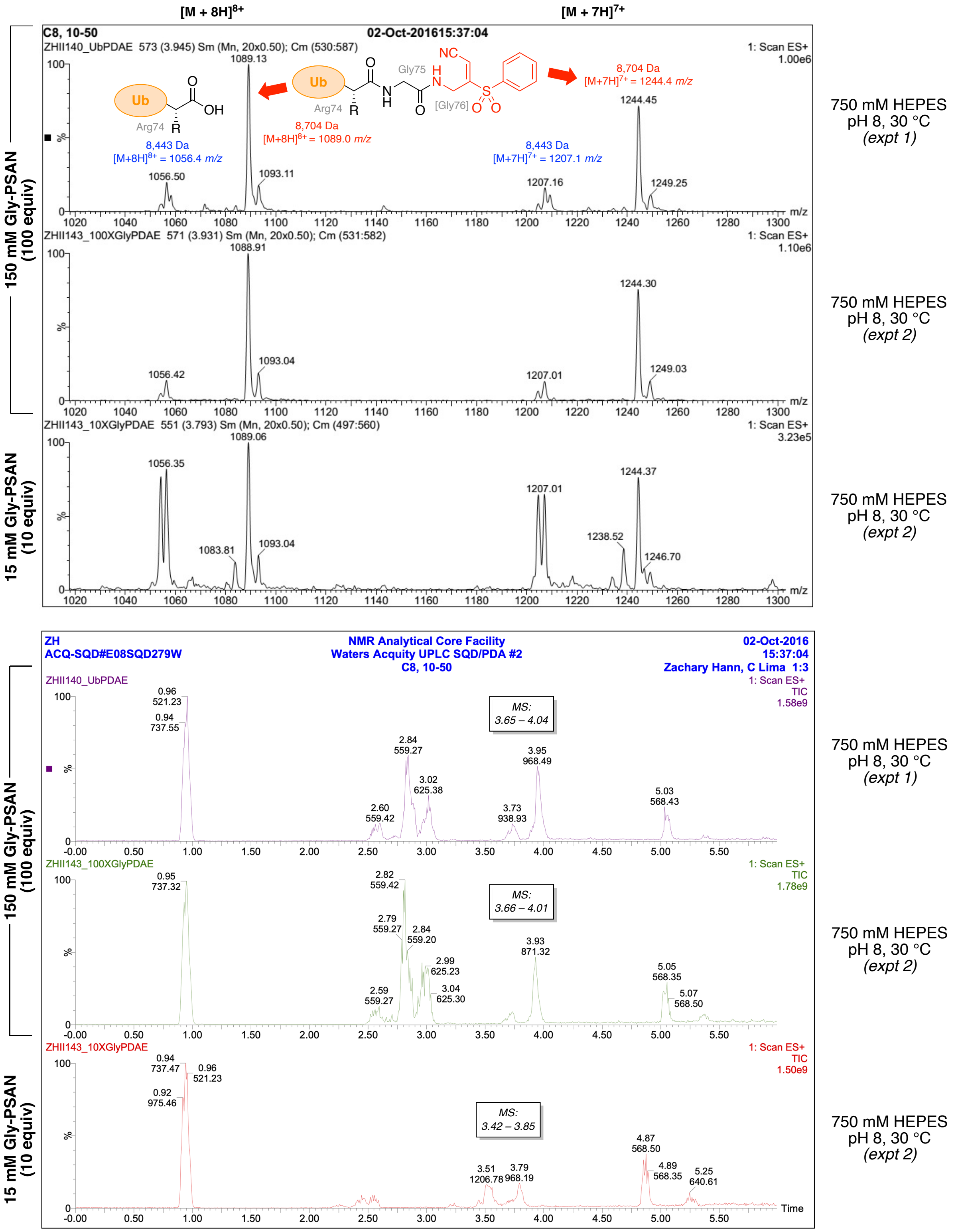

Figure S7. Aminolysis of Ub⁻² acyl azide (27) with various concentrations of Gly-PSAN (*E*-22a). Mass spectra (top) integrated over the HPLC range indicated (bottom). *Conditions*: 1.5 mM Ub⁻² hydrazide (**26**), 250 mM $NaNO_2$, 50 mM citrate, pH 3, –5 °C, 2 min; then Gly-PSAN·HCl (***E*-22a**) as indicated, 750 mM HEPES, pH 8, 10 min.

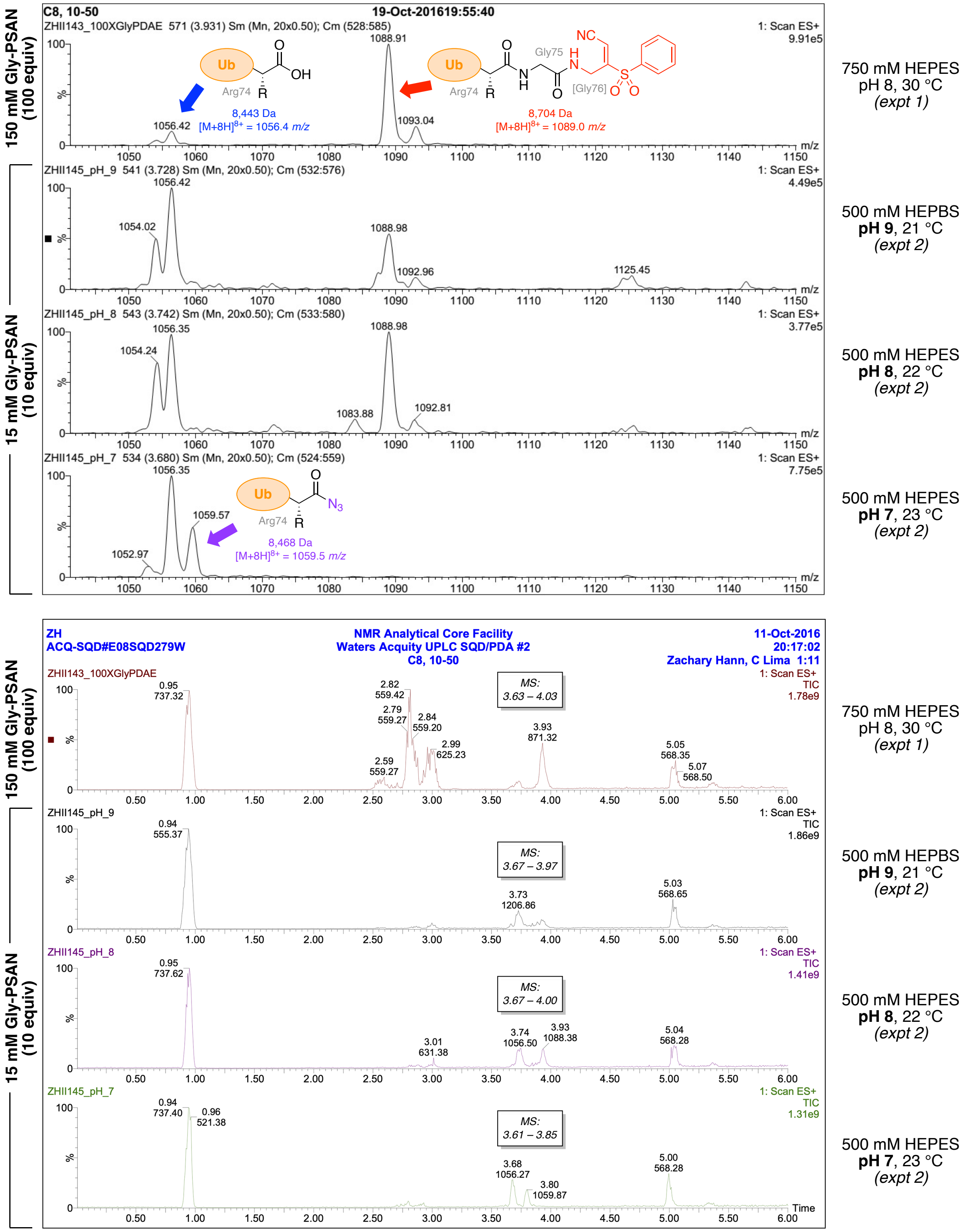

Figure S8. Aminolysis of Ub^{-2} acyl azide (27) with Gly-PSAN (*E*-22a) at various pH. Mass spectra (top) integrated over the HPLC range indicated (bottom). *Conditions*: 1.5 mM Ub^{-2} hydrazide (**26**), 250 mM $NaNO_2$, 50 mM citrate, pH 3, –5 °C, 2 min; then Gly-PSAN·HCl (***E*-22a**) as indicated, 750 mM HEPES, pH as indicated, 10 min.

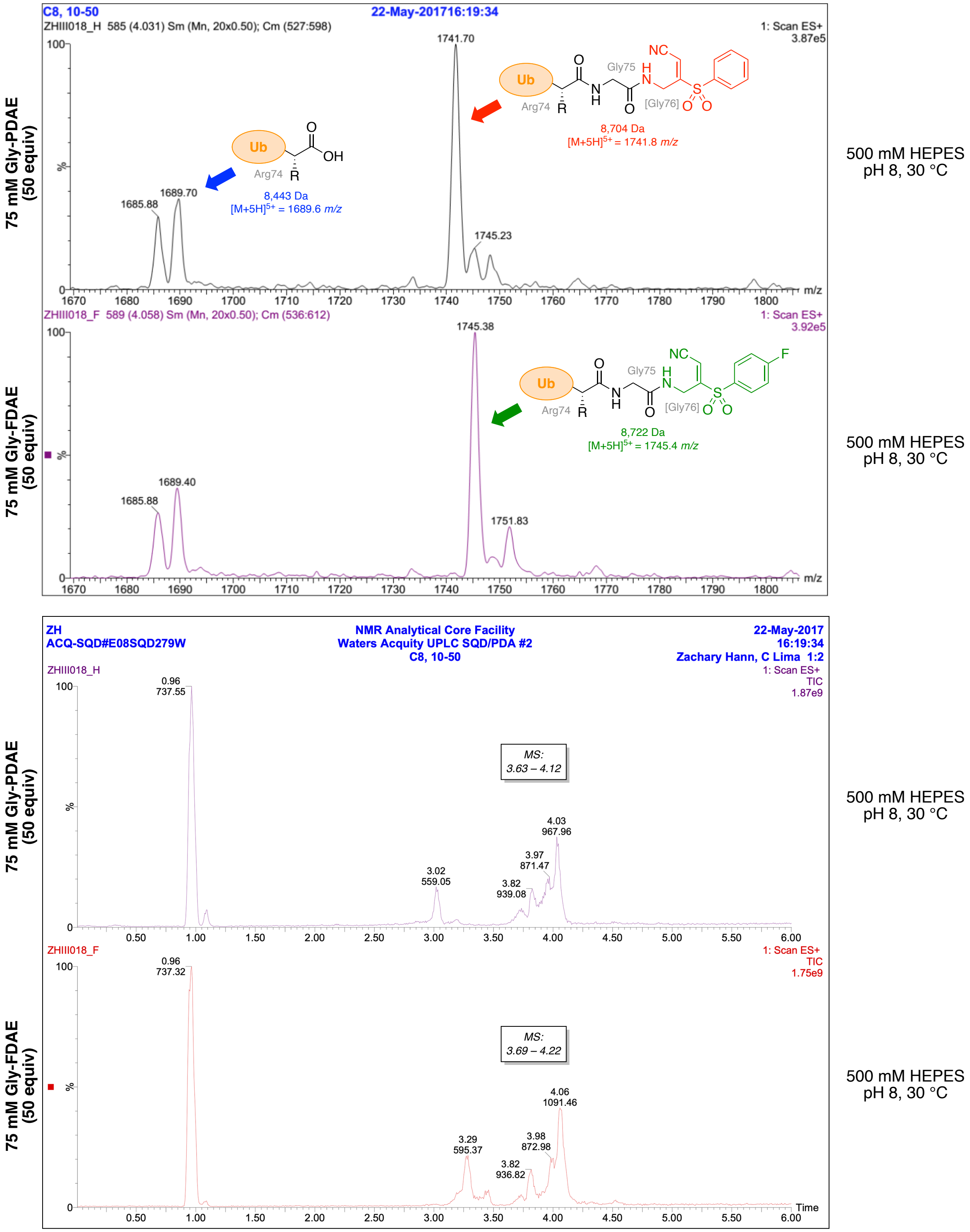

Figure S9. Aminolysis of Ub^{-2} acyl azide (27) with Gly-FSAN (22b). Mass spectra (top) integrated over the HPLC range indicated (bottom). *Conditions*: 1.5 mM Ub^{-2} hydrazide (**26**), 250 mM $NaNO_2$, 50 mM citrate, pH 3, –5 °C, 2 min; then 75 mM Gly-FSAN·HCl (**22b**), 500 mM HEPES, pH 8, 2 min.

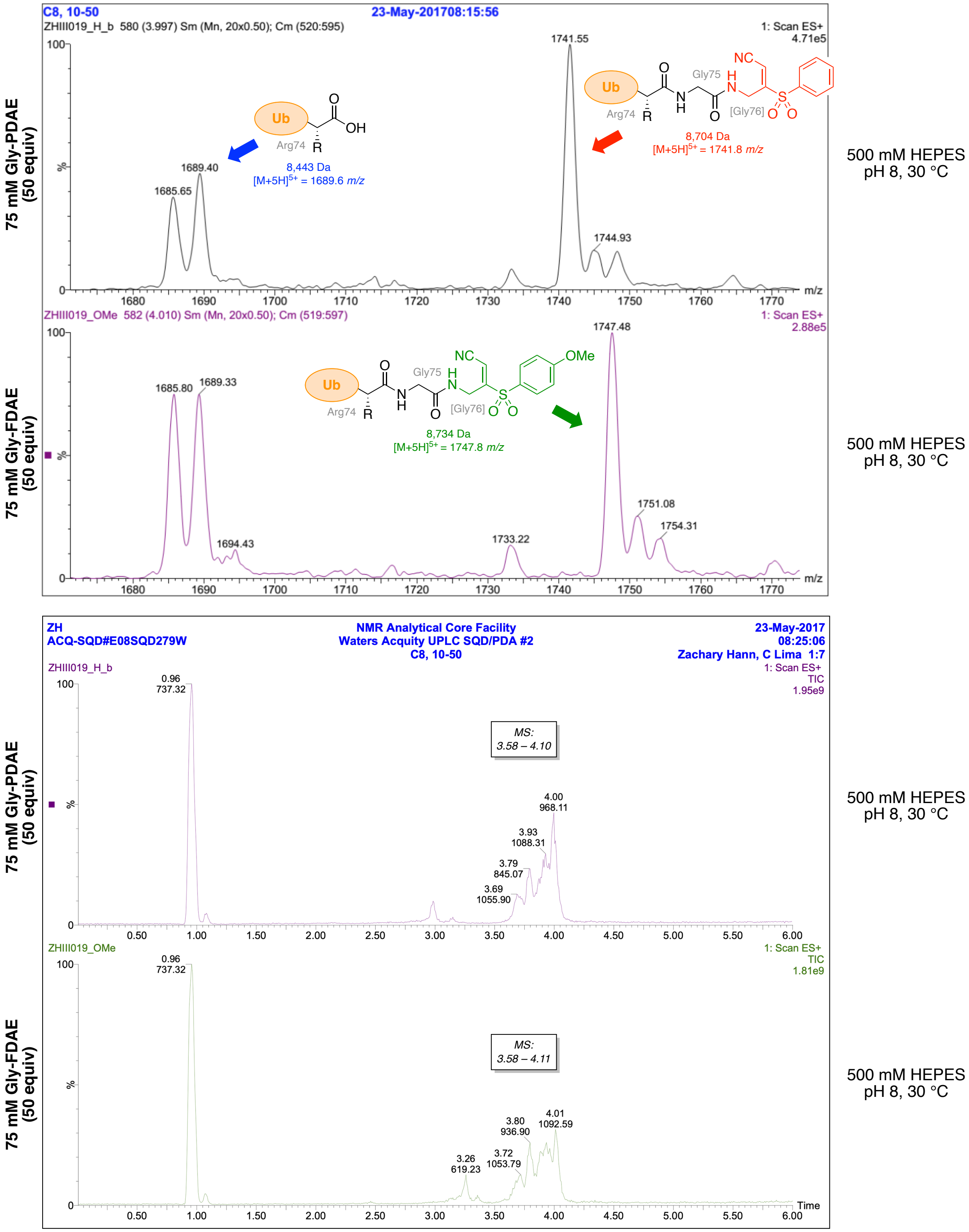

Figure S10. Aminolysis of Ub^{-2} acyl azide (27) with Gly-MSAN (22c). Mass spectra (top) integrated over the HPLC range indicated (bottom). *Conditions*: 1.5 mM Ub^{-2} hydrazide (**26**), 250 mM $NaNO_2$, 50 mM citrate, pH 3, –5 °C, 2 min; then 75 mM Gly-MSAN·HCl (**22c**), 500 mM HEPES, pH 8, 2 min.

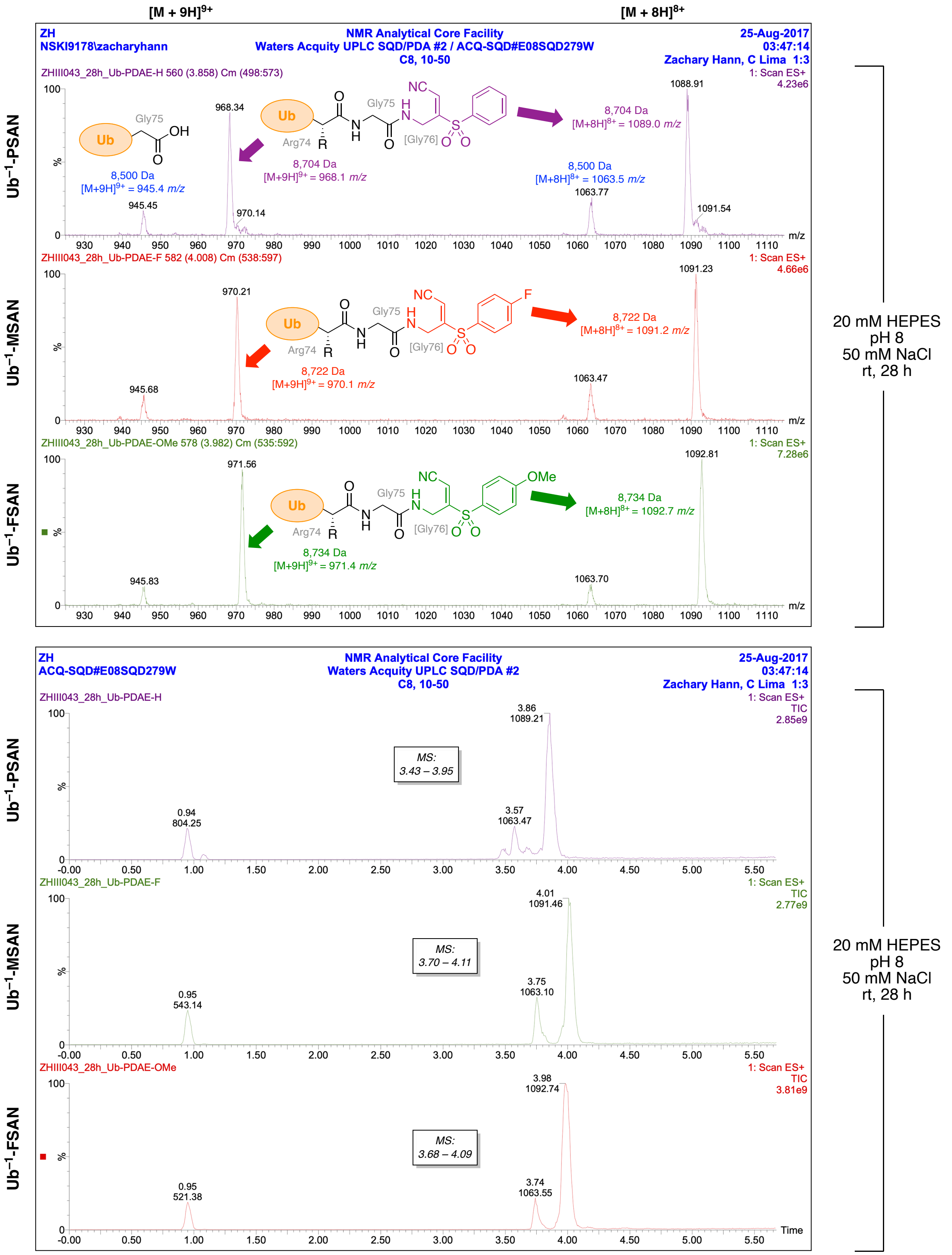

Figure S11. Stablility of Ub⁻¹-XSAN probes (18a–c). Mass spectra (top) integrated over the HPLC range indicated (bottom). *Conditions*: 400 μM Ub⁻¹-XSAN, other conditions as indicated.

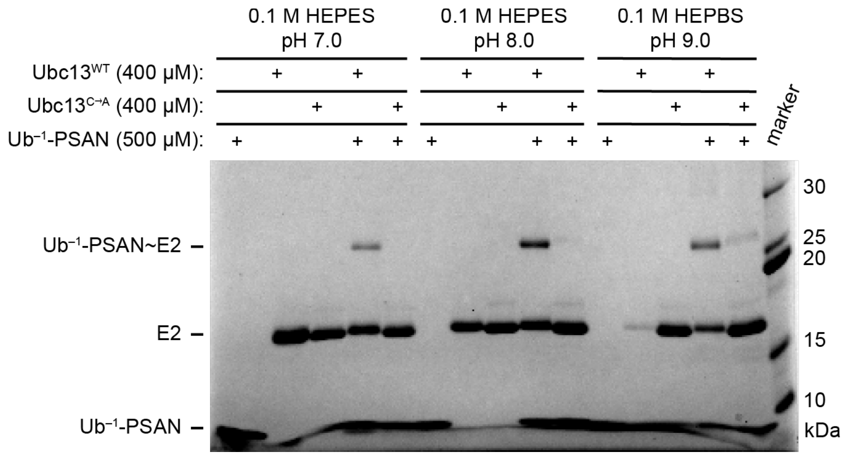

Figure S12. Conjugation of Ub⁻¹-PSAN probe (18a) and an E2 conjugating enzyme (*S. pombe* Ubc13) at various pH. *Conditions*: 500 μM Ub⁻¹-PSAN (**18a**), 400 μM E2, 100 mM HEPES, pH 7.0, rt, 12.5 h. SDS-PAGE, Coomassie stain.

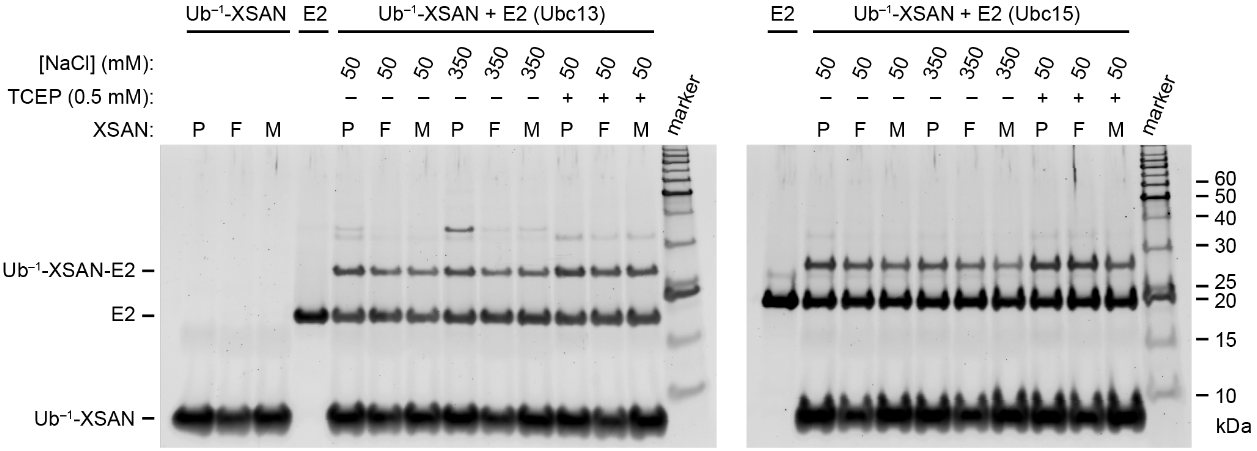

Figure S13. Conjugation of Ub⁻¹-XSAN probes (18a–c) with E2 conjugating enzymes (*S. pombe* Ubc13, left; Ubc15, right). P = Ub⁻¹-PSAN (**18a**); F = Ub⁻¹-FSAN (**18b**); M = Ub⁻¹-MSAN (**18c**). *Conditions*: 400 μM Ub⁻¹-XSAN, 200 μM E2, 20 mM HEPES, pH 8, 50 or 350 mM NaCl, ± 0.5 mM TCEP, rt, 93 h. SDS-PAGE, Sypro Ruby stain.

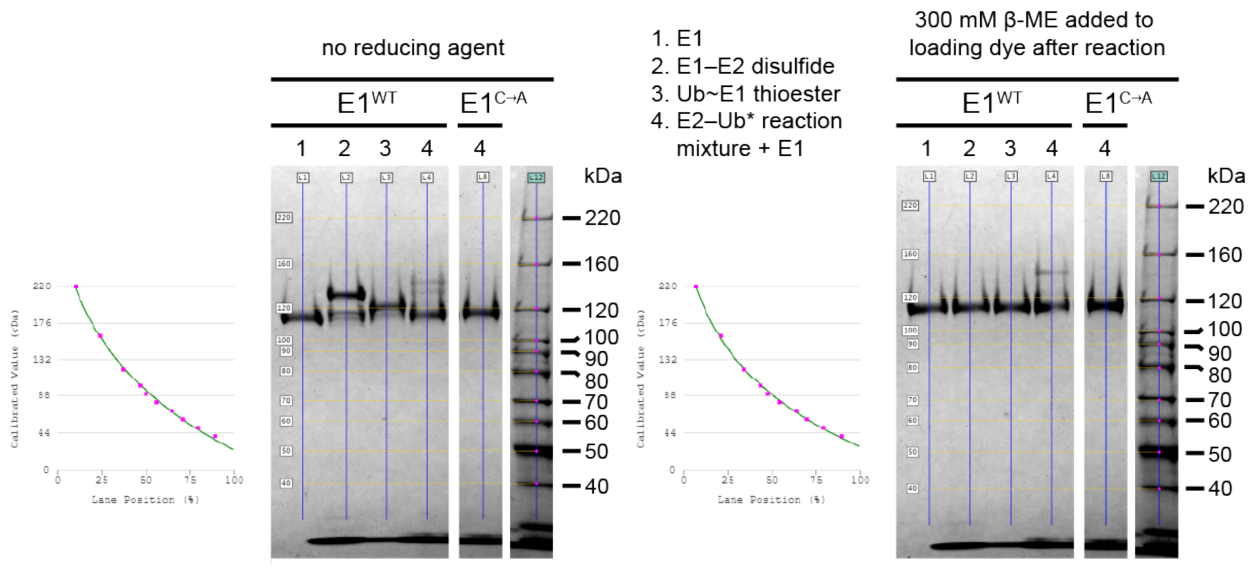

	E1	E1-E2 disulfide	Ub~E1 thioester	E1-Ub*-E2	E1
MW theoretical (kDa):	112	129	120	138	112
MW estimated from gel (kDa):	116	132	123	142	118

	E1	E1	E1	E1-Ub*-E2	E1
MW theoretical (kDa):	112	112	112	138	112
MW estimated from gel (kDa):	114	115	115	140	116

Figure S14. Estimation of molecular weights of bands in SDS-PAGE gels shown in Figure 4D. Apparent molecular weights (MW) of experimental bands were estimated based on their electrophoretic mobility relative to the MW standards ladder (Invitrogen BenchMark Protein Ladder), using the standard curve obtained with the logarithmic fit function in ImageQuantTL software (Cytiva). SDS-PAGE, Coomassie stain. (See **SECTION N** for the complete original gels).

B. MATERIALS AND METHODS

Reagents

Reagents were obtained from Aldrich Chemical (www.sigma-aldrich.com) or Acros Organics/ TCI America (www.fishersci.com) and used without further purification. Optima or HPLC grade solvents were obtained from Fisher Scientific (www.fishersci.com), degassed with Ar, and purified on a solvent drying system as described[7] unless otherwise indicated.

Reactions

All reactions were performed in flame-dried glassware under positive Ar pressure with magnetic stirring unless otherwise noted. Liquid reagents and solutions were transferred through rubber septa via syringes flushed with Ar prior to use. Cold baths were generated as follows: 0 °C, wet ice/water; –78 °C, dry ice/acetone.

Chromatography

TLC was performed on 0.25 mm E. Merck silica gel 60 F254 plates and visualized under UV light (254 nm) or by staining with potassium permanganate ($KMnO_4$), or cerium ammonium molybdenate (CAM). Silica flash chromatography was performed manually on E. Merck 230– 400 mesh silica gel 60 or on an ISCO CombiFlash Rf+ instrument with RediSep Silver silica gel normal phase columns or RediSep Gold silica gel normal phase columns with UV detection at 254 nm.

Preparative scale HPLC purification was carried out on a Waters 2545 HPLC with 2996 diode array detector using a XBridge prep C18 reverse phase column (25 cm x 19mm, 5 μm) using a flow rate of 15.0 mL/min and a gradient of 5–95% CH_3CN in 0.1% aq TFA over 20 min with UV detection at 254 nm.

Analytical LC–MS was carried out on a Waters Acuity SQD LC-MS in electrospray ionization (ESI) mode, with a 2.1*100 mL/min C18 reverse phase column (ACQUITY UPLCÒBEH, C18, 1.7 μm), 2996 diode array detector, evaporative light scattering detector (ELSD), and a ZQ4000 mass spectrometer in ESI mode using a flow rate of 0.3 mL/min and a gradient of 5–95% CH3CN in 0.1% aq TFA over 8 min. Lyophilization of larger aqueous samples was performed using a Labconco Freezone 2.5 instrument.

Analytical Instrumentation

NMR spectra were recorded on a Bruker UltraShield Plus 500 MHz Avance III NMR or UltraShield Plus 600 MHz Avance III NMR with DCH CryoProbe at 24 °C in $CDCl_3$ unless otherwise indicated. Chemical shifts are expressed in ppm relative to TMS (7^1H, 0 ppm) or solvent signals: $CDCl_3$ (^1H, 7.26 ppm; ^{13}C, 77.2 ppm), CD_2Cl_2 (^1H, 5.30 ppm; ^{13}C, 53.5 ppm), CD_3OD (^1H, 3.31 ppm; ^{13}C, 49.0 ppm), or D_2O (^1H, 4.79 ppm); coupling constants are expressed in Hz. NMR spectra were processed using Bruker TopSpin, Mnova (www.mestrelab.com/software/mnova-nmr), or nucleomatica iNMR (www.inmr.net) software. Mass spectra were obtained at the MSKCC Analytical Core Facility on a Waters Acuity SQD LC-MS or PE SCIEX API 100 by electrospray (ESI) ionization or atmospheric pressure chemical ionization (AP-CI). High resolution mass spectra were obtained on a Waters Acuity Premiere XE TOF LC-MS by electrospray ionization (ESI). X-ray crystallography analysis was carried out at the University of Toledo Instrumentation Center (http://www.utoledo.edu/nsm/ic/index.html) on a Siemens Smart CCD System. Crystal structures were visualized using CCDC Mercury software (http://www.ccdc.cam.ac.uk/products/mercury/).

Nomenclature

Atom numbers shown in the figures herein may not correspond to IUPAC nomenclature, which was used solely to name each compound. Compounds not cited in the paper are numbered herein from **S1**.

C. Synthesis of XSAN (14) and Ac-XSAN (15)

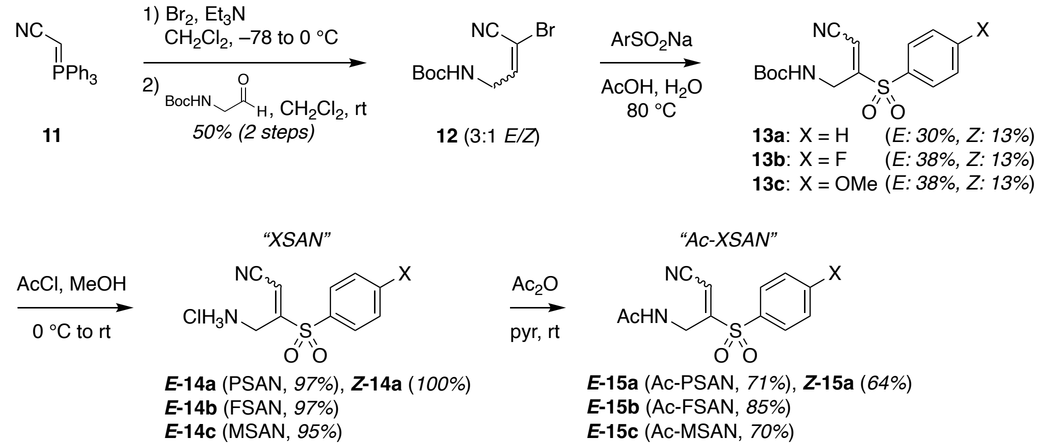

Figure S15. Synthesis of XSAN (14) and Ac-XSAN (15). Boc = *t*-butoxycarbonyl; pyr = pyridine.

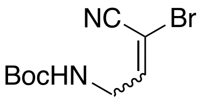

12

***tert*-Butyl (3-bromo-3-cyanoallyl)carbamate (12).** In a 100 mL roundbottom flask, ylide **11** (6.0 g, 20 mmol, 1 equiv.) was dissolved in CH_2Cl_2 (2.5 M, 0.78 mL) and triethylamine (5.5 mL, 40 mmol, 2 equiv.) was added. The mixture was cooled to –78 °C and bromine (1.0 mL, 20 mmol, 1 equiv.) was added dropwise. The mixture was warmed to 0 °C in an ice bath and stirred for 1 h. The reaction was quenched with water, and the aq phase was separated and extracted with CH_2Cl_2 (3x). The combined organic extracts were washed (brine), dried ($MgSO_4$), filtered, and concentrated by rotary evaporation. The crude bromo ylide **S1** was used directly in the next reaction without further purification.

In a 100 mL roundbottom flask, the crude bromo ylide **S6** (7.4 g, 19 mmol, 1.5 equiv.) and *N*-Boc-2-aminoacetaldehyde (2.1 g, 13 mmol, 1 equiv.) were dissolved in anhydrous CH_2Cl_2 (0.72 M, 27 mL). The mixture stirred at rt for 5 h, then concentrated via rotary evaporation. Purification by silica gel chromatography (10–20% EtOAc/hexanes) afforded the Boc-protected amine **12** (1.7 g, 50%).

The *E* and *Z* diastereomers were inseparable and were characterized as a 3:1 mixture. **TLC:** R_f 0.28 (1:4 EtOAc/Hexanes). **^{1}H-NMR** (600 MHz, $CDCl_3$) δ 6.97 (t, *J* = 5.9 Hz, 1H), 6.85 (t, *J* = 6.8 Hz, 1H), 4.86 (s, 1H), 4.03 – 3.82 (m, 2H), 1.45 (s, 9H). **^{13}C-NMR** (151 MHz, $CDCl_3$) δ 155.5, 149.8, 148.6, 115.3, 113.7, 88.6, 80.7, 41.9, 28.4. **HRMS** (ESI): *m/z* calcd for $C_9H_{13}BrN_2O_2$ $[M+H]^+$ 261.0239; found 261.0231.

General procedure for installation of the arylsulfonyl group onto α-bromoacrylonitrile

The requisite sulfinic acid sodium salts were synthesized as previously reported.[8] In a roundbottom flask, Boc-protected amine **12** (1 equiv.) and the appropriate sulfinic acid sodium salt (2 equiv.) were dissolved in 2:5 AcOH/H_2O (0.3 M substrate concentration). The solution was heated to 80 °C and stirred for 1 h, then cooled to 25 °C. The reaction was quenched by the addition of 3 M NaOH dropwise until the solution reached pH 12. The aq phase was extracted with EtOAc (3x). The combined organic extracts were washed (brine), dried ($MgSO_4$), filtered, and concentrated by rotary evaporation. Purification by silica gel chromatography (10–40% EtOAc/hexanes) afforded the Boc-protected XSAN warheads **13a–c**. The *E* and *Z* diastereomers were separable at this stage, and were characterized separately.

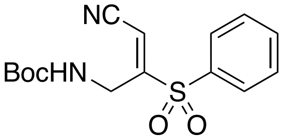

***E*-13a**

***tert*-Butyl (*E*)-(3-cyano-2-(phenylsulfonyl)allyl)carbamate (*E*-13a, (*E*)-Boc-PSAN).** Prepared from Boc-protected amine **12** and sodium benzenesulfinate as a white crystalline solid (3.7 g, 30%). **TLC:** R*f* 0.27 (1:4 EtOAc/Hexanes). **^{1}H-NMR** (600 MHz, $CDCl_3$) δ 7.89 (d, *J* = 1.5 Hz, 2H), 7.71 (t, *J* = 7.5 Hz, 1H), 7.60 (t, *J* = 7.9 Hz, 2H), 6.63 (s, 1H), 4.95 (s, 1H), 4.22 (d, *J* = 6.2 Hz, 2H), 1.36 (s, 9H). **^{13}C-NMR** (151 MHz, $CDCl_3$) δ 159.9, 154.8, 137.2, 134.9, 129.9, 128.7, 112.8, 109.1, 80.6, 39.4, 28.2. **HRMS** (ESI): *m/z* calcd for $C_{15}H_{18}N_2O_4S$ $[M+Na]^+$ 345.0885; found 345.0878.

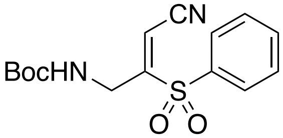

***Z*-13a**

***tert*-Butyl (*Z*)-(3-cyano-2-(phenylsulfonyl)allyl)carbamate (*Z*-13a, (*Z*)-Boc-PSAN)**. Prepared from Boc-protected amine **12** and sodium benzenesulfinate as an off-white crystalline solid (1.6 g, 13%). **TLC:** R*f* 0.12 (1:4 EtOAc/Hexanes). **^{1}H-NMR** (600 MHz, $CDCl_3$) δ 8.01 (d, *J* = 7.7 Hz, 2H), 7.75 – 7.69 (m, 1H), 7.64 – 7.57 (m, 2H), 6.09 (s, 1H), 5.15 (s, 1H), 4.11 (d, *J* = 6.4 Hz, 2H), 1.37 (s, 9H). **^{13}C-NMR** (151 MHz, $CDCl_3$) δ 159.5, 155.3, 138.0, 135.2, 129.9, 128.6, 113.0, 106.8, 81.0, 41.9, 28.3, 28.1. **HRMS** (ESI): *m/z* calcd for $C_{15}H_{18}N_2O_4S$ $[M+Na]^+$ 345.0885; found 345.0871.

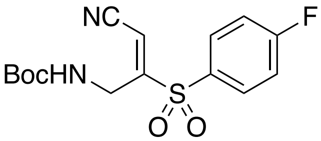

***E*-13b**

***tert*-Butyl (*E*)-(3-cyano-2-((4-fluorophenyl)sulfonyl)allyl)carbamate (*E*-13b, (*E*)-Boc-FSAN).** Prepared from Boc-protected amine **12** and sodium 4-fluorobenzenesulfinate as a white solid (0.46

g, 38%). **TLC:** R_f 0.20 (1:4 EtOAc/hexanes). **^{1}H-NMR** (600 MHz, CD_2Cl_2) δ 7.92 – 7.87 (m, 2H), 7.31 – 7.25 (m, 2H), 6.62 (s, 1H), 4.93 (s, 1H), 4.18 (dd, *J* = 6.2, 1.0 Hz, 2H), 1.32 (s, 9H). **^{13}C-NMR** (151 MHz, CD_2Cl_2) δ 166.6 (d, *J* = 258.0 Hz), 159.5, 154.8, 133.6, 131.8 (d, *J* = 10.0 Hz), 117.3 (d, *J* = 23.0 Hz), 113.0, 109.7, 80.5, 39.6, 28.0. **HRMS** (ESI): *m/z* calcd for $C_{15}H_{18}N_2O_4FS$ $[M+H]^+$ 341.0971; found 341.0963.

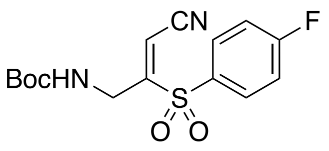

***Z*-13b**

***tert*-Butyl (*Z*)-(3-cyano-2-((4-fluorophenyl)sulfonyl)allyl)carbamate (*Z*-13b, (*Z*)-Boc-FSAN).** Prepared from Boc-protected amine **12** and sodium 4-fluorobenzenesulfinate as a white solid (0.16 g, 13%). **TLC:** R_f 0.13 (1:4 EtOAc/Hexanes). **^{1}H-NMR** (600 MHz, CD_2Cl_2) δ 8.10 – 7.99 (m, 2H), 7.35 – 7.28 (m, 2H), 6.10 (s, 1H), 4.99 (d, J = 8.3 Hz, 1H), 4.07 (dd, J = 6.3, 1.7 Hz, 2H), 1.36 (s, 9H). **^{13}C-NMR** (151 MHz, CD_2Cl_2) δ 166.8 (d, J = 258.1 Hz), 159.4, 155.1, 134.2 (d, J = 3.1 Hz), 131.7 (d, J = 10.0 Hz), 117.3 (d, J = 23.0 Hz), 113.1, 106.8, 80.8, 41.9, 28.0. **HRMS** (ESI): *m/z* calcd for $C_{15}H_{17}N_2O_4FNaS$ $[M+Na]^+$ 363.0791; found 363.0778.

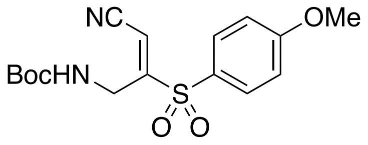

***E*-13c**

***tert*-Butyl (*E*)-(3-cyano-2-((4-methoxyphenyl)sulfonyl)allyl)carbamate (*E*-13c, (*E*)-Boc-MSAN)**. Prepared from Boc-protected amine **12** and sodium 4-methoxybenzenesulfinate as a white crystalline solid (0.76 g, 38%). **TLC:** R_f 0.35 (2:3 EtOAc/Hexanes). **^{1}H-NMR** (600 MHz, $CDCl_3$) δ 7.81 (d, *J* = 8.9 Hz, 2H), 7.04 (d, *J* = 9.0 Hz, 2H), 6.58 (s, 1H), 4.93 (s, 1H), 4.21 (dd, *J* = 6.2, 1.0 Hz, 2H), 3.89 (s, 3H), 1.38 (s, 9H). **^{13}C-NMR** (151 MHz, $CDCl_3$) δ 164.7, 160.2, 154.7, 131.0, 127.9, 115.1, 108.0, 80.5, 55.8, 39.3, 28.1. **HRMS** (ESI): *m/z* calcd for $C_{16}H_{19}N_2O_5S$ $[M]^-$ 351.1015; found 351.1002.

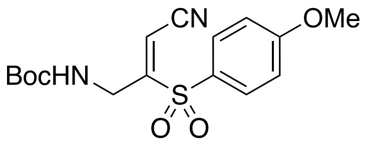

***Z*-13c**

***tert*-Butyl (*Z*)-(3-cyano-2-((4-methoxyphenyl)sulfonyl)allyl)carbamate (*Z*-13c, (*Z*)-Boc-MSAN)**. Prepared from Boc-protected amine **12** and sodium 4-methoxybenzenesulfinate as a white crystalline solid (0.26 g, 13%). **TLC:** R_f 0.25 (2:3 EtOAc/Hexanes). **^{1}H-NMR** (600 MHz, $CDCl_3$) δ 7.98 – 7.92 (m, 2H), 7.10 – 7.02 (m, 2H), 6.03 (s, 1H), 5.03 (s, 1H), 4.11 (dd, *J* = 6.4, 1.6 Hz, 2H), 3.90 (s, 4H), 1.43 (s, 9H). **^{13}C-NMR** (151 MHz, $CDCl_3$) δ 165.1, 160.1, 155.3, 131.1, 129.2, 115.2, 113.1, 105.9, 81.1, 56.0, 41.9, 28.4. **HRMS** (ESI): *m/z* calcd for $C_{16}H_{20}N_2O_5NaS$ $[M+Na]^+$ 375.0991; found 375.0994.

General procedure for Boc deprotection in anhydrous HCl (General Procedure A)

In a roundbottom flask, the Boc-protected amine **13** (1 equiv.) was dissolved in MeOH (to 0.1 M substrate concentration). The solution was cooled to 0 °C, then acetyl chloride (0.4 mL/mL MeOH) was added dropwise. The solution was warmed to rt and the reaction was stirred until completion as judged by LC-MS analysis. The mixture was concentrated by rotary evaporation and the resulting solid was dissolved into water and lyophilized to obtain the amine hydrochloride salt **14**, which was used without further purification.

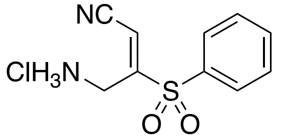

***E*-14a**

***(E)*-4-Amino-3-(phenylsulfonyl)but-2-enenitrile·HCl salt (*E*-14a, (*E*)-PSAN).** Prepared from Boc-protected PSAN amine ***E*-13a** as a fluffy off-white powder (260 mg, 97%). **^{1}H-NMR** (600 MHz, CD_3OD) δ 8.10 – 7.98 (m, 2H), 7.87 – 7.79 (m, 1H), 7.72 (ddt, *J* = 7.5, 6.3, 1.3 Hz, 2H), 7.14 (s, 1H), 3.95 (s, 1H). **^{13}C-NMR** (151 MHz, CD_3OD) δ 154.8, 137.2, 136.8, 131.4, 130.3, 117.3, 113.7, 37.8. **HRMS** (ESI): *m/z* calcd for $C_{10}H_{11}N_2O_2S$ $[M+H]^+$ 223.0541; found 223.0551.

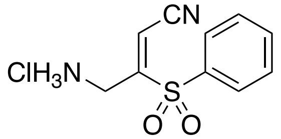

***Z*-14a**

***(Z)*-4-Amino-3-(phenylsulfonyl)but-2-enenitrile·HCl salt (*Z*-14a, (*Z*)-PSAN).** Prepared from Boc-protected PSAN amine ***Z*-13a** as a fluffy off-white powder (780 mg, quant). **^{1}H-NMR** (600 MHz, D_2O) δ 8.04 – 7.91 (m, 2H), 7.88 – 7.74 (m, 1H), 7.70 – 7.61 (m, 2H), lkk6.66 (t, *J* = 1.2 Hz, 1H), 3.94 (d, *J* = 1.3 Hz, 2H). **^{13}C-NMR** (151 MHz, D_2O) δ 152.7, 136.4, 134.9, 130.3, 128.5, 112.7, 39.3. **HRMS** (ESI): *m/z* calcd for $C_{10}H_{11}N_2O_2S$ $[M+H]^+$ 223.0541; found 223.0540.

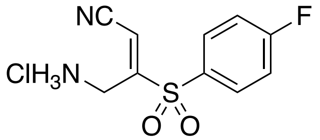

***E*-14b**

(*E*)-4-Amino-3-((4-fluorophenyl)sulfonyl)but-2-enenitrile·HCl salt (*E*-14b, (*E*)-FSAN). Prepared from Boc-protected FSAN amine ***E*-13b** as a fluffy off-white powder (220mg, 97%). **^{1}H-NMR** (600 MHz, CD_3OD) δ 8.16 – 8.09 (m, 2H), 7.54 – 7.45 (m, 2H), 7.16 (s, 1H), 3.99 (s, 2H). **^{13}C-NMR** (151 MHz, CD_3OD) δ 168.4 (d, *J* = 257.7 Hz), 154.6, 133.7 (d, *J* = 10.2 Hz), 133.3 (d, *J* = 3.0 Hz), 118.6 (d, *J* = 23.4 Hz), 117.3, 113.7, 37.9. **HRMS** (ESI): *m/z* calcd for $C_{10}H_{10}N_2O_2FS$ $[M+H]^+$ 241.0447; found 241.0452.

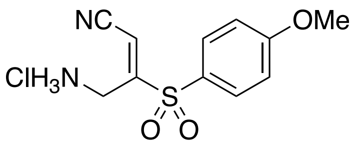

***E*-14c**

(*E*)-4-Amino-3-((4-methoxyphenyl)sulfonyl)but-2-enenitrile·HCl salt (*E*-14c, (*E*)-MSAN). Prepared from Boc-protected MSAN amine ***E*-13c** as a fluffy off-white powder (338 mg, 95%). **^{1}H-NMR** (500 MHz, CD_3OD) δ 7.95 (d, *J* = 8.9 Hz, 2H), 7.22 (d, *J* = 8.9 Hz, 2H), 7.02 (s, 1H), 3.97 – 3.92 (m, 4H). **^{13}C-NMR** (126 MHz, CD_3OD) δ 167.0, 155.3, 132.8, 127.8, 116.6, 115.9, 113.8, 56.6, 37.8. **HRMS** (ESI): *m/z* calcd for $C_{11}H_{13}N_2O_3S$ $[M+H]^+$ 253.0647; found 253.0646.

General procedure for acetylation of XSAN warheads (General Procedure B)

In a roundbottom flask were placed the XSAN amine **14** (1 equiv.) and acetic anhydride (0.1 M substrate concentration). A single drop of pyridine was added and all components of the reaction mixture dissolved. The mixture was stirred until reaction completion as judged by LC-MS analysis. Solvent was removed by rotary evaporation and the residue was purified via preparative HPLC and lyophilization to provide the Ac-XSAN **15**.

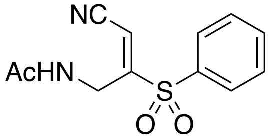

***E*-15a**

(*E*)-*N*-(3-Cyano-2-(phenylsulfonyl)allyl)acetamide (*E*-15a, (*E*)-Ac-PSAN). Prepared from PSAN amine ***E*-14a** as a white fluffy powder (11 mg, 71%). **^{1}H-NMR** (600 MHz, $CDCl_3$) δ 7.96 – 7.90 (m, 2H), 7.82 – 7.75 (m, 1H), 7.67 (t, *J* = 7.9 Hz, 2H), 6.68 (d, *J* = 1.2 Hz, 1H), 5.94 (s, 1H), 4.33 (dd, *J* = 5.9, 1.0 Hz, 2H), 1.94 (s, 3H). **^{13}C-NMR** (151 MHz, $CDCl_3$) δ 170.2, 159.1, 136.6, 135.1, 129.9, 128.7, 112.8, 109.0, 38.2, 22.6. **HRMS** (ESI): *m/z* calcd for $C_{12}H_{13}N_2O_3S$ $[M+H]^+$ 265.0647; found 265.0642.

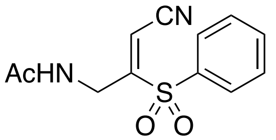

***Z*-15a**

(*Z*)-*N*-(3-Cyano-2-(phenylsulfonyl)allyl)acetamide (*Z*-15a, (*Z*)-Ac-PSAN). Prepared from PSAN amine ***Z*-14a** as a white fluffy powder (7 mg, 64%). **^{1}H-NMR** (500 MHz, $CDCl_3$) δ 8.07 – 7.98 (m, 2H), 7.80 – 7.71 (m, 1H), 7.69 – 7.60 (m, 2H), 6.24 (s, 1H), 6.19 – 6.13 (m, 1H), 4.20 (d, *J* = 6.2 Hz, 2H), 1.98 (s, 3H). **^{13}C-NMR** (126 MHz, $CDCl_3$) δ 170.6, 158.4, 137.8, 135.4, 130.1, 128.6, 112.9, 107.9, 40.6, 23.1. **HRMS** (ESI): *m/z* calcd for $C_{12}H_{12}N_2O_3NaS$ $[M+Na]^+$ 287.0466; found 287.0477.

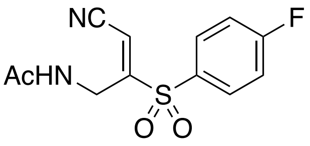

***E*-15b**

(*E*)-*N*-(3-Cyano-2-((4-fluorophenyl)sulfonyl)allyl)acetamide (*E*-15b, (*E*)-Ac-FSAN). Prepared from FSAN amine ***E*-14b** as a white fluffy powder (22 mg, 85%). **^{1}H-NMR** (600 MHz, CD_2Cl_2) δ 7.95 – 7.89 (m, 2H), 7.35 – 7.29 (m, 2H), 6.64 (t, *J* = 1.0 Hz, 1H), 5.92 (s, 1H), 4.27 (dd, *J* = 6.0, 1.1 Hz, 2H), 1.86 (s, 3H). **^{13}C-NMR** (151 MHz, CD_2Cl_2) δ 170.2, 167.0 (d, *J* = 258.3 Hz), 159.4, 132.2 (d, *J* = 10.0 Hz), 117.6 (d, *J* = 23.0 Hz), 113.3, 109.7, 38.5, 22.7. **HRMS** (ESI): *m/z* calcd for $C_{12}H_{11}N_2O_3NaSF$ $[M+Na]^+$ 305.0372; found 305.0384.

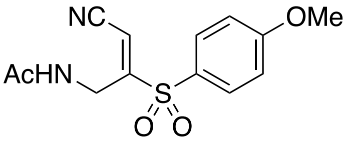

***E*-15c**

(*E*)-*N*-(3-Cyano-2-((4-methoxyphenyl)sulfonyl)allyl)acetamide (*E*-15c, (*E*)-Ac-MSAN). Prepared from MSAN amine ***E*-14c** as a white fluffy powder (10 mg, 70%). **^{1}H-NMR** (600 MHz, $CDCl_3$) δ 7.81 (d, *J* = 8.9 Hz, 2H), 7.07 (d, *J* = 9.0 Hz, 2H), 6.59 (s, 1H), 5.95 (s, 1H), 4.30 (dd, *J* = 5.9, 1.0 Hz, 2H), 3.91 (s, 3H), 1.96 (s, 3H). **^{13}C-NMR** (151 MHz, $CDCl_3$) δ 170.3, 164.9, 159.6, 131.2, 127.3, 115.2, 113.0, 108.0, 55.9, 38.1, 22.7. **HRMS** (ESI): *m/z* calcd for $C_{13}H_{15}N_2O_4S$ $[M+H]^+$ 295.0753; found 295.0748.

D. SYNTHESIS OF GLY-XSAN (22) AND AC-GLY-XSAN (23)

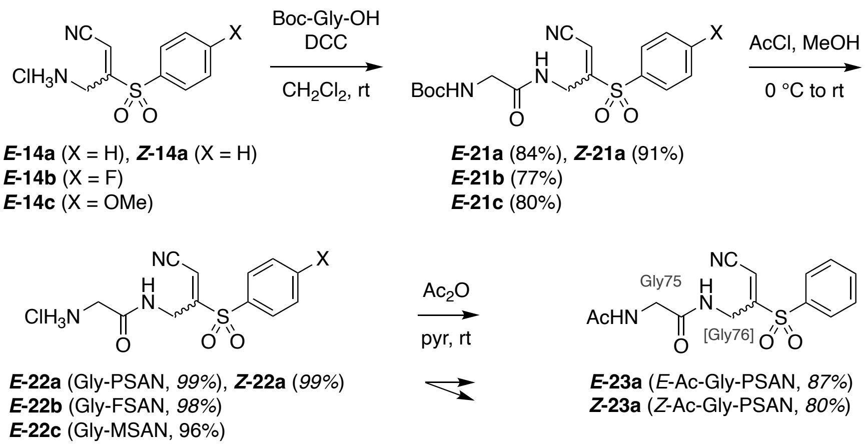

Figure S16. Synthesis of Gly-XSAN (22) and Ac-Gly-XSAN (23).

General procedure for DCC coupling of Boc-glycine

In a roundbottom flask, the XSAN amine hydrochloride **14** (1.0 equiv.), DCC (3.0 equiv.), and Boc-Gly-OH (3.0 equiv.) were dissolved in CH_2Cl_2 (0.1 M substrate concentration). The mixture was stirred until reaction completion as judged by NMR analysis. The solvent was removed by rotary evaporation, and the resulting solid dissolved in CH_3CN, then filtered to remove dicyclohexylurea (DCU). The filtrate was dried via rotary evaporation and this process was repeated twice. The crude material was then triturated with 1:2 CH_2Cl_2/hexanes to remove impurities. The resulting solid was dried to afford the Boc-Gly-XSAN warheads **21**, which were used without further purification.

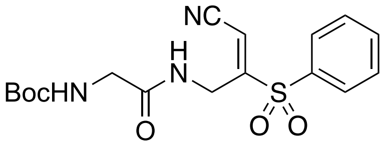

***E*-21a**

***tert*-Butyl (*E*)-(2-((3-cyano-2-(phenylsulfonyl)allyl)amino)-2-oxoethyl)carbamate (*E*-21a, (*E*)-Boc-Gly-PSAN).** Prepared from PSAN amine ***E*-14a** as a white solid (580 mg, 84%). **^{1}H-NMR** (600 MHz, CD_3OD) δ 7.95 (d, J = 7.8 Hz, 2H), 7.79 (d, J = 6.8 Hz, 1H), 7.68 (t, J = 7.8 Hz, 2H), 6.91 (s, 1H), 4.28 (s, 2H), 3.50 (s, 2H), 1.44 (s, 9H). **^{13}C-NMR** (151 MHz, CD_3OD) δ 172.7, 160.2, 158.3, 139.1, 136.1, 131.0, 129.9, 114.3, 111.0, 80.7, 44.2, 39.2, 28.7. **HRMS** (ESI): *m/z* calcd for $C_{17}H_{21}N_3O_5NaS$ $[M+Na]^+$ 402.1100; found 402.1089.

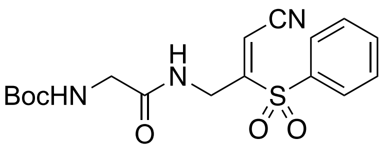

***Z*-21a**

***tert*-Butyl (*Z*)-(2-((3-cyano-2-(phenylsulfonyl)allyl)amino)-2-oxoethyl)carbamate (*Z*-21a, (*Z*)-Boc-Gly-PSAN).** Prepared from PSAN amine ***Z*-14a** as an off-white solid (73 mg, 91%). **^{1}H-NMR** (500 MHz, $CDCl_3$) δ 8.03 (d, *J* = 8.0 Hz, 2H), 7.74 (t, *J* = 7.6 Hz, 1H), 7.63 (d, *J* = 7.8 Hz, 2H), 6.95 (s, 1H), 6.18 (s, 1H), 4.24 (d, *J* = 6.2 Hz, 2H), 3.74 (d, *J* = 6.0 Hz, 2H), 1.44 (s, 9H). **^{13}C-NMR** (126 MHz, $CDCl_3$) δ 170.3, 158.5, 137.8, 135.4, 130.0, 128.7, 112.9, 107.3, 81.2, 44.8, 40.3, 28.4. **HRMS** (ESI): *m/z* calcd for $C_{17}H_{21}N_3O_5NaS$ $[M+Na]^+$ 402.1100; found 402.1100.

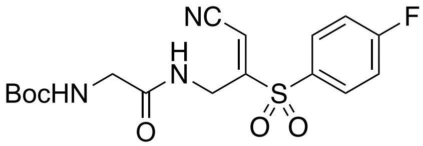

***E*-21b**

***tert*-Butyl (*E*)-(2-((3-cyano-2-((4-fluorophenyl)sulfonyl)allyl)amino)-2-oxoethyl)carbamate (*E*-21b, (*E*)-Boc-Gly-FSAN).** Prepared from FSAN amine ***E*-14b** as an off-white solid (43 mg, 77%). **^{1}H-NMR** (600 MHz, CD_2Cl_2) δ 7.97 – 7.88 (m, 2H), 7.37 – 7.26 (m, 2H), 6.74 (s, 1H), 6.64 (t, J = 1.0 Hz, 1H), 5.10 (s, 1H), 4.30 (dd, J = 6.0, 1.0 Hz, 2H), 3.72 (d, J = 6.0 Hz, 2H), 1.44 (s, 9H). **^{13}C-NMR** (151 MHz, CD_2Cl_2) δ 170.6, 167.2 (d, *J* = 258.5 Hz), 159.2, 156.6, 133.2 (d, *J* = 3.1 Hz), 132.4 (d, *J* = 10.1 Hz), 117.9 (d, *J* = 23.0 Hz), 113.4, 109.8, 38.5, 28.5. **HRMS** (ESI): *m/z* calcd for $C_{17}H_{20}N_3O_5FnaS$ $[M+Na]^+$ 420.1005; found 420.0992.

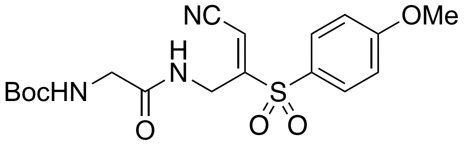

***E*-21c**

***tert*-Butyl (*E*)-(2-((3-cyano-2-((4-methoxyphenyl)sulfonyl)allyl)amino)-2-oxoethyl)carbamate (*E*-21c, (*E*)-Boc-Gly-MSAN).** Prepared from MSAN amine ***E*-14c** as an off-white solid (97 mg, 80%). **^{1}H-NMR** (500 MHz, $CDCl_3$) δ 7.85 – 7.76 (m, 2H), 7.12 – 7.00 (m, 2H), 6.72 (s, 1H), 6.58 (s, 1H), 5.05 (s, 1H), 4.32 (d, *J* = 5.9 Hz, 2H), 3.91 (s, 3H), 3.78 (d, *J* = 6.1 Hz, 2H), 1.46 (s, 9H). **^{13}C-NMR** (126 MHz, $CDCl_3$) δ 170.0, 165.1, 159.7, 131.3, 127.5, 115.4, 113.2, 107.8, 56.0, 38.2, 28.4. **HRMS** (ESI): *m/z* calcd for $C_{18}H_{23}N_3O_6NaS$ $[M+Na]^+$ 432.1205; found 432.1187.

General procedure for Boc deprotection in anhydrous HCl

The Boc-protected Gly-XSAN **21** was deprotected using General Procedure A described above to afford the Gly-XSAN amine hydrochloride salt **22**.

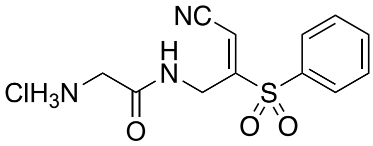

***E*-22a**

(*E*)-2-Amino-*N*-(3-cyano-2-(phenylsulfonyl)allyl)acetamide·HCl salt (*E*-22a, (*E*)-Gly-PSAN). Prepared from Boc-Gly-PSAN ***E*-21a** as an off-white fluffy powder (749 mg, 99%). **^{1}H-NMR** (600 MHz, CD_3OD) δ 8.03 – 7.94 (m, 2H), 7.86 – 7.78 (m, 1H), 7.70 (t, *J* = 7.8 Hz, 2H), 6.94 (d, *J* = 1.3 Hz, 1H), 4.32 (s, 2H), 3.53 (s, 2H). **^{13}C-NMR** (151 MHz, CD_3OD) δ 172.0, 164.4, 143.3, 140.7, 135.6, 134.5, 118.9, 115.5, 45.7, 43.6. **HRMS** (ESI): *m/z* calcd for $C_{12}H_{14}N_3O_3S$ $[M+H]^+$ 280.0756; found 280.0746.

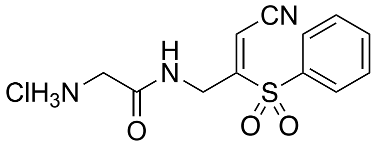

***Z*-22a**

(*Z*)-2-Amino-*N*-(3-cyano-2-(phenylsulfonyl)allyl)acetamide·HCl salt (*Z*-22a), (*Z*)-Gly-PSAN. Prepared from Boc-Gly-PSAN ***Z*-21a** as an off-white fluffy powder (291 mg, 99%). **^{1}H-NMR** (600 MHz, D_2O) δ 8.00 – 7.90 (m, 2H), 7.77 (tt, *J* = 7.5, 1.2 Hz, 1H), 7.63 (dd, *J* = 8.5, 7.5 Hz, 2H), 6.38 (t, *J* = 1.6 Hz, 1H), 4.17 (d, *J* = 1.7 Hz, 2H), 3.60 (d, *J* = 2.3 Hz, 2H). **^{13}C-NMR** (151 MHz, D_2O) δ 167.2, 156.9, 136.0, 135.9, 130.1, 128.2, 113.5, 107.7, 40.2, 40.0. **HRMS** (ESI): *m/z* calcd for $C_{12}H_{14}N_3O_3S$ $[M+H]^+$ 280.0756; found 280.0759.

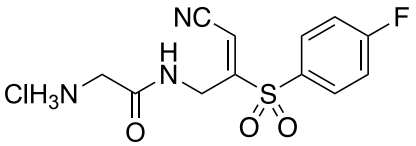

***E*-22b**

(*E*)-2-Amino-*N*-(3-cyano-2-((4-fluorophenyl)sulfonyl)allyl)acetamide·HCl salt (*E*-22b, (*E*)-Gly-FSAN). Prepared from Boc-Gly-FSAN ***E*-21b** as an off-white fluffy powder (60 mg, 98%). **^{1}H-NMR** (500 MHz, CD_3OD) δ 8.11 – 7.94 (m, 2H), 7.44 (t, *J* = 8.5 Hz, 2H), 6.93 (s, 1H), 4.32 (s, 2H), 3.59 (s, 2H). **^{13}C-NMR** (126 MHz, CD_3OD) δ 167.6, 159.9, 133.3 (d, *J* = 10.2 Hz), 118.3 (d, *J* = 23.4 Hz), 114.4, 111.0, 53.4, 41.3, 39.1. **HRMS** (ESI): *m/z* calcd for $C_{12}H_{13}N_3O_3FS$ $[M+H]^+$ 298.0662; found 298.0647.

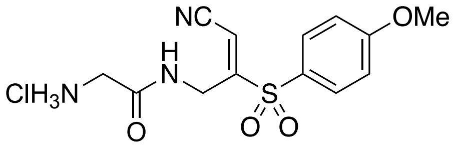

***E*-22c**

(*E*)-2-Amino-*N*-(3-cyano-2-((4-methoxyphenyl)sulfonyl)allyl)acetamide·HCl salt (*E*-22c, (*E*)-Gly-MSAN). Prepared from Boc-Gly-MSAN ***E*-21c** as an off-white fluffy powder (120 mg, 96%). **^{1}H-NMR** (600 MHz, CD_3OD) δ 8.17 (s, 1H), 7.85 – 7.77 (m, 2H), 7.13 – 7.05 (m, 2H), 3.90 (s, 2H), 3.87 (s, 3H), 3.55 (s, 2H). **^{13}C-NMR** (151 MHz, CD_3OD) δ 165.8, 164.1, 133.2, 130.4, 129.9, 115.0, 114.4, 113.2, 54.8, 40.8, 12.8. **HRMS** (ESI): *m/z* calcd for $C_{13}H_{16}N_3O_4S$ $[M+H]^+$ 310.0862; found 310.0852.

General procedure for acetylation of Gly-XSAN warheads

The Gly-PSAN amine **22** was acetylated using General Procedure B described above to afford Ac-Gly-PSAN **23**.

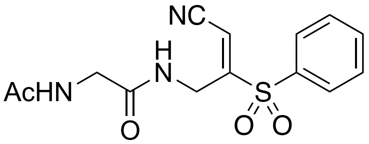

***E*-23a**

(*E*)-2-Acetamido-*N*-(3-cyano-2-(phenylsulfonyl)allyl)acetamide (*E*-23a). Prepared from Gly-PSAN amine ***E*-22a** as a white fluffy powder (112 mg, 87%). **^{1}H-NMR** (600 MHz, $CDCl_3$) δ 7.96 – 7.87 (m, 2H), 7.84 – 7.74 (m, 1H), 7.67 (dd, *J* = 8.4, 7.4 Hz, 2H), 6.79 (s, 1H), 6.68 (d, *J* = 1.1 Hz, 1H), 6.28 (s, 1H), 4.34 (dd, *J* = 6.0, 1.1 Hz, 2H), 3.89 (d, *J* = 5.5 Hz, 2H), 2.09 (s, 3H). **^{13}C-NMR** (151 MHz, $CDCl_3$) δ 171.7, 169.2, 158.8, 136.5, 135.2, 130.0, 128.7, 112.9, 108.9, 43.3, 38.2, 22.9. **HRMS** (ESI): *m/z* calcd for $C_{14}H_{15}N_3O_4SNa$ $[M+Na]^+$ 344.0681; found 344.0677.

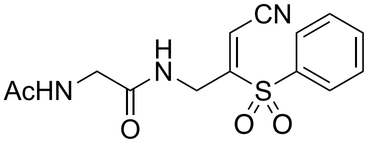

***Z*-23a**

(*Z*)-2-Acetamido-*N*-(3-cyano-2-(phenylsulfonyl)allyl)acetamide (*Z*-23a). Prepared from Gly-PSAN amine ***Z*-22a** as a white fluffy powder (98 mg, 80%). **^{1}H-NMR** (600 MHz, $CDCl_3$) δ 8.11 – 8.02 (m, 2H), 7.79 (ddt, *J* = 7.9, 7.1, 1.2 Hz, 1H), 7.71 – 7.64 (m, 2H), 6.92 (t, *J* = 5.7 Hz, 1H), 6.32 (s, 1H), 6.17 (t, *J* = 1.4 Hz, 1H), 4.27 (dd, *J* = 6.2, 1.4 Hz, 2H), 3.93 (d, *J* = 5.6 Hz, 2H), 2.12 (s, 3H). **^{13}C-NMR** (151 MHz, $CDCl_3$) δ 172.0, 169.5, 157.7, 137.5, 135.3, 130.0, 128.6, 112.6, 107.8, 43.7, 40.5, 22.8. **HRMS** (ESI): *m/z* calcd for $C_{14}H_{15}N_3O_4SNa$ $[M+Na]^+$ 344.0681; found 344.0687.

E. REACTIVITY OF AC-XSAN PROBES (15)

Aqueous stability of Ac-XSAN probes (15)

A 500 mM deuterated phosphate buffer was made from dipotassium deuterium phosphate (877 mg) in D_2O (10 mL). An internal standard of 1,4-dioxane or sodium 3-(trimethylsilyl)propionate-2,2,3,3-d_4 was added. The pD of the solution was adjusted to 8.0 (pH meter reading 7.6) with DCl in D_2O. Each warhead was dissolved in the deuterated phosphate buffer to a concentration of 1 mM. The solution was monitored with NMR for 40 min, and no decomposition of the probes was observed.

Addition of protected cysteine nucleophile to Ac-XSAN probes (15)

A 2 mM solution of each probe was prepared in the deuterated phosphate buffer. A 4 mM solution of *N*-acetyl-L-cysteine methyl ester was also prepared in the deuterated phosphate buffer. The warhead solution (0.25 mL) was mixed with the *N*-acetyl-L-cysteine methyl ester solution (0.25 mL) and the reaction was monitored by NMR for 40 min. All warheads reacted completely within 3 minutes.

F. KINETIC STUDIES OF THIOL ADDITIONS TO AC-GLY-PSAN (23)

A 1x deuterated phosphate buffered saline solution (PBS-*d*: 140 mM NaCl, 10 mM phosphate, 3 mM KCl) was made by dissolving a PBS Tablet (Sigma-Aldrich, 524650) in 100 mL of D_2O to form a 10x PBS-*d* solution, then 50 mL of 10x PBS-*d* was further diluted with 450 mL of D_2O. The pD of the solution was adjusted to 7.0 (pH meter reading 6.6) with DCl in D_2O. An internal standard of 1,4-dioxane was added.

Stock solutions of Ac-Gly-PSAN probes ***E*-23a** and ***Z*-23a** were made at 25 mM in CD_3CN. To an NMR tube was added 100 µL of the probe. In a separate vial, Ac-Cys-OMe (10, 20, or 30 equiv.) was added, followed by 400 µL of 1x PBS-*d*. This 400 µL of Ac-Cys-OMe stock solution was added to the NMR tube. The NMR tube was capped and briefly mixed then analyzed by NMR. The concentration of probe was measured between 2.5 minutes to 23.5 minutes.

The concentration of probe was plotted against time in seconds. These plots were then fitted for non-linear regression to obtain k_{obs} values. The values of k_{obs} were then plotted against initial [Ac-Cys-OMe]. A simple linear regression was used to obtain the slope, which is the second-order rate constant, k.

G. SINGLE CRYSTAL STRUCTURAL DETERMINATION OF *Z*-14A

In a 4 mL vial, 30 mg of (*Z*)-PSAN hydrochloride salt ***Z*-14a** was dissolved in 1 mL of MeOH. The open 4 mL vial was placed in a 20 mL vial containing 10 mL pentane. The 20 mL vial was capped. The solution was allowed to stand at rt undisturbed for 2 d, resulting in formation of plate-like crystals.

A clear colorless specimen of $C_{10}H_{11}ClN_2O_2S$ was used for the X-ray crystallographic analysis at the University of Toledo CNSM Instrumentation Center. The X-ray intensity data were measured (λ = 0.71073 Å).
A total of 1564 frames were collected. The total exposure time was 0.87 hours. The frames were integrated with the Bruker SAINT software package using a narrow-frame algorithm. The integration of the data using a monoclinic unit cell yielded a total of 56406 reflections to a maximum θ angle of 35.61° (0.61 Å resolution), of which 5299 were independent (average redundancy 10.645, completeness = 95.6%, R_{int} = 2.63%, R_{sig} = 1.32%) and 5180 (97.75%) were greater than $2\sigma(F^2)$. The final cell constants of a = 6.0196(4) Å, b = 6.8844(5) Å, c = 28.967(2) Å, β = 93.682(2)°, volume = 1197.95(14) Å^3, are based upon the refinement of the XYZ-centroids of 9722 reflections above 20 $\sigma(I)$ with 5.632° < 2θ < 72.52°. Data were corrected for absorption effects using the Multi-Scan method (SADABS). The ratio of minimum to maximum apparent transmission was 0.957. The calculated minimum and maximum transmission coefficients (based on crystal size) are 0.7152 and 0.7470.

The structure was solved and refined using the Bruker SHELXTL Software Package, using the space group $P2_1/c$, with Z = 4 for the formula unit, $C_{10}H_{11}ClN_2O_2S$. Small part of non-merohedral twinning (2.6%) was included in the refinement using the twin matrix (-1 0 0 0 -1 0 0.64 0 1). The final anisotropic full-matrix least-squares refinement on F^2 with 179 variables converged at R_1 = 3.49%, for the observed data and wR_2 = 8.20% for all data. The goodness-of-fit was 1.102. The largest peak in the final difference electron density synthesis was 0.524 e$^-$/Å^3 and the largest hole was -0.453 e$^-$/Å^3 with an RMS deviation of 0.069 e$^-$/Å^3. On the basis of the final model, the calculated density was 1.434 g/cm^3 and F(000), 536 e$^-$.

CCDC 2336089 contains the supplementary crystallographic data for this paper. These data can be obtained free of charge from The Cambridge Crystallographic Data Centre via www.ccdc.cam.ac.uk/data_request/cif

**Table S1. Sample and crystal data for *Z*-14a**		
**Chemical formula**	$C_{10}H_{11}ClN_2O_2S$	
**Formula weight**	258.72 g/mol	
**Temperature**	120(2) K	
**Wavelength**	0.71073 Å	
**Crystal system**	monoclinic	
**Space group**	P 2_1/c	
**Unit cell dimensions**	a = 6.0196(4) Å	α = 90°
	b = 6.8844(5) Å	β = 93.682(2)°
	c = 28.967(2) Å	γ = 90°
**Volume**	1197.95(14) Å^3	
**Z**	4	
**Density (calculated)**	1.434 g/cm^3	
**Absorption coefficient**	0.480 mm^{-1}	
**F(000)**	536	

H. CLONING, EXPRESSION AND PROTEIN PURIFICATION

Cloning of *Schizosaccharomyces pombe* Uba1, Ubc2, Ubc7, Ubc8, Ubc11, Ubc13 and $Ub^{1\text{-}75}$-intein-CBD (chitin-binding domain) were described in previous publications.[1,9] Genes encoding *S. pombe* Ubc7 and Ubc15 were each inserted into pET29b vector using NdeI and XhoI restriction sites. All Ubc7 constructs contained mutations of non-catalytic surface cysteine residues C76S, C142S, and C153N. To generate $Ub^{1\text{-}74}$-intein-CBD, residues 1-74 of the *S. pombe* Ubi5 gene were inserted into vector pTXB1 using the NdeI and SapI restriction sites.

All catalytic C→A mutations were introduced using PCR-based mutagenesis (Ubc2 C88A, Ubc7 C90A [in addition to mutations above], Ubc8 C85A, Ubc11 C113A, Ubc13, C86A, Ubc15 C90A).

S. pombe Uba1, Ubc2, Ubc8, Ubc11, Ubc13 were expressed in *E. coli* and purified as described previously,[1] and *S. pombe* Ubc7 and Ubc15 was expressed and purified using the same protocol. $Ub^{1\text{-}75}$-intein-CBD was expressed in *E. coli*, purified, and used to generate $Ub^{1\text{-}75}$-MESNa thioester (**17**, Ub^{-1} MESNa thioester), which was then converted to $Ub^{1\text{-}75}$ hydrazide (**19**, Ub^{-1} hydrazide) as described previously.[9] $Ub^{1\text{-}74}$-intein-CBD was expressed in *E. coli*, purified, and converted to $Ub^{1\text{-}74}$ MESNa thioester (**25**, Ub^{-2} MESNa thioester), then to $Ub^{1\text{-}74}$ hydrazide (**26**, Ub^{-2} hydrazide) using the same protocol.[9]

I. FORMATION OF E1-X-E2 COMPLEXES FROM BAY 11-7082 (8) OR PSAN (14)

Reaction of E2 and E1 enzymes with biselectrophiles (8, 14) (Figure 2b)

Biselectrophiles BAY 11-7082 (**8**), (*E*)-PSAN (***E*-14a**), or (*Z*)-PSAN (***Z*-14a**) were dissolved in DMSO to generate stock solutions at 100 mM. *S. pombe* Ubc13 E2 enzyme (100 µM) was derivatized by incubation with 2.5 mM biselectrophile in 20 mM Tris, pH 8, 50 mM NaCl, 2.5% DMSO at rt for 2.5 h. Samples were desalted using Micro Bio-Spin P-6 desalting columns (Bio-Rad) equilibrated with 20 mM TRIS, 50 mM NaCl. The resulting E2-X conjugates (0–20 µM) were then reacted with 50 nM *S. pombe* Uba1 E1 at rt in the same buffer, ± 50 mM DTT, at rt for 1 or 15 h. Reaction mixtures were analyzed by SDS-PAGE with Sypro Ruby staining.

J. Semisynthesis and conjugation of Ub^{-1}-XSAN probes

Attempted aminolysis of Ub^{-1} MESNa thioester (17) with amine nucleophiles (Figure 2c)

1 mM Ub^{-1} MESNa thioester (**17**) was incubated with 100 mM PSAN amine ***E*-14a** in 20 mM HEPES, pH 8, 50 mM NaCl at rt. The reaction was analyzed by UPLC-MS.

Attempted aminolysis of Ub^{-1} acyl azide (20) with amine nucleophiles (Figures 2c, S3, S4)

100 μM Ub^{-1} hydrazide (**19**) was incubated with 250 mM $NaNO_2$ in 50 mM citrate buffer, pH 3 in an ice/brine bath at –5 °C for 2 min to form the Ub^{-1} acyl azide (**20**) *in situ*. The solution was immediately added to an equal volume of 100 mM triglycine or PSAN amine (***E*-14a**) in Et_3N at the indicated concentration and resulting pH, at –5 °C, or in 750 mM HEPES at the indicated pH, at 2 °C. Reactions were analyzed by UPLC-MS.

Semisynthesis of Ub^{-1}-XSAN probes (18) from Ub^{-2} acyl azide (27) (Figures 4a, S7–S10)

3 mM Ub^{-2} hydrazide (**26**), 0.5 M $NaNO_2$, 100 mM citrate, pH 3 was incubated in an ice/brine bath for 2 min to form Ub^{-2} acyl azide (**27**). The solution was immediately added to an equal volume of 1.5 M HEPES, pH 8.0 and a 100-fold molar excess of the appropriate Gly-XSAN nucleophile (***E*-22a–c**) and incubated for at 30 °C for 2 min, then moved to ice. The resulting Ub^{-1}-XSAN probe (**18**) was purified on a Superdex 75 Increase 10/300 GL column equilibrated in 20 mM HEPES, pH 8, 50 mM NaCl, then concentrated using an Amicon Ultra centrifugal filter, flash frozen, and stored at –80 °C. Ub-MESNa thioester (**25**), Ub^{-2}-$NHNH_2$ (**26**), and the Ub^{-1}-XSAN (**18**) were distinguished via UPLC/MS using a Waters Acquity SQD UPLC-MS-PDA ultra-performance liquid chromatography/mass spectrometry instrument, using a C8 column with a water/acetonitrile gradient.

Experiments in **Figures S7–S10** were carried out using modifications to the conditions above, as indicated in the figures.

Stability of Ub^{-1}-XSAN probes (18) (Figure S11)

Ub^{-1}-PSAN probes were incubated in 20 mM HEPES, pH 8, 50 mM NaCl at rt for 28 h, then analyzed by UPLC/MS.

Reaction of E2 enzymes with Ub^{-1}-XSAN probes (18) (Figures 4b, 4c, S12, S13)

200 μM *S. pombe* Ubc13 E2 was incubated with the indicated concentration of Ub^{-1}-PSAN probe (**18a**) in 20 mM HEPES, pH 8, 50 mM NaCl at rt for 22 h, unless otherwise indicated.

For reactions comparing reactivity with different E2s, 200 μM of the indicated E2 (*S. pombe* Ubc2, Ubc7, Ubc8, Ubc11, Ubc13, or Ubc15) was incubated with 400 μM of Ub^{-1}-PSAN probe (**18a**) in 20 mM Tris, pH 8, 350 mM NaCl, 0.5 mM TCEP, at rt for 33 h.

For reactions comparing reactivity of different Ub^{-1}-XSAN probes, 200 μM of E2 (*S. pombe* Ubc13 or Ubc15) was incubated with 400 μM of the indicated Ub^{-1}-XSAN probe (**18a–c**) in 20 mM HEPES, pH 8, 50 or 350 mM NaCl, ±0.5 mM TCEP at rt for 93 h.

Samples were diluted in 4x LDS NuPAGE loading dye (Life Technologies) and analyzed by SDS-PAGE with Coomassie (Bio-Rad) staining.

Preparation of E1~Ub thioester and E1-E2 disulfide loading controls (Figure 4d)

For E1~Ub, a solution of 10 µM *S. pombe* Uba1, 50 µM Ub, 1 mM ATP, 5 mM $MgCl_2$, 20 mM Tris, pH 8, and 50 mM NaCl was incubated at rt for 1 min, then quenched in 4x LDS NuPAGE loading dye.

For E1-E2 disulfide, a solution of 10 µM *S. pombe* Uba1, 200 µM *S. pombe* Ubc13, 1 mM 2,2′-dipyridyldisulfide, 1% DMSO, 20 mM Tris, pH 8, and 50 mM NaCl was incubated at rt for 5 min, then quenched in 4x LDS NuPAGE loading dye.

Reaction of E2-Ub* probe (28) with E1 (Figure 4d)

200 µM *S. pombe* Ubc13 E2 was incubated with 500 µM Ub^{-1}-PSAN probe (**18a**) in 20 mM HEPES, pH 8, 50 mM NaCl at rt for 20 h to form the E2-Ub* probe (**28**). A sample of this reaction mixture was added to *S. pombe* Uba1 to final concentrations of 10 µM Uba1 and 60 µM total Ubc13 (including Ubc13 that had reacted with Ub^{-1}-PSAN to form E2-Ub* above) in 20 mM HEPES, pH 8, 50 mM NaCl. This mixture was incubated for 2 h then quenched in 4x LDS NuPAGE loading dye and analyzed by SDS-PAGE with Coomassie (Bio-Rad) staining.

K. SUPPORTING INFORMATION REFERENCES

(1) Olsen, S. K.; Lima, C. D. "Structure of a ubiquitin E1-E2 complex: insights to E1-E2 thioester transfer." *Mol. Cell* **2013**, *49*, 884-896.

(2) Stanley, M.; Han, C.; Knebel, A.; Murphy, P.; Shpiro, N.; Virdee, S. "Orthogonal thiol functionalization at a single atomic center for profiling transthiolation activity of E1 activating enzymes." *ACS Chem. Biol.* **2015**, *10*, 1542-1554.

(3) Pao, K. C.; Stanley, M.; Han, C.; Lai, Y. C.; Murphy, P.; Balk, K.; Wood, N. T.; Corti, O.; Corvol, J. C.; Muqit, M. M.; Virdee, S. "Probes of ubiquitin E3 ligases enable systematic dissection of parkin activation." *Nat. Chem. Biol.* **2016**, *12*, 324-331.

(4) Hehl, L. A.; Horn-Ghetko, D.; Prabu, J. R.; Vollrath, R.; Vu, D. T.; Pérez Berrocal, D. A.; Mulder, M. P. C.; van der Heden van Noort, G. J.; Schulman, B. A. "Structural snapshots along K48-linked ubiquitin chain formation by the HECT E3 UBR5." *Nat. Chem. Biol.* **2023**.

(5) Horn-Ghetko, D.; Krist, D. T.; Prabu, J. R.; Baek, K.; Mulder, M. P. C.; Klügel, M.; Scott, D. C.; Ovaa, H.; Kleiger, G.; Schulman, B. A. "Ubiquitin ligation to F-box protein targets by SCF–RBR E3–E3 super-assembly." *Nat. Chem. Biol.* **2021**, *590*, 671-676.

(6) Mao, J.; Ai, H.; Wu, X.; Zheng, Q.; Cai, H.; Liang, L.; Tong, Z.; Pan, M.; Liu, L. "Structural visualization of HECT-E3 ufd4 accepting and transferring ubiquitin to form K29/K48-branched polyubiquitination on N-degron." *bioRxiv* **2023**, 2023.2005.2023.542033.

(7) Pangborn, A. B.; Giardello, M. A.; Grubbs, R. H.; Rosen, R. K.; Timmers, F. J. "Safe and convenient procedure for solvent purification." *Organometallics* **1996**, *15*, 1518–1520.

(8) Zhou, B.; Chen, W.; Yang, Y.; Yang, Y.; Deng, G.; Liang, Y. "A radical cyclization cascade of 2-alkynylbenzonitriles with sodium arylsulfinates." *Org. Biomol. Chem.* **2018**, *16*, 7959-7963.

(9) Hann, Z. S.; Ji, C.; Olsen, S. K.; Lu, X.; Lux, M. C.; Tan, D. S.; Lima, C. D. "Structural basis for adenylation and thioester bond formation in the ubiquitin E1." *Proc. Natl. Acad. Sci. USA* **2019**, *116*, 15475-15484.

L. ^{1}H-NMR AND ^{13}C-NMR SPECTRA

1. Synthesis of XSAN (14) and Ac-XSAN (15) **S35**

a. Boc-protected amine **12** S35
b. (*E*)-Boc-PSAN, ***E*-13a** S36
c. (*Z*)-Boc-PSAN, ***Z*-13a** S37
d. (*E*)-Boc-FSAN, ***E*-13b** S38
e. (*Z*)-Boc-FSAN, ***Z*-13b** S39
f. (*E*)-Boc-MSAN, ***E*-13c** S40
g. (*Z*)-Boc-MSAN, ***Z*-13c** S41
h. (*E*)-PSAN, ***E*-14a** S42
i. (*Z*)-PSAN, ***Z*-14a** S43
j. (*E*)-FSAN, ***E*-14b** S44
k. (*E*)-MSAN, ***E*-14c** S45
l. (*E*)-Ac-PSAN, ***E*-15a** S46
m. (*Z*)-Ac-PSAN, ***Z*-15a** S47
n. (*E*)-Ac-FSAN, ***E*-15b** S48
o. (*E*)-Ac-MSAN, ***E*-15c** S49

2. Synthesis of Gly-XSAN (22) and Ac-Gly-XSAN (23) **S50**

a. (*E*)-Boc-Gly-PSAN, ***E*-21a** S50
b. (*Z*)-Boc-Gly-PSAN, ***Z*-21a** S51
c. (*E*)-Boc-Gly-FSAN, ***E*-21b** S52
d. (*E*)-Boc-Gly-MSAN, ***E*-21c** S53
e. (*E*)-Gly-PSAN, ***E*-22a** S54
f. (*Z*)-Gly-PSAN, ***Z*-22a** S55
g. (*E*)-Gly-FSAN, ***E*-22b** S56
h. (*E*)-Gly-MSAN, ***E*-22c** S57
i. (*E*)-Ac-Gly-PSAN, ***E*-23a** S58
j. (*Z*)-Ac-Gly-PSAN, ***Z*-23a** S59

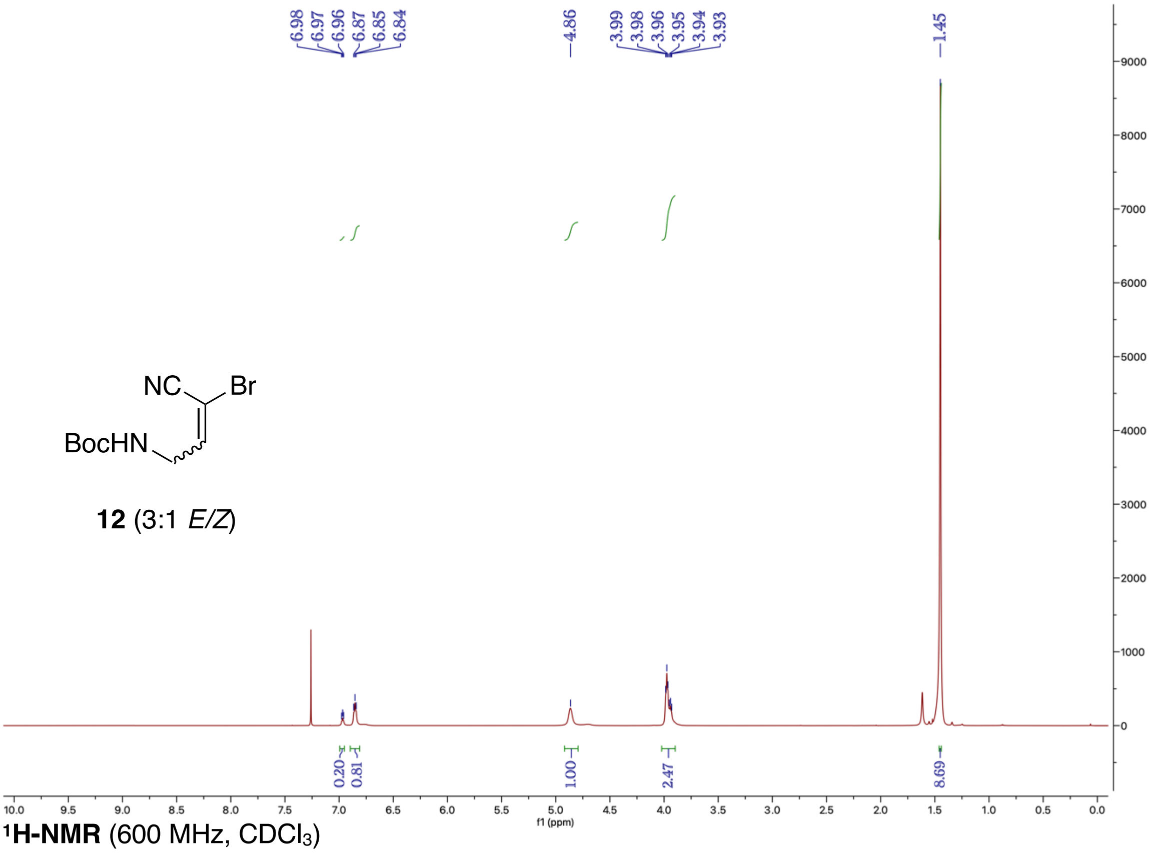

^{1}**H-NMR** (600 MHz, $CDCl_3$)

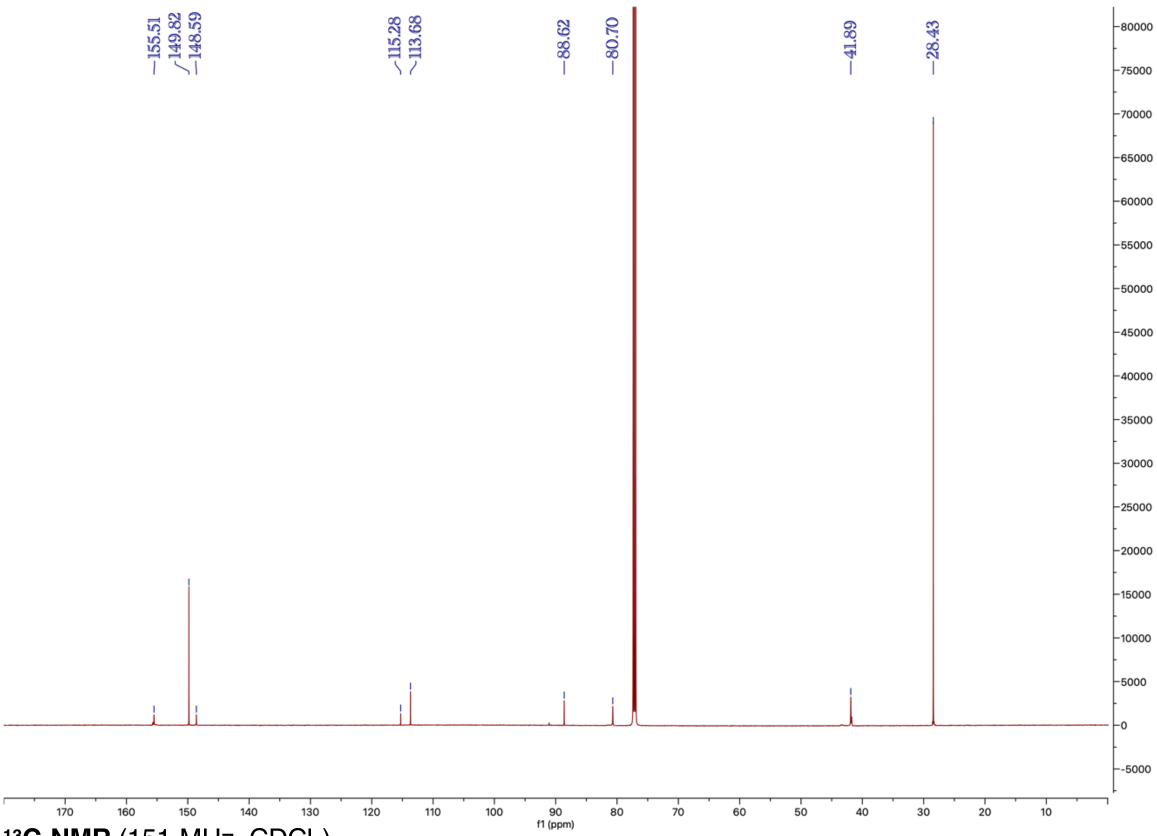

^{13}**C-NMR** (151 MHz, $CDCl_3$)

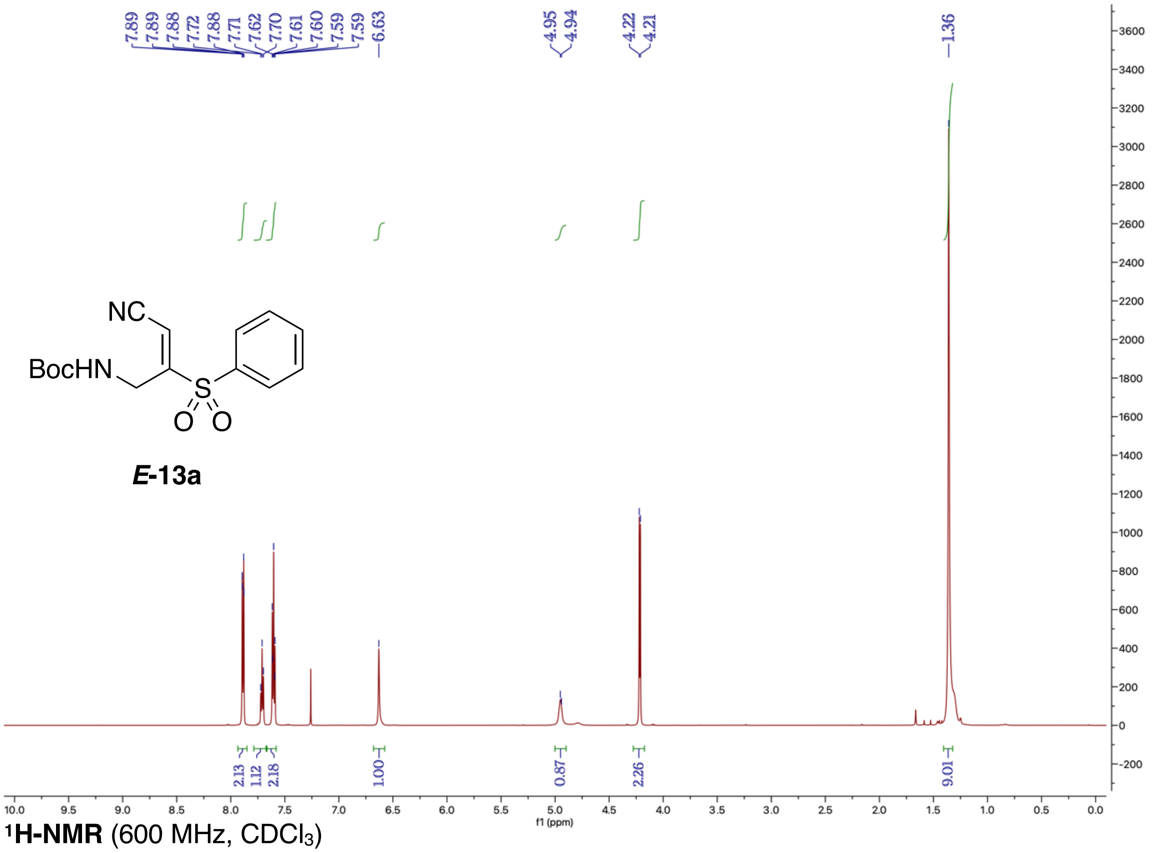

^{1}H-NMR (600 MHz, $CDCl_3$)

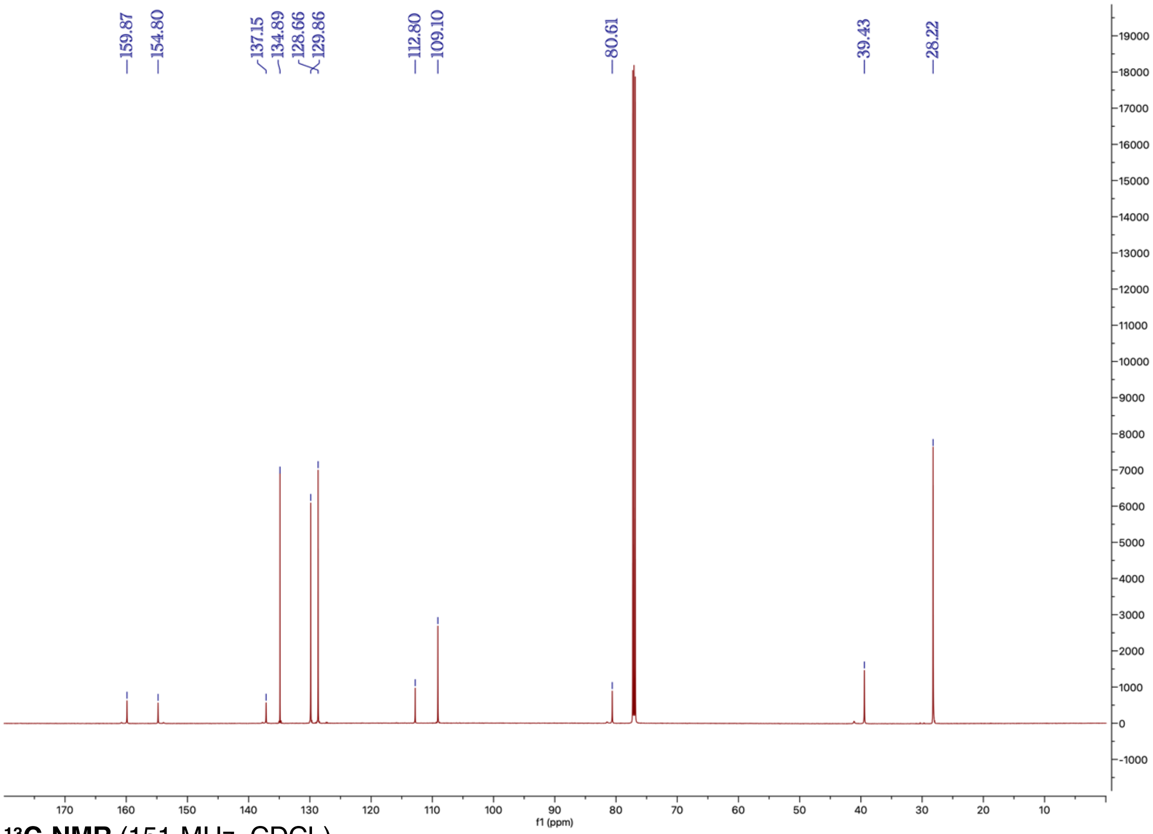

^{13}C-NMR (151 MHz, $CDCl_3$)

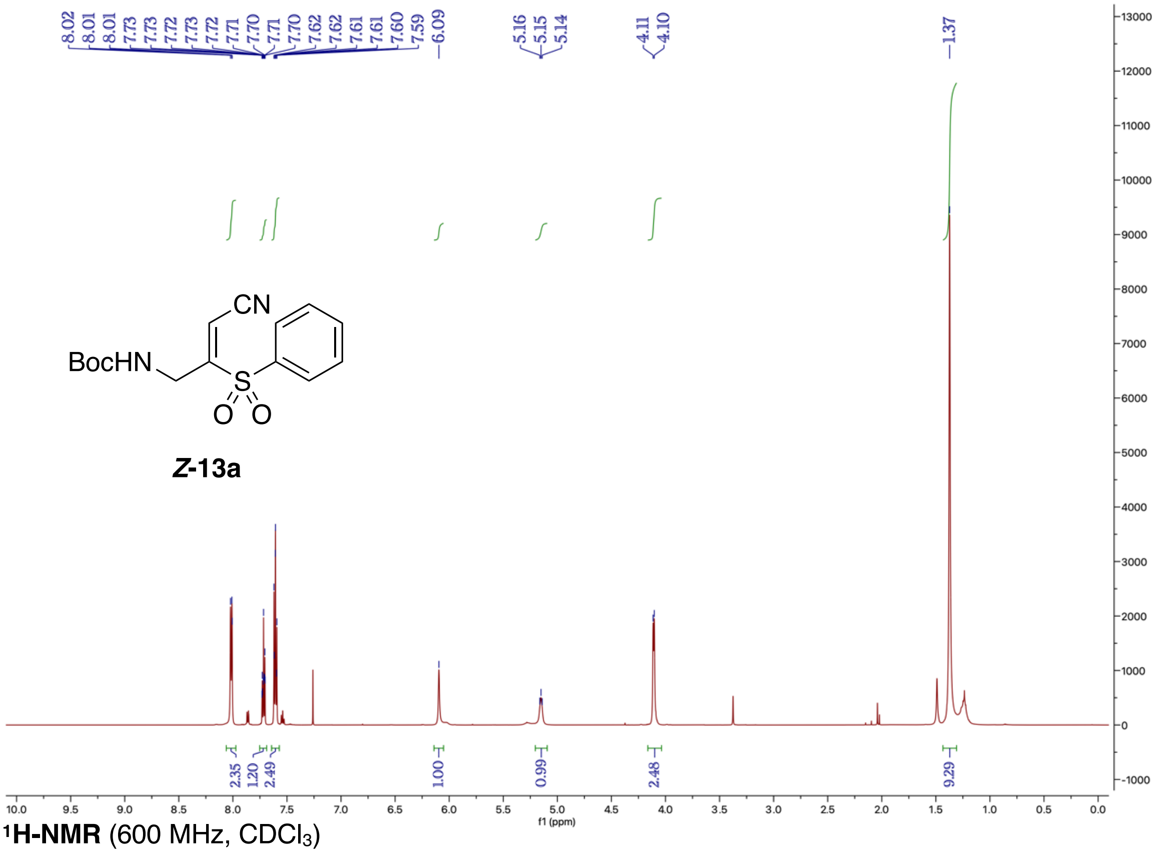

¹H-NMR (600 MHz, $CDCl_3$)

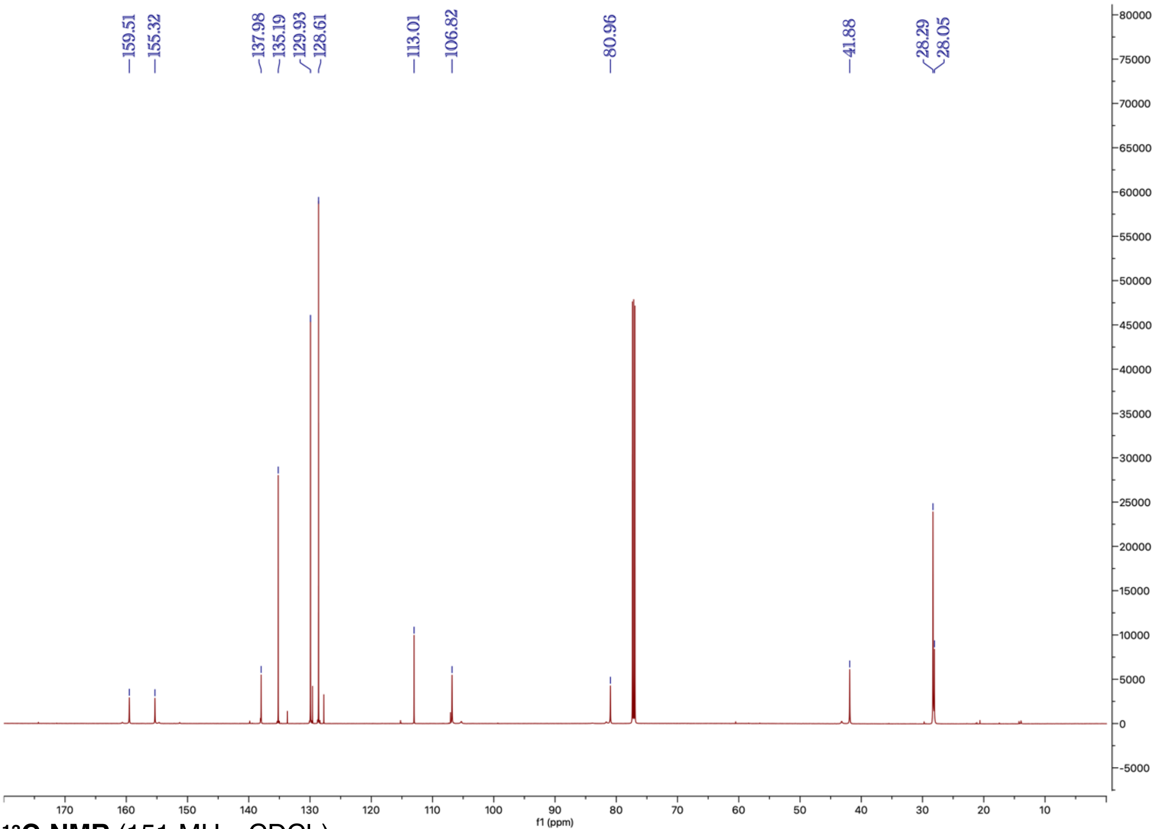

¹³C-NMR (151 MHz, $CDCl_3$)

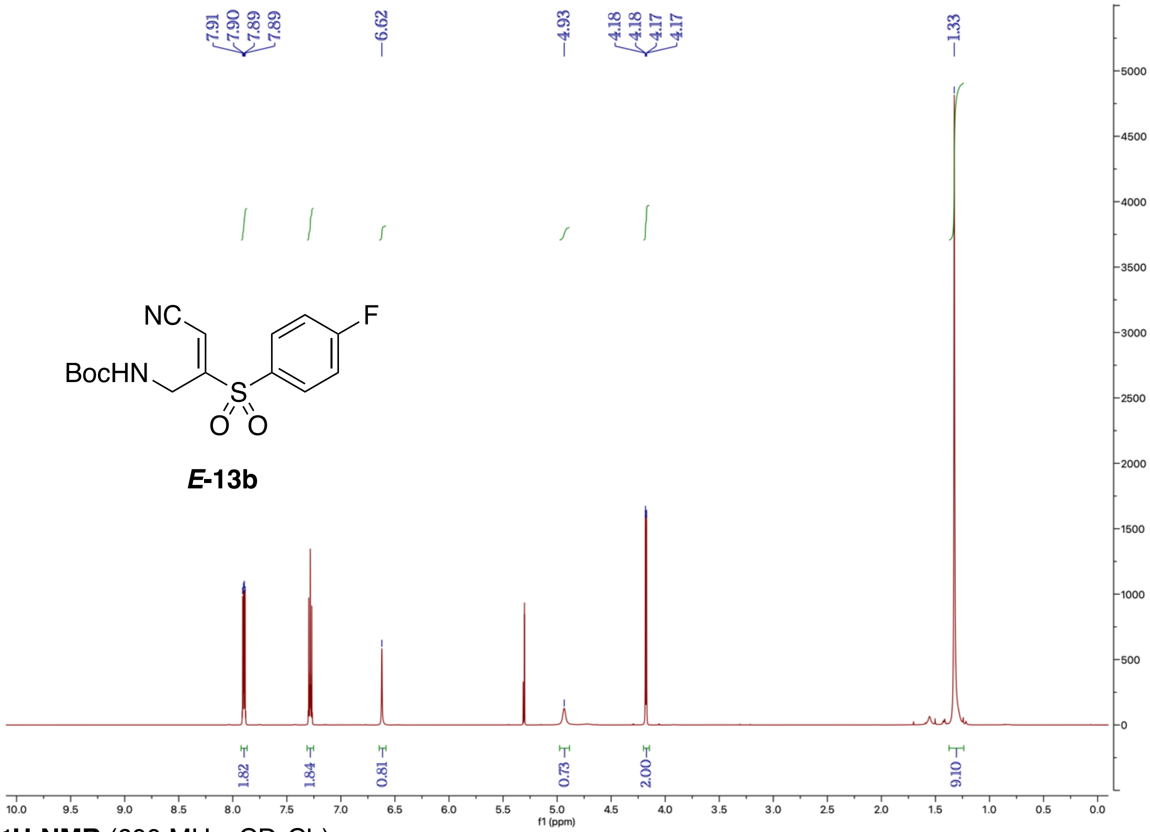

^{1}**H-NMR** (600 MHz, CD_2Cl_2)

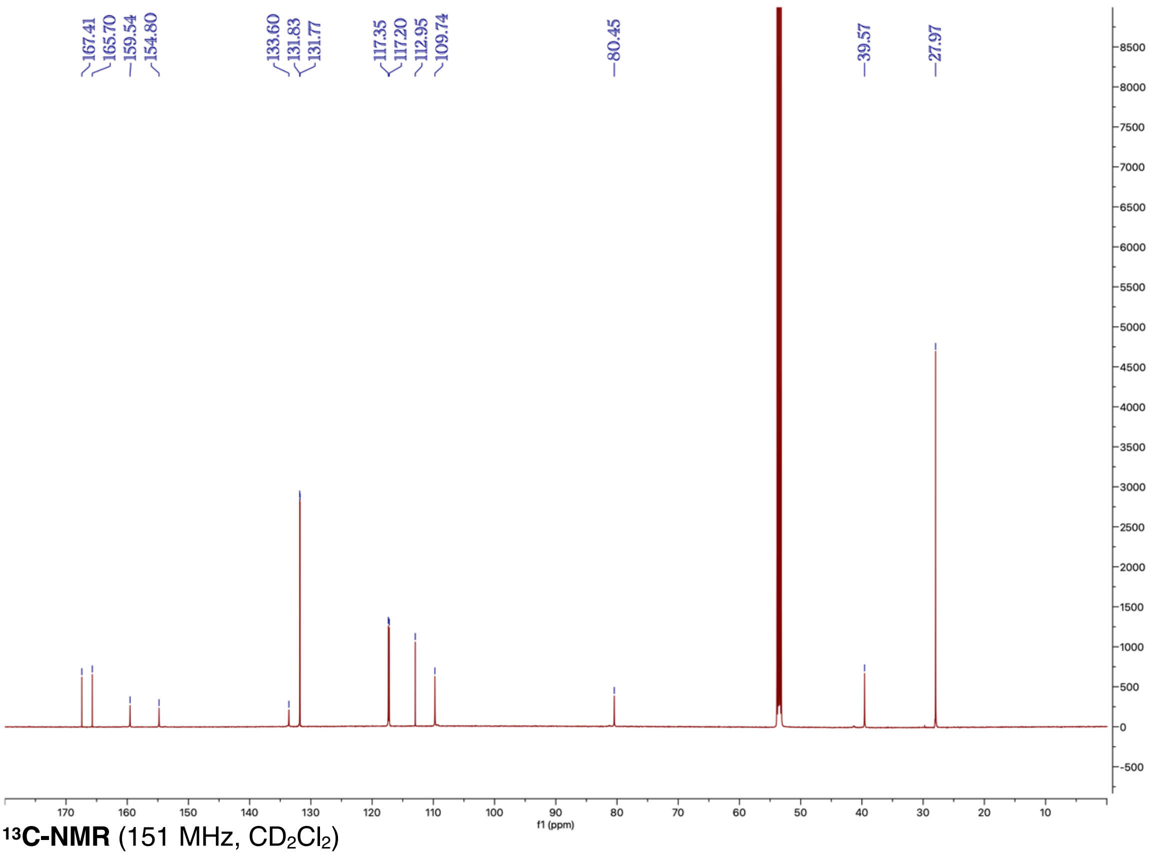

^{13}**C-NMR** (151 MHz, CD_2Cl_2)

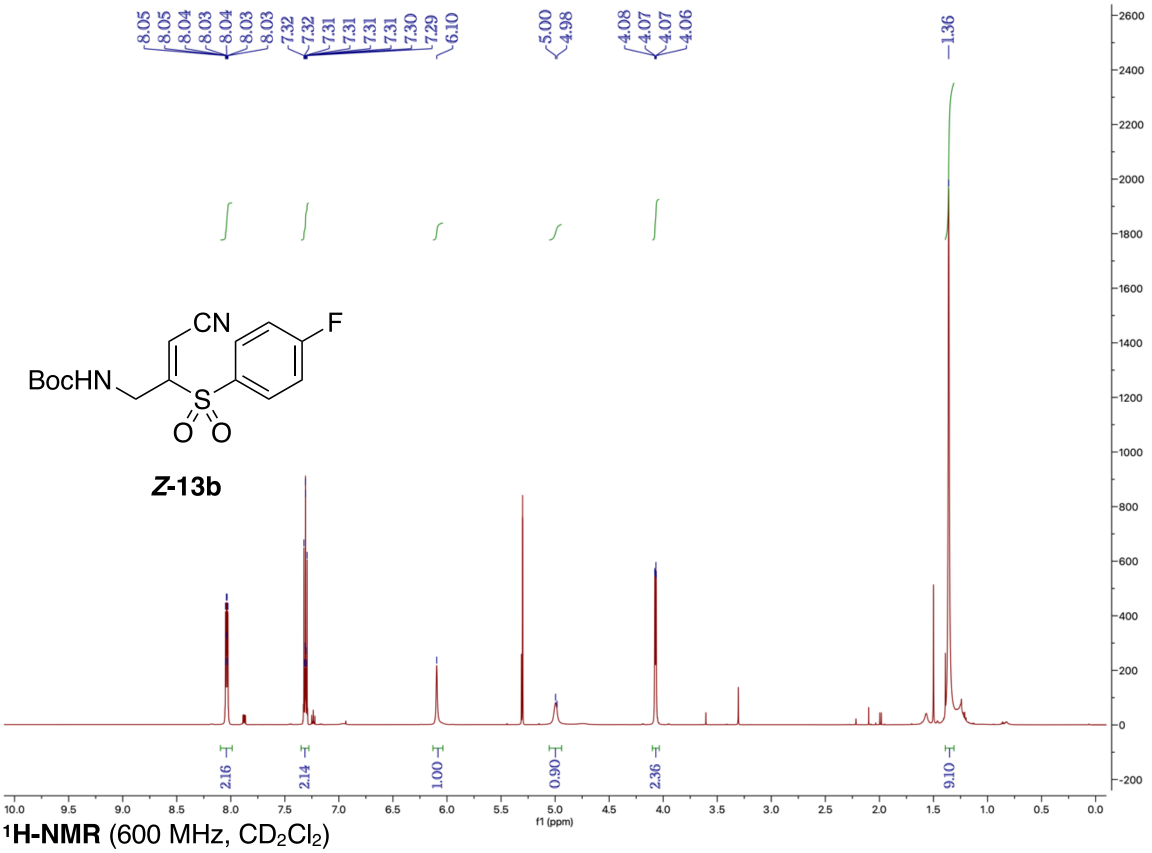

***Z*-13b**

^{1}H-NMR (600 MHz, CD_2Cl_2)

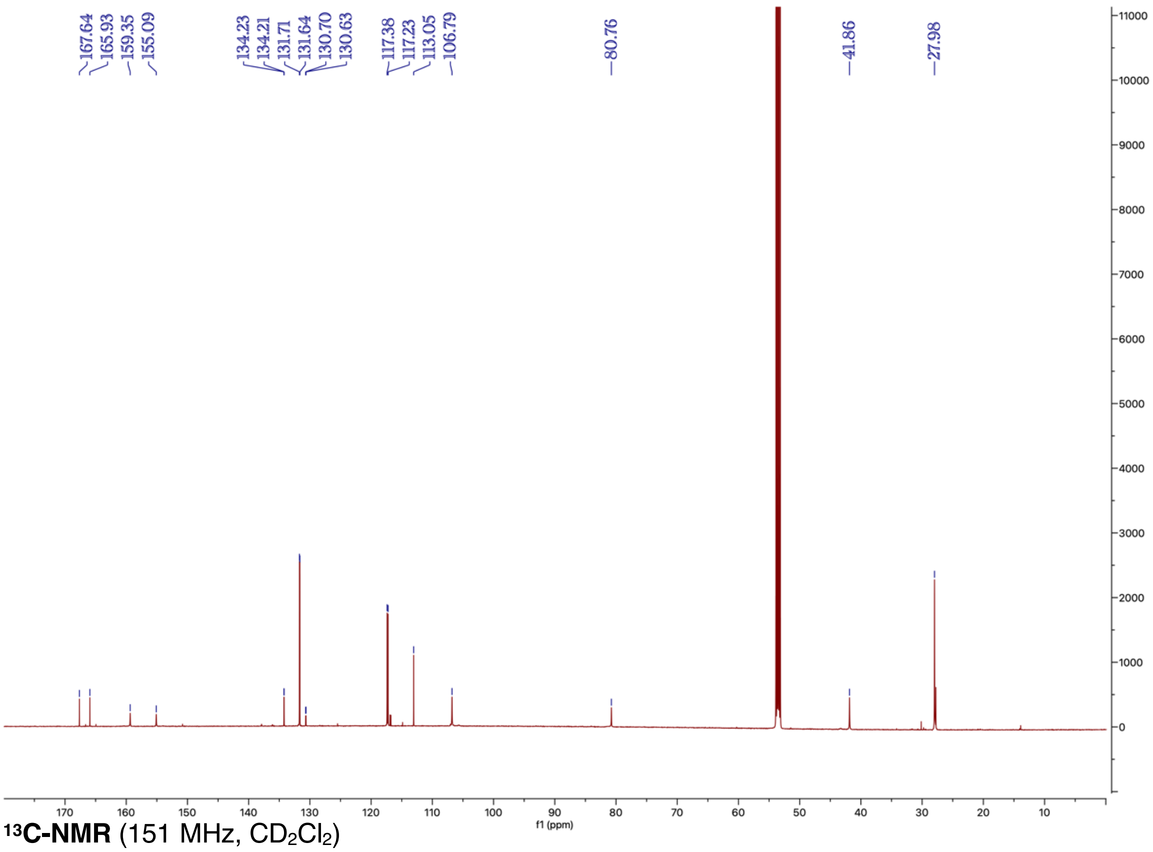

^{13}C-NMR (151 MHz, CD_2Cl_2)

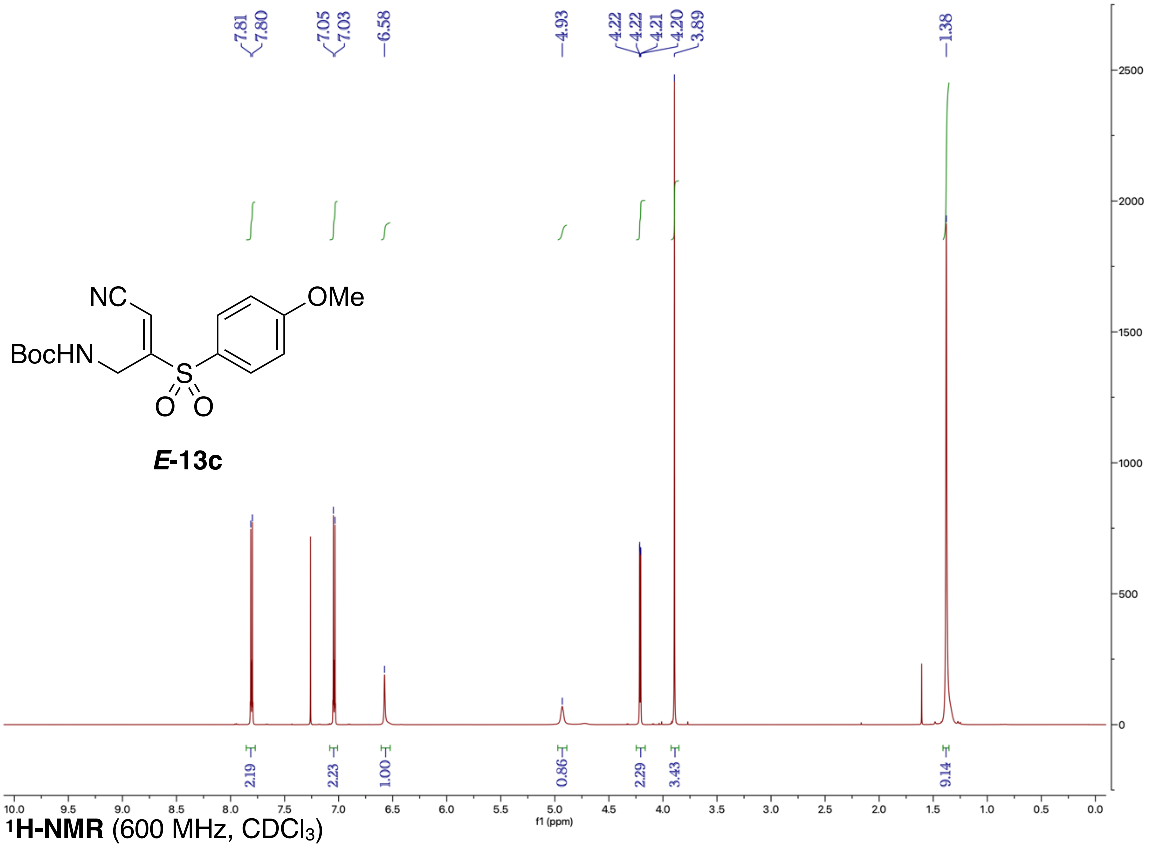

^{1}**H-NMR** (600 MHz, $CDCl_3$)

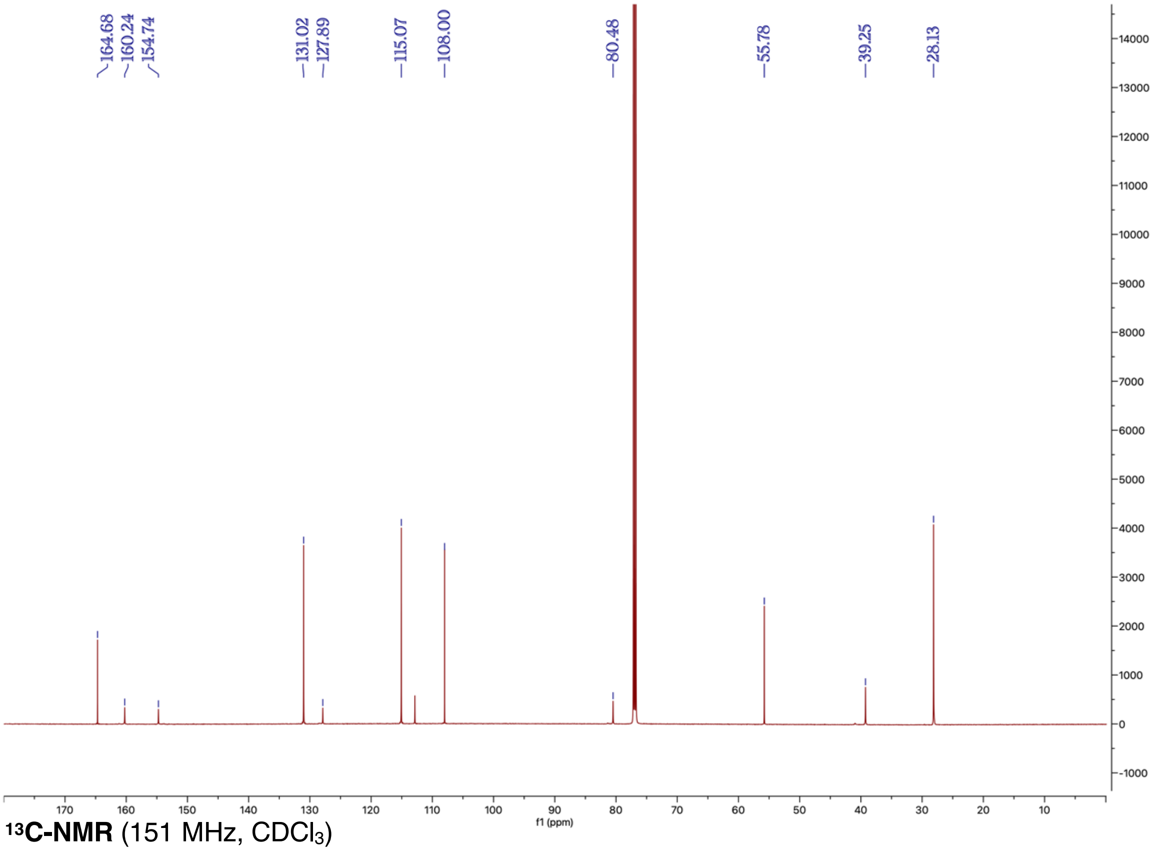

^{13}**C-NMR** (151 MHz, $CDCl_3$)

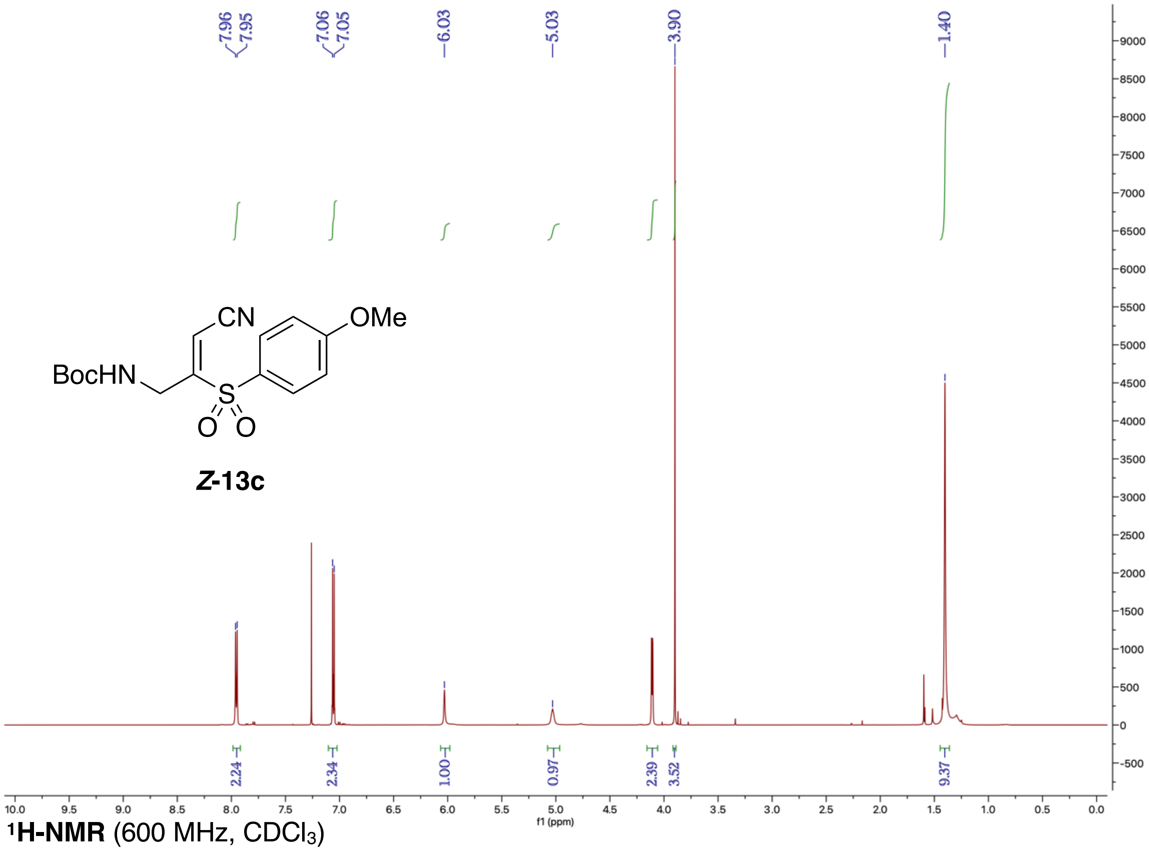

^{1}**H-NMR** (600 MHz, $CDCl_3$)

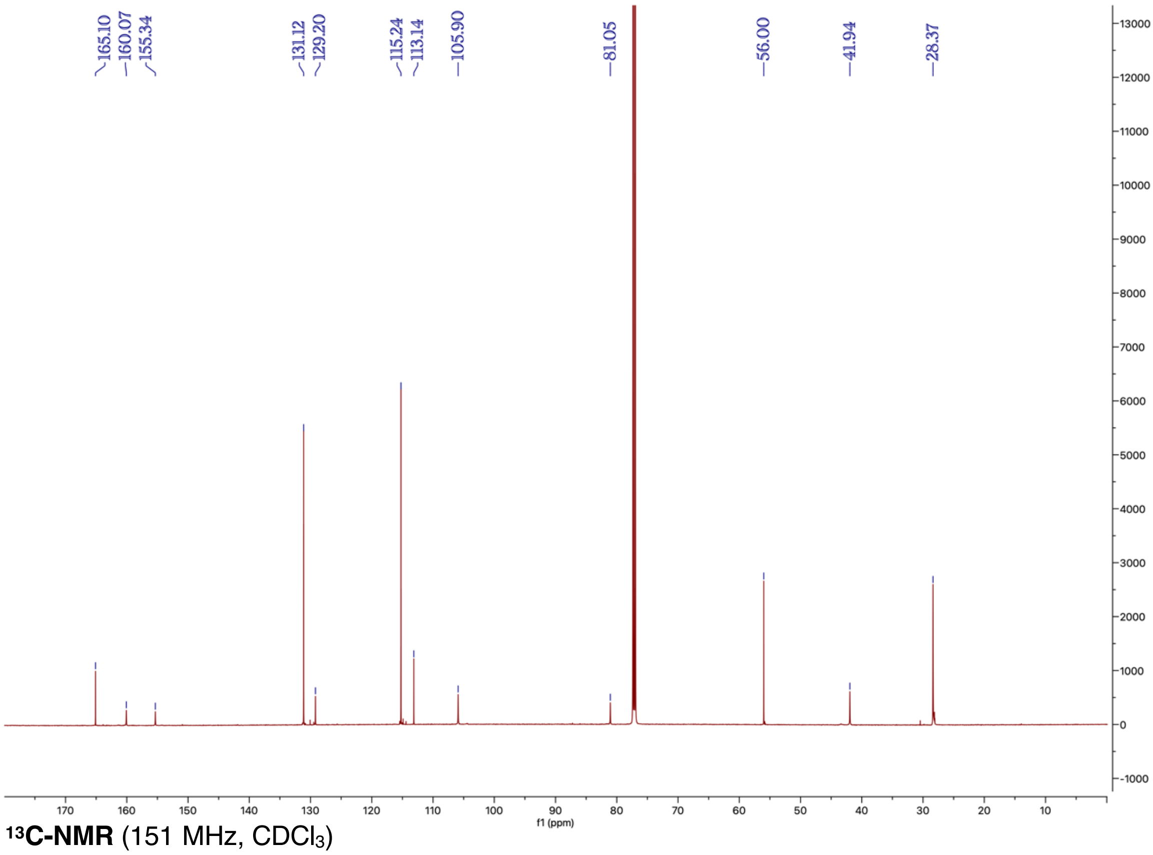

^{13}**C-NMR** (151 MHz, $CDCl_3$)

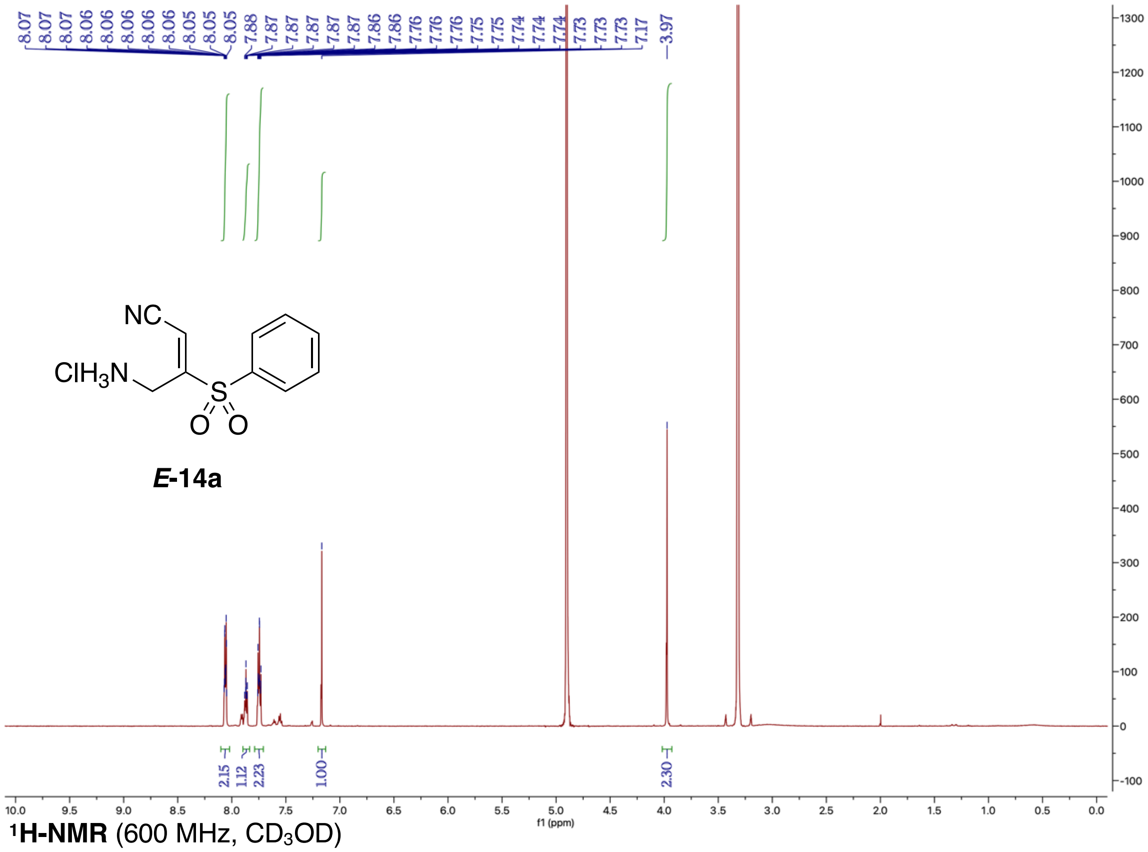

^{1}H-NMR (600 MHz, CD_3OD)

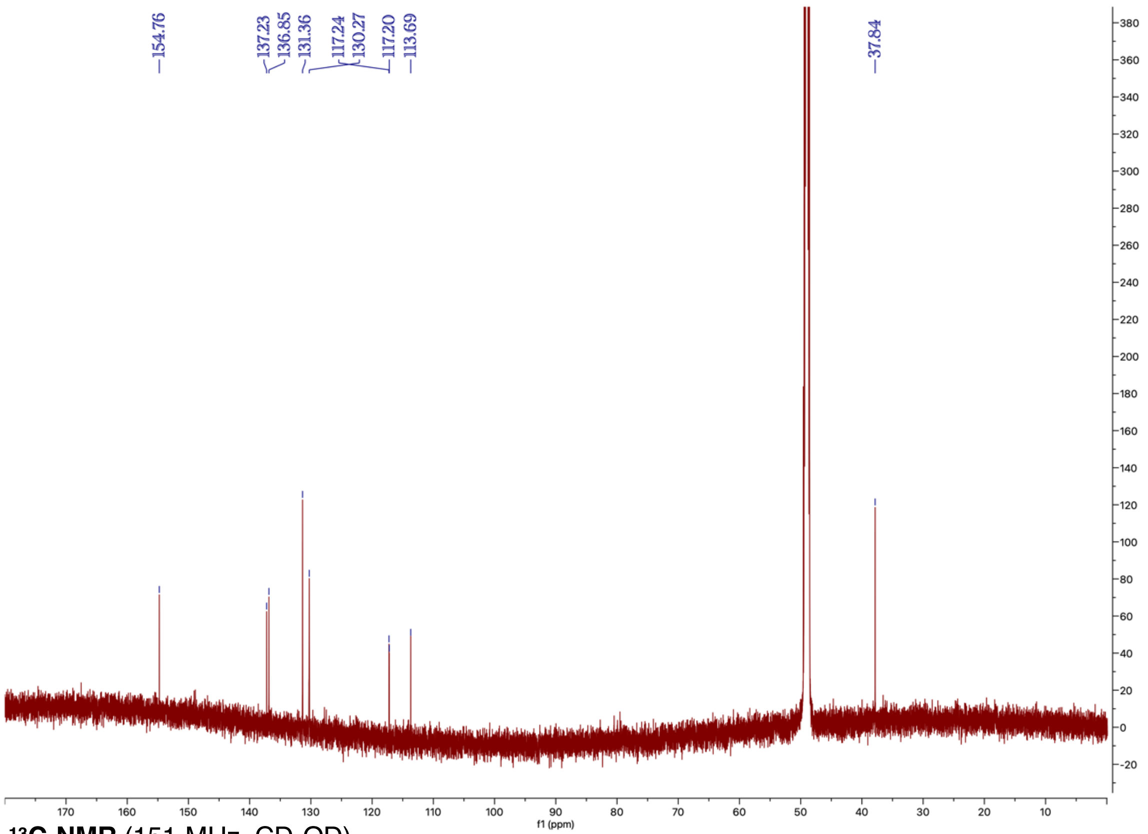

^{13}C-NMR (151 MHz, CD_3OD)

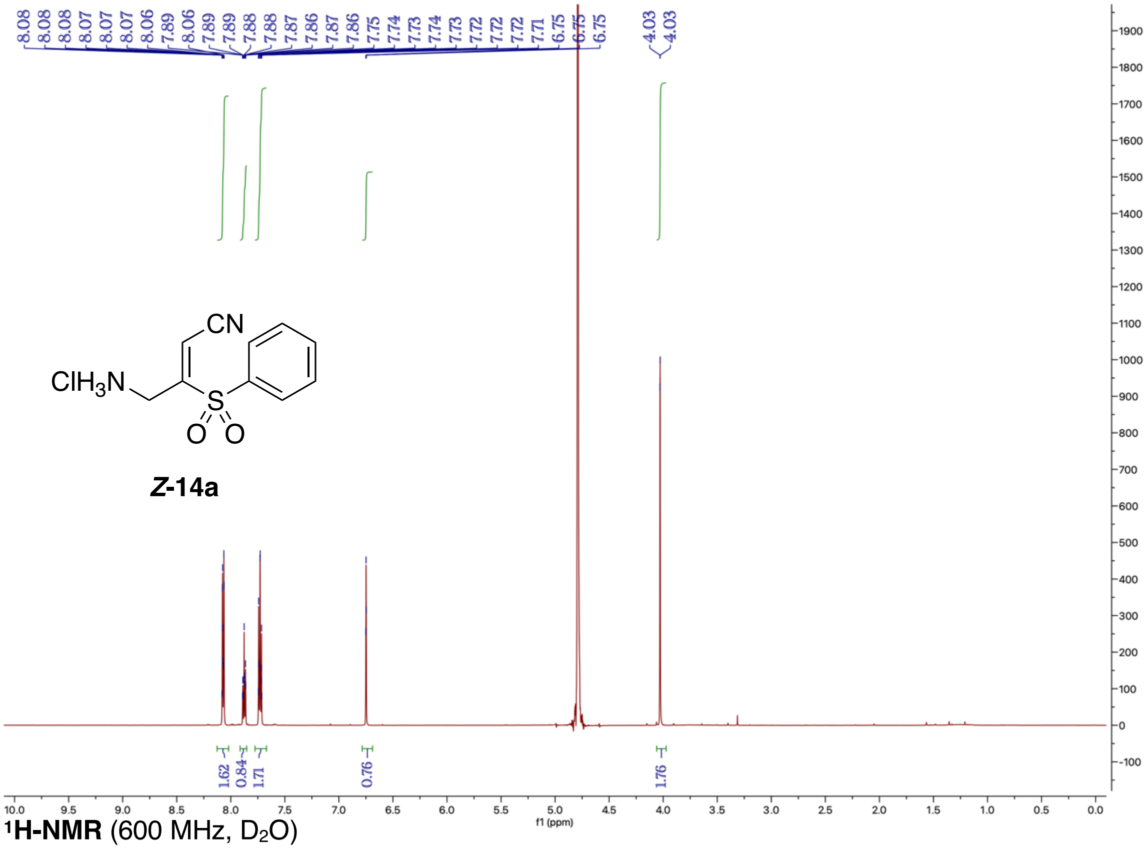

^{1}H-NMR (600 MHz, D_2O)

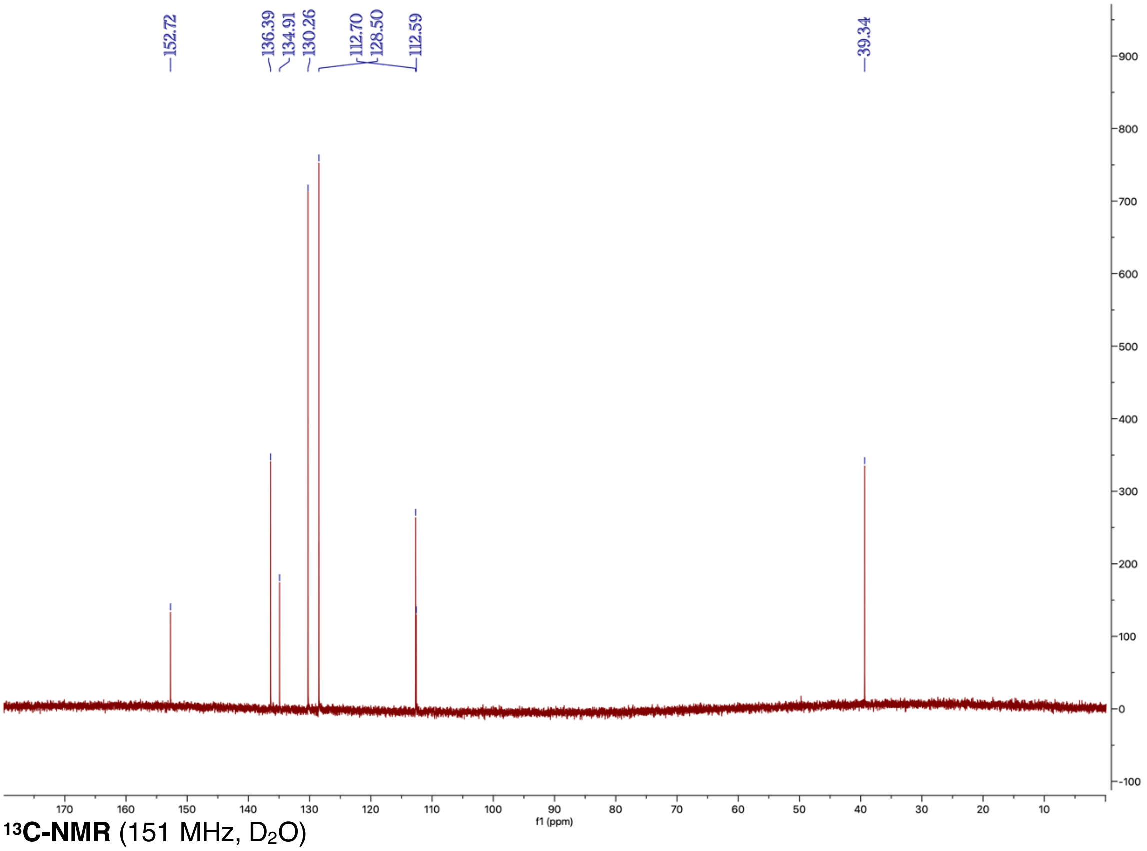

^{13}C-NMR (151 MHz, D_2O)

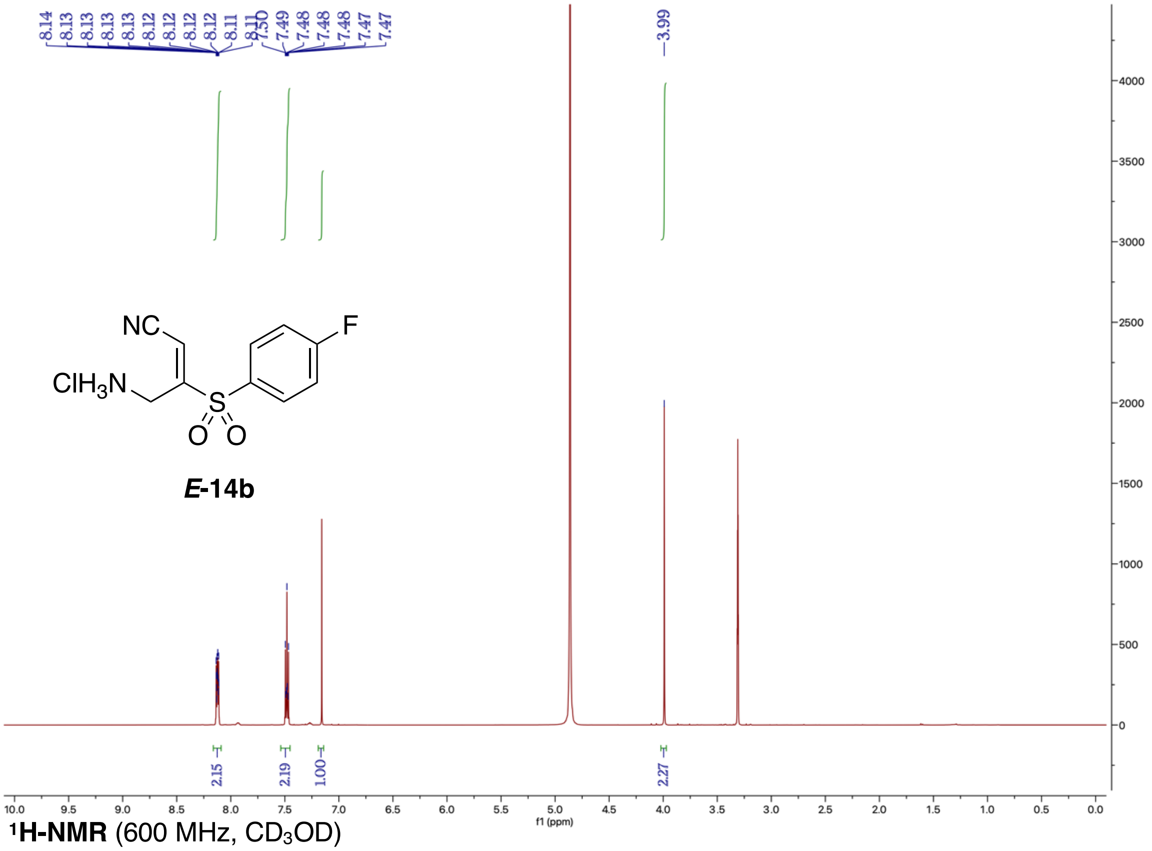

¹H-NMR (600 MHz, CD_3OD)

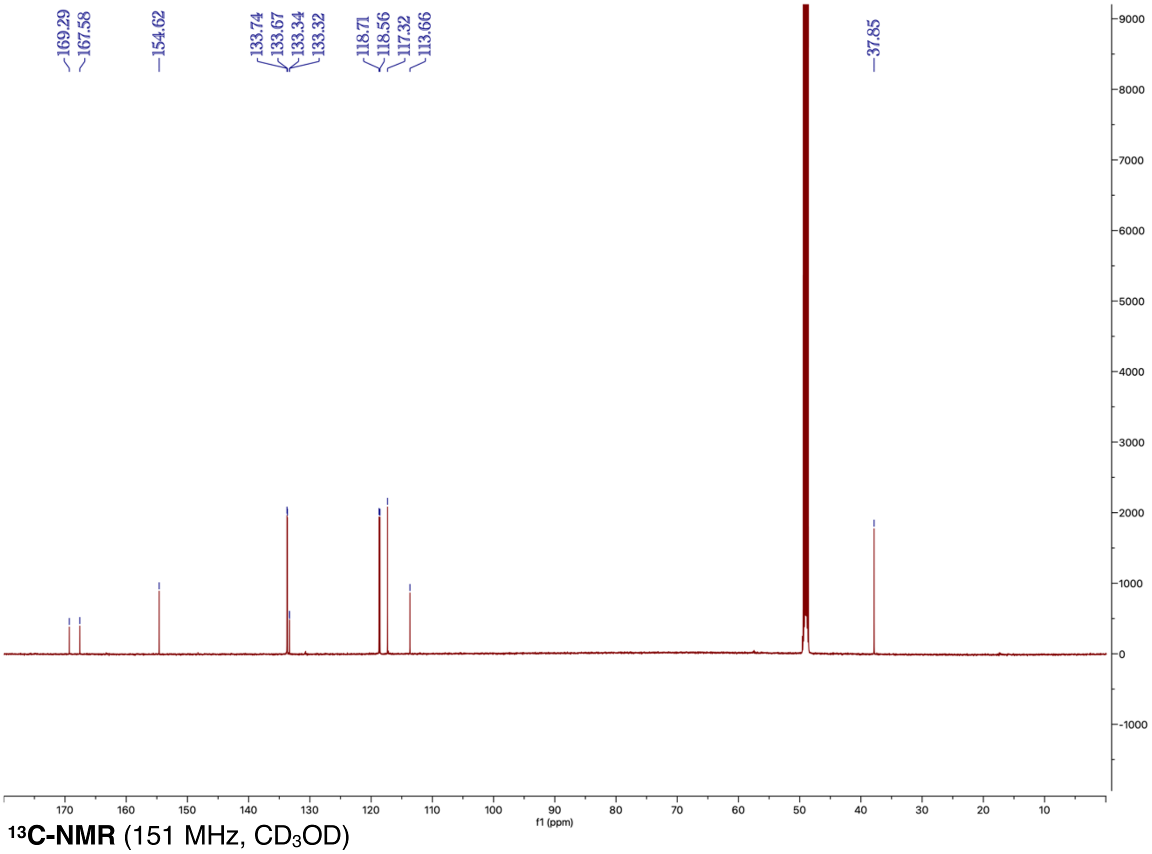

¹³C-NMR (151 MHz, CD_3OD)

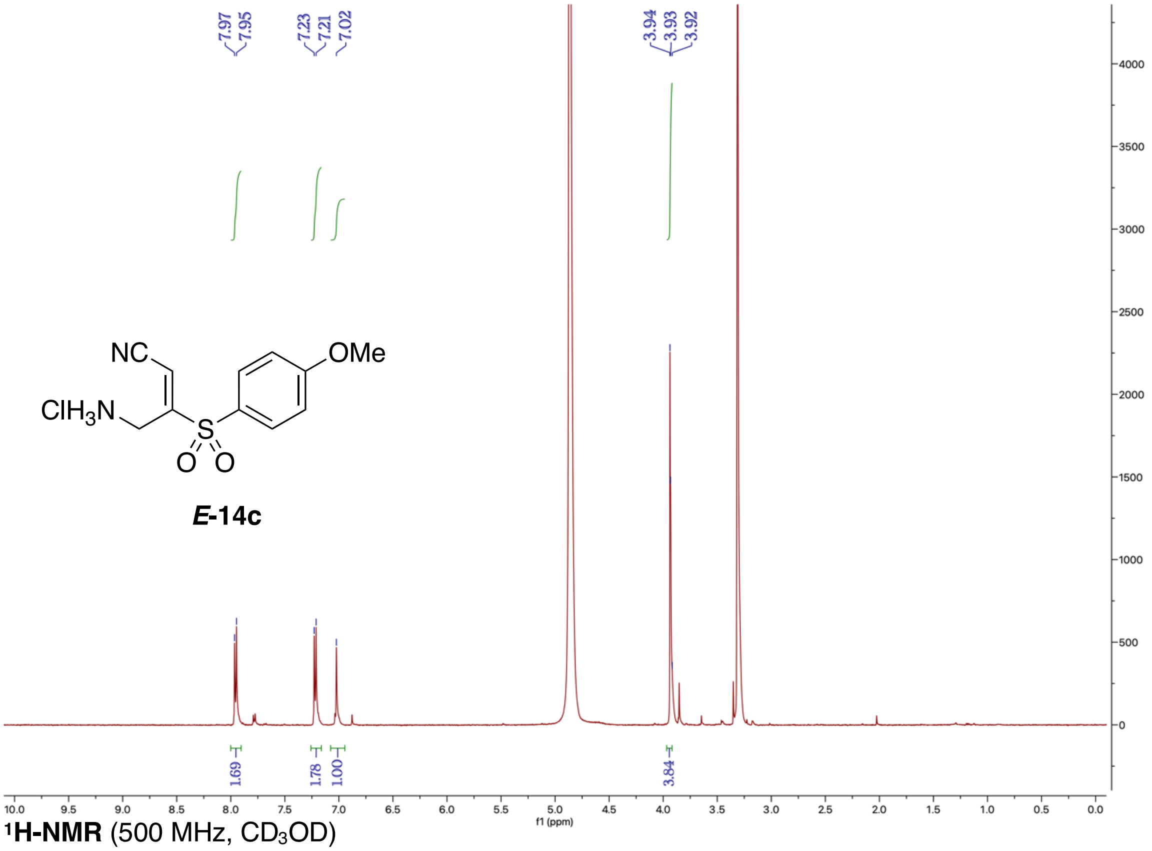

***E*-14c**

^{1}H-NMR (500 MHz, CD_3OD)

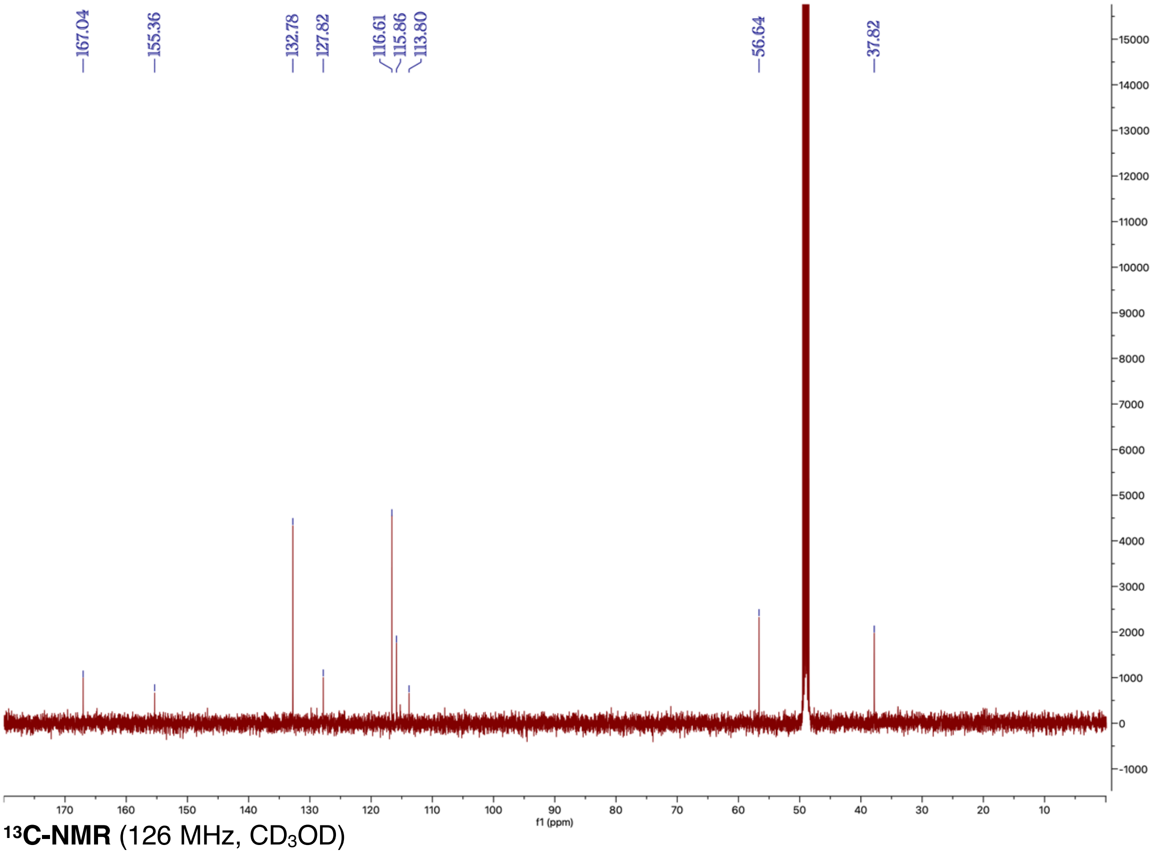

^{13}C-NMR (126 MHz, CD_3OD)

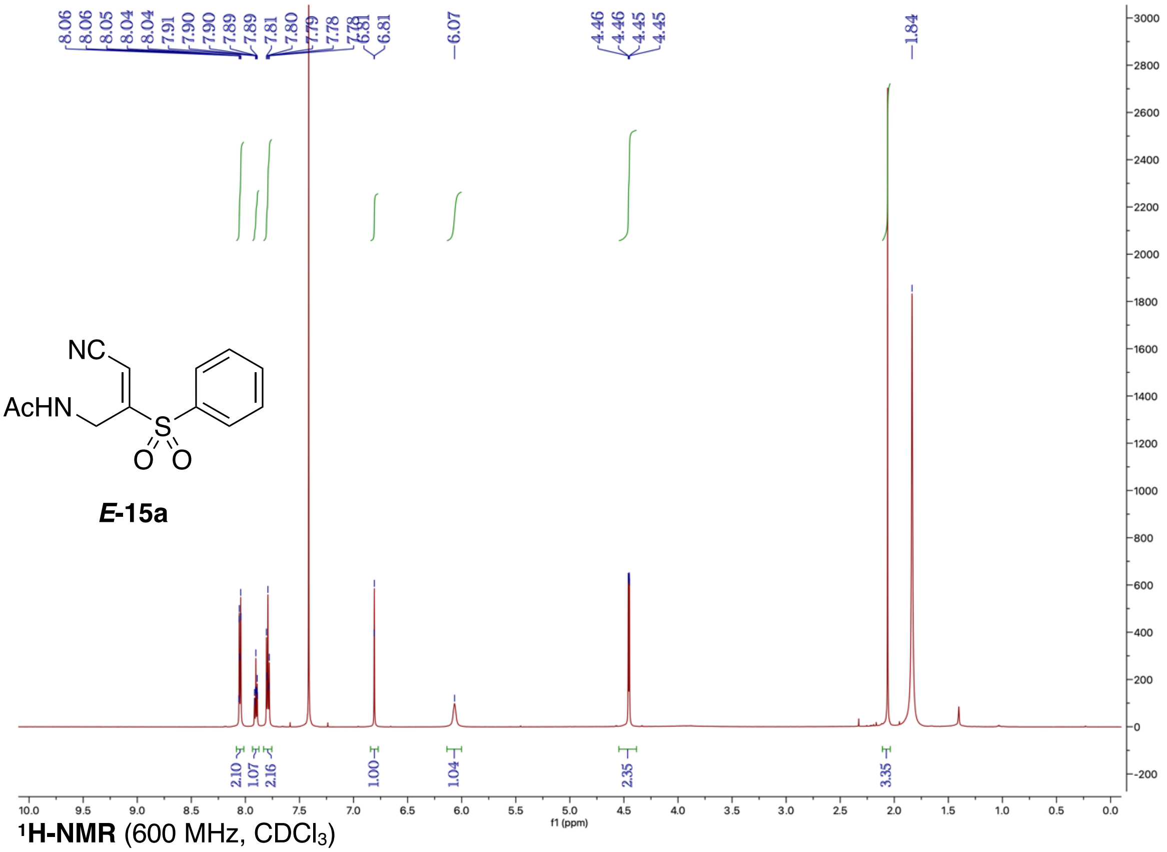

¹H-NMR (600 MHz, $CDCl_3$)

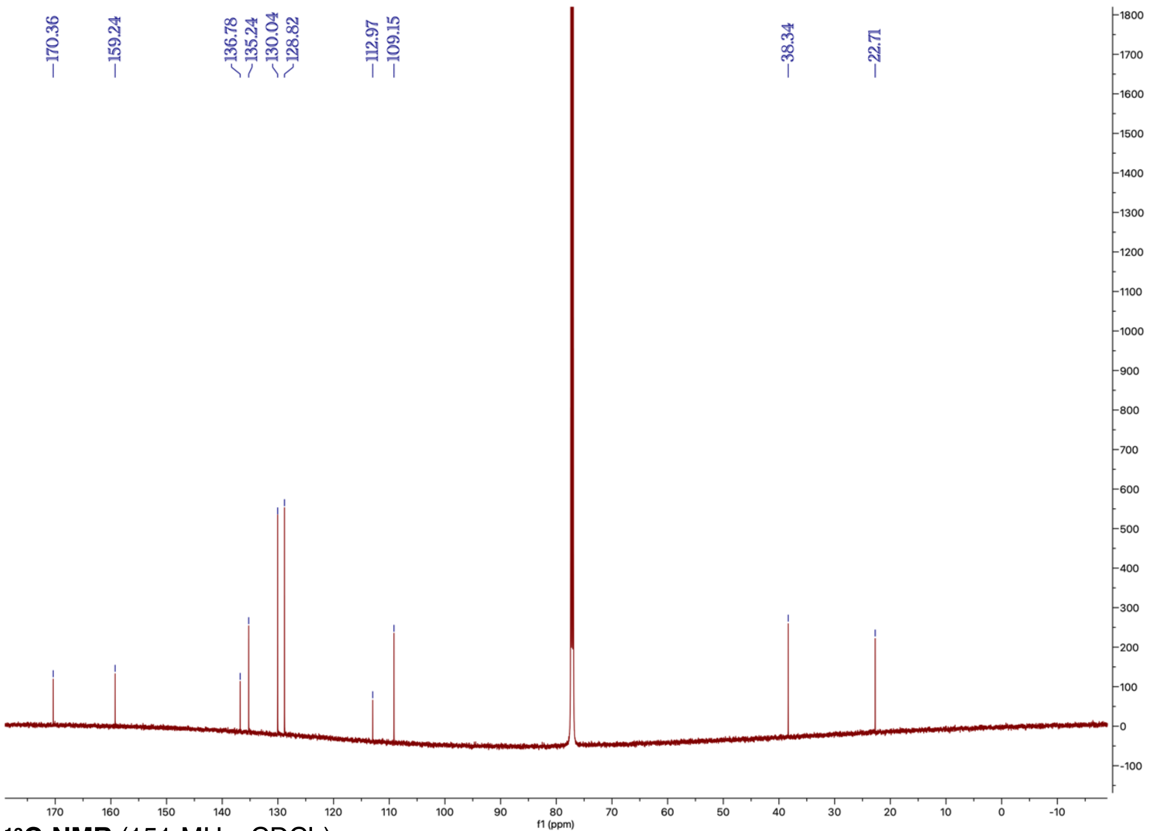

¹³C-NMR (151 MHz, $CDCl_3$)

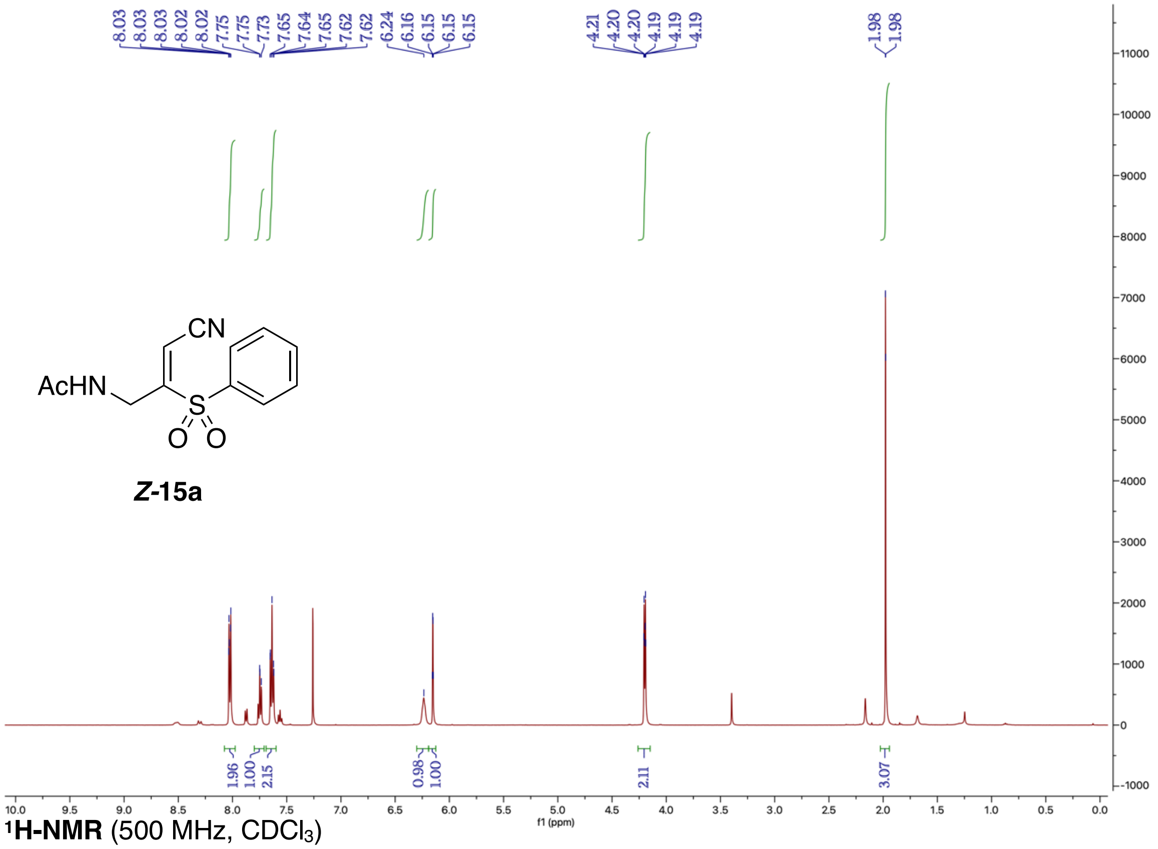

^{1}**H-NMR** (500 MHz, $CDCl_3$)

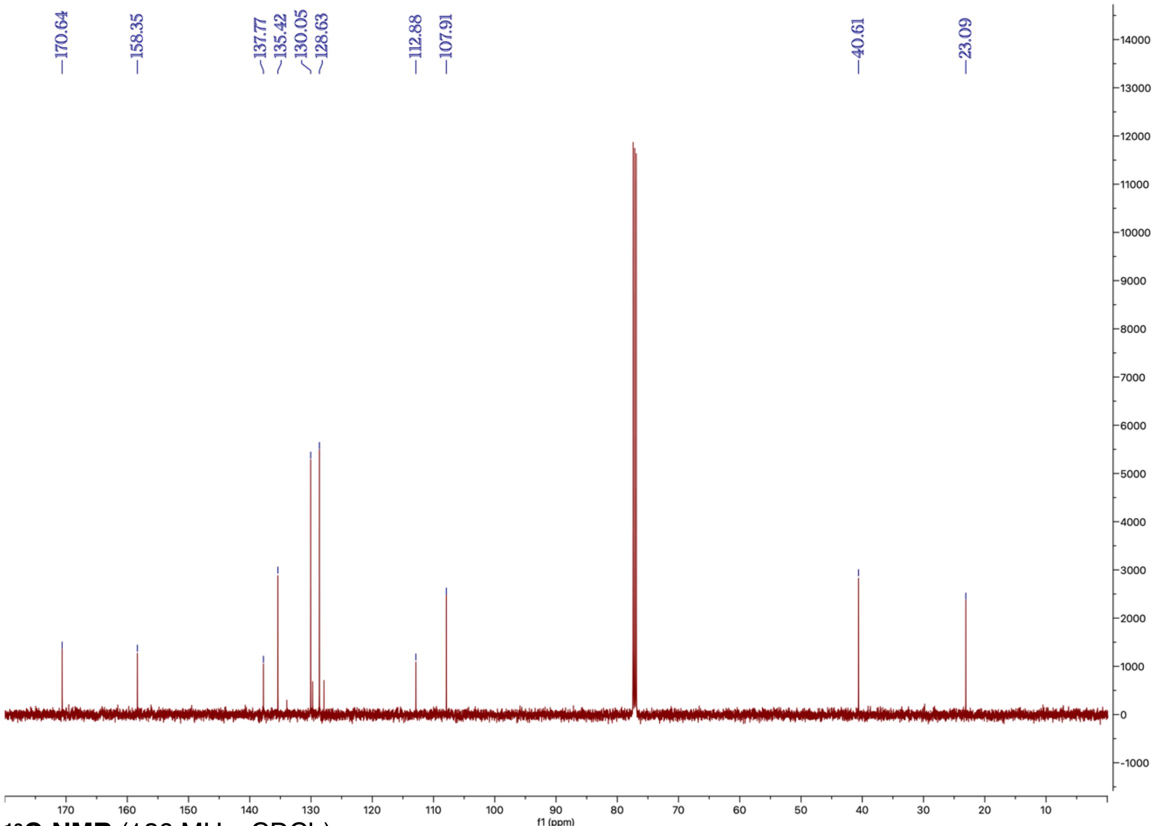

^{13}**C-NMR** (126 MHz, $CDCl_3$)

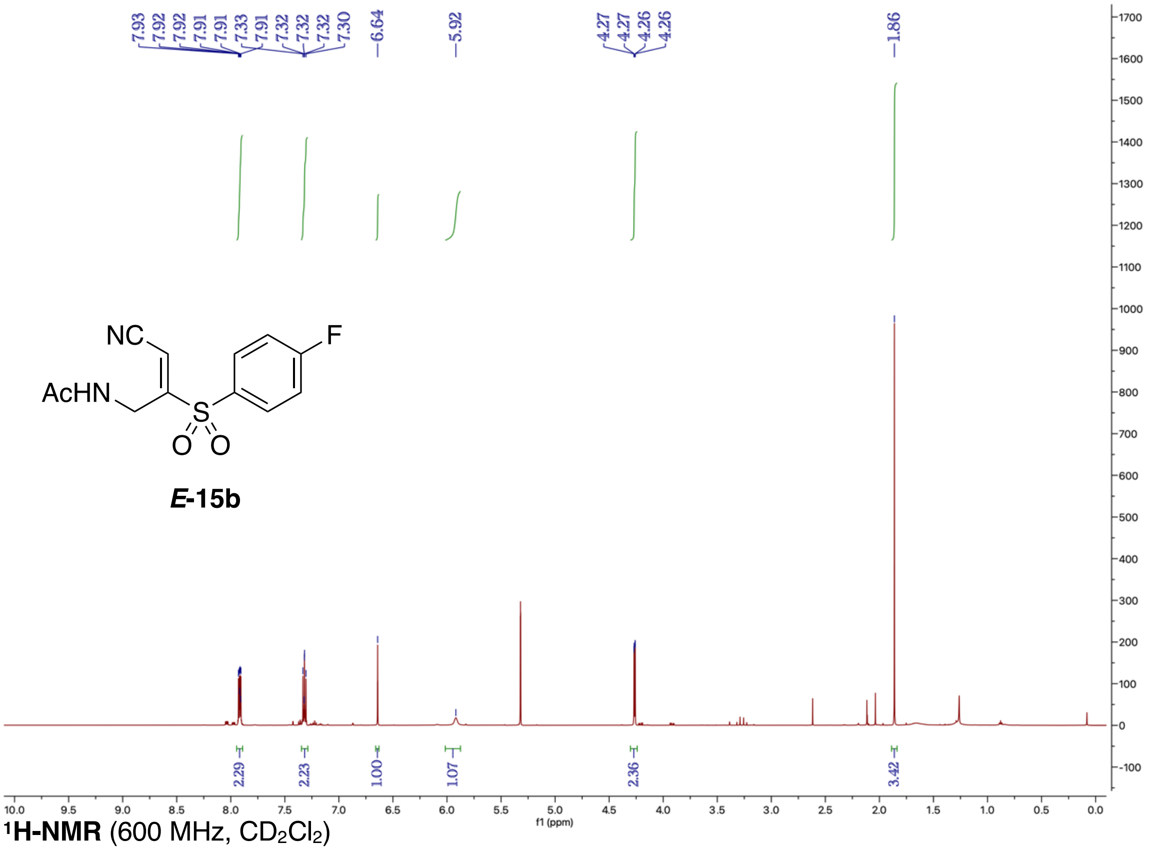

^{1}**H-NMR** (600 MHz, CD_2Cl_2)

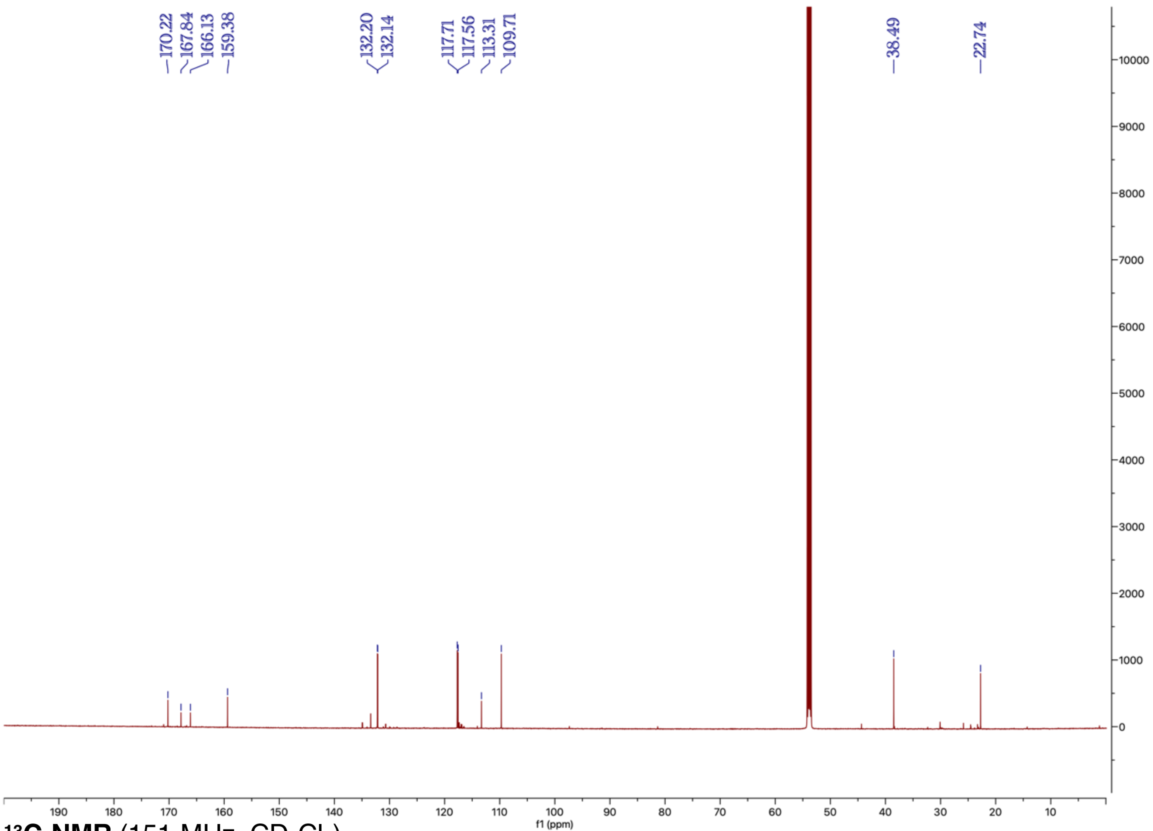

^{13}**C-NMR** (151 MHz, CD_2Cl_2)

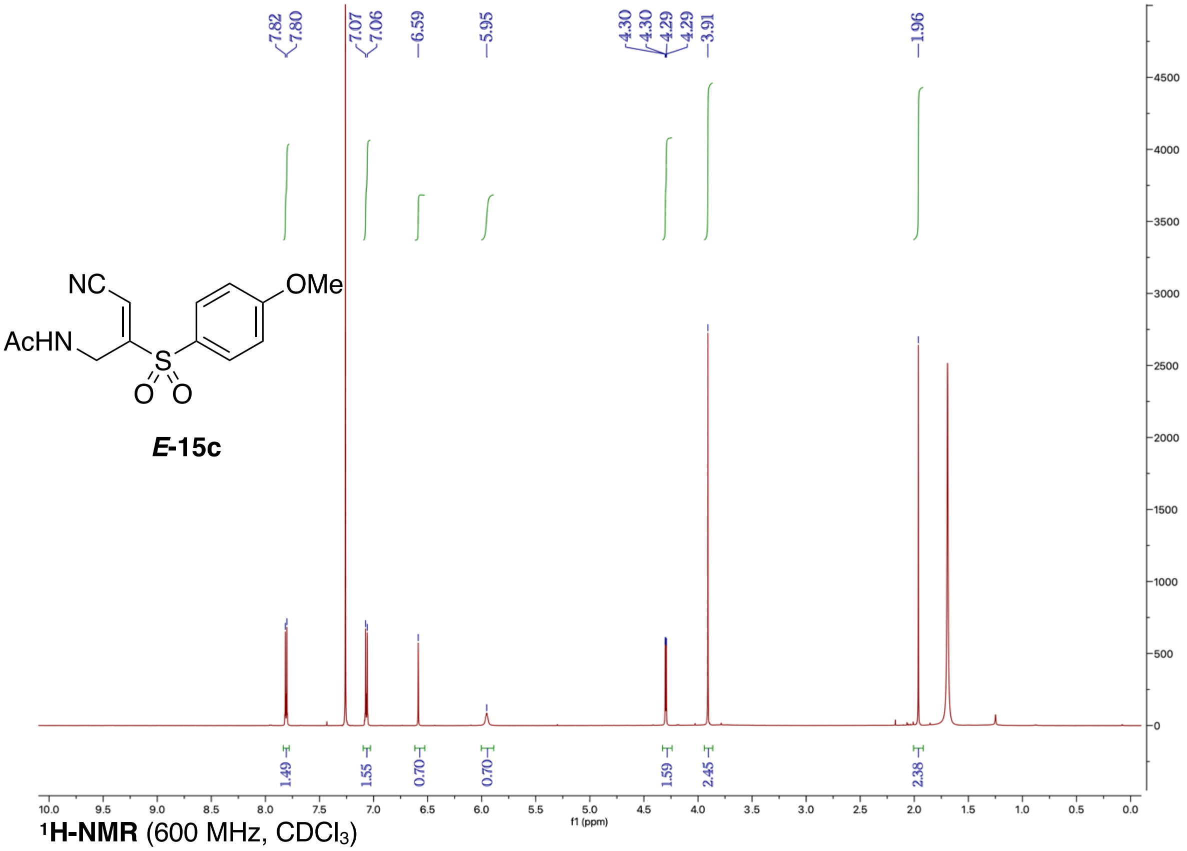

¹H-NMR (600 MHz, $CDCl_3$)

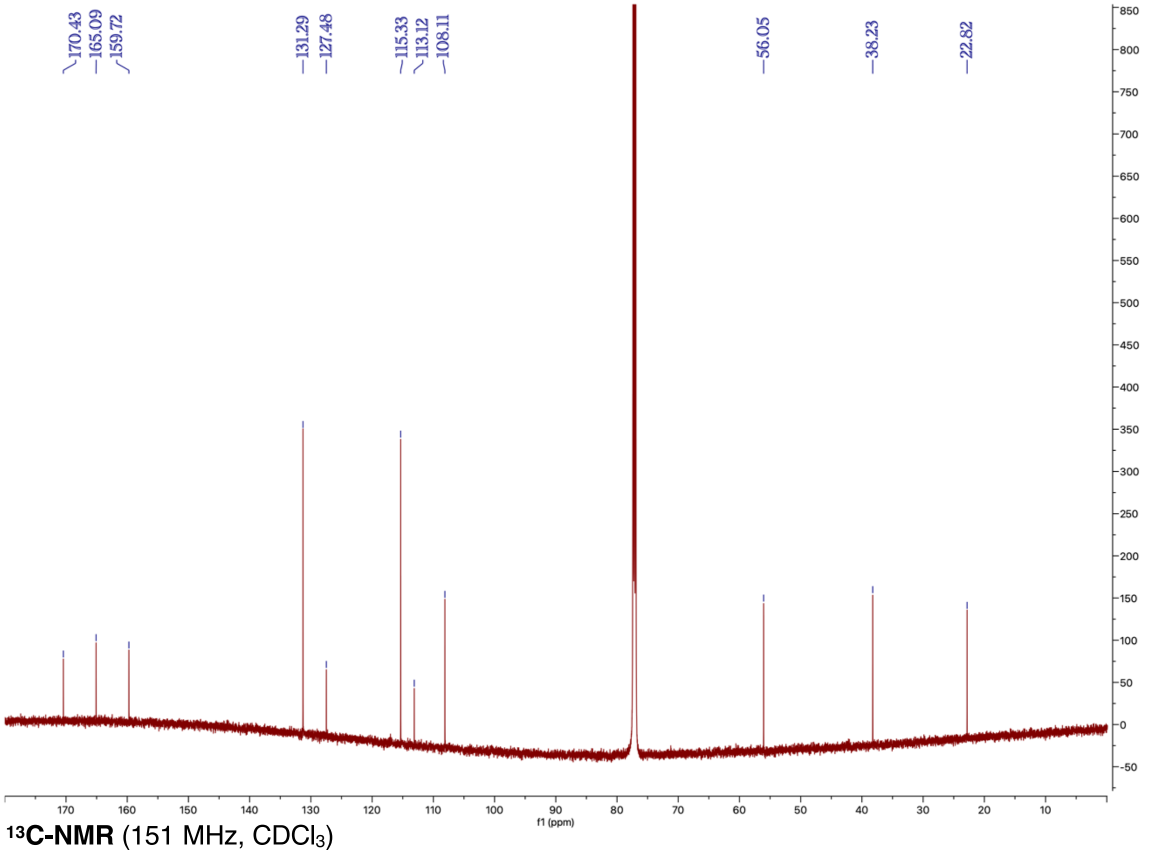

¹³C-NMR (151 MHz, $CDCl_3$)

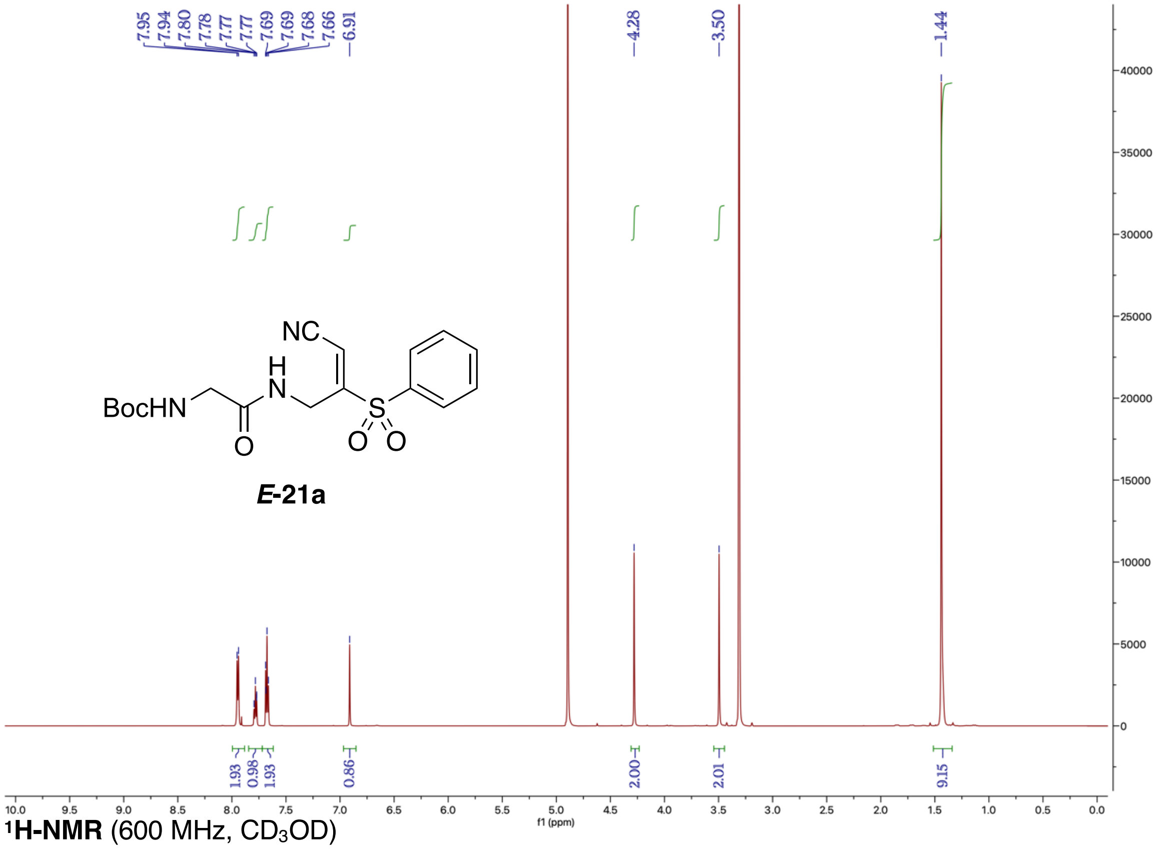

^{1}H-NMR (600 MHz, CD_3OD)

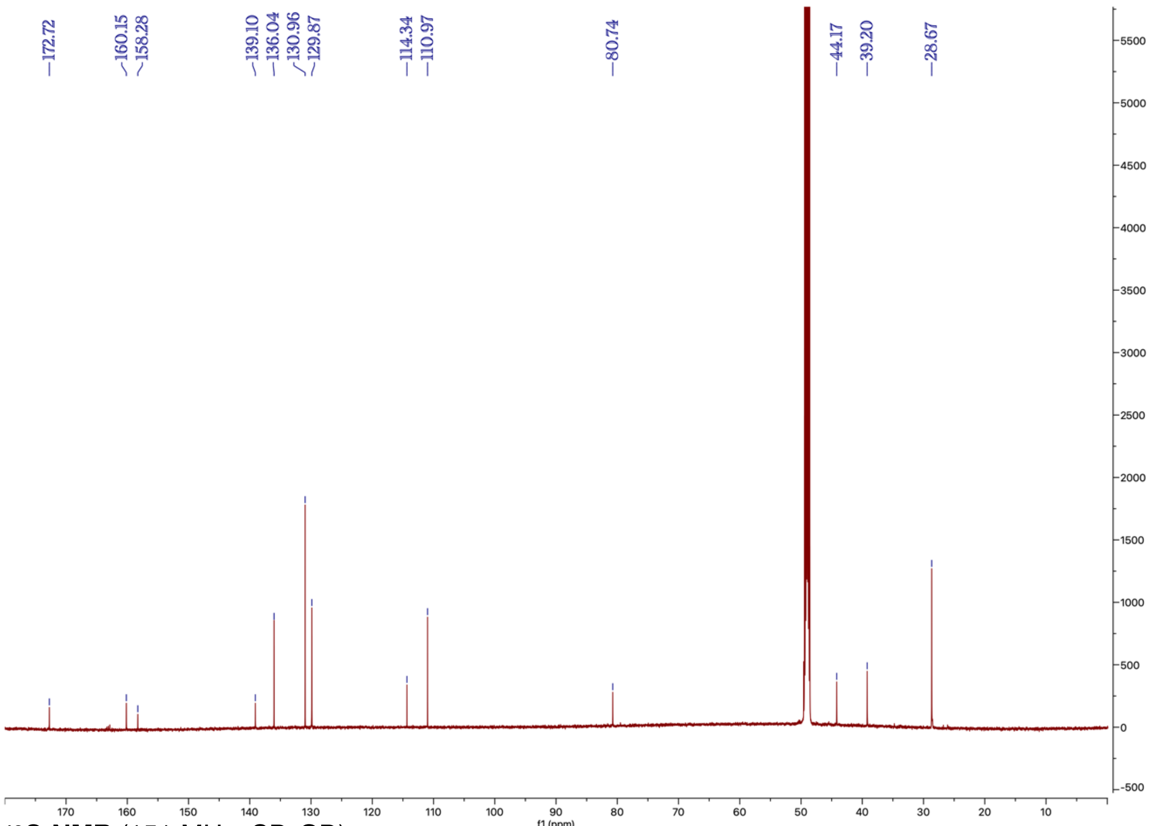

^{13}C-NMR (151 MHz, CD_3OD)

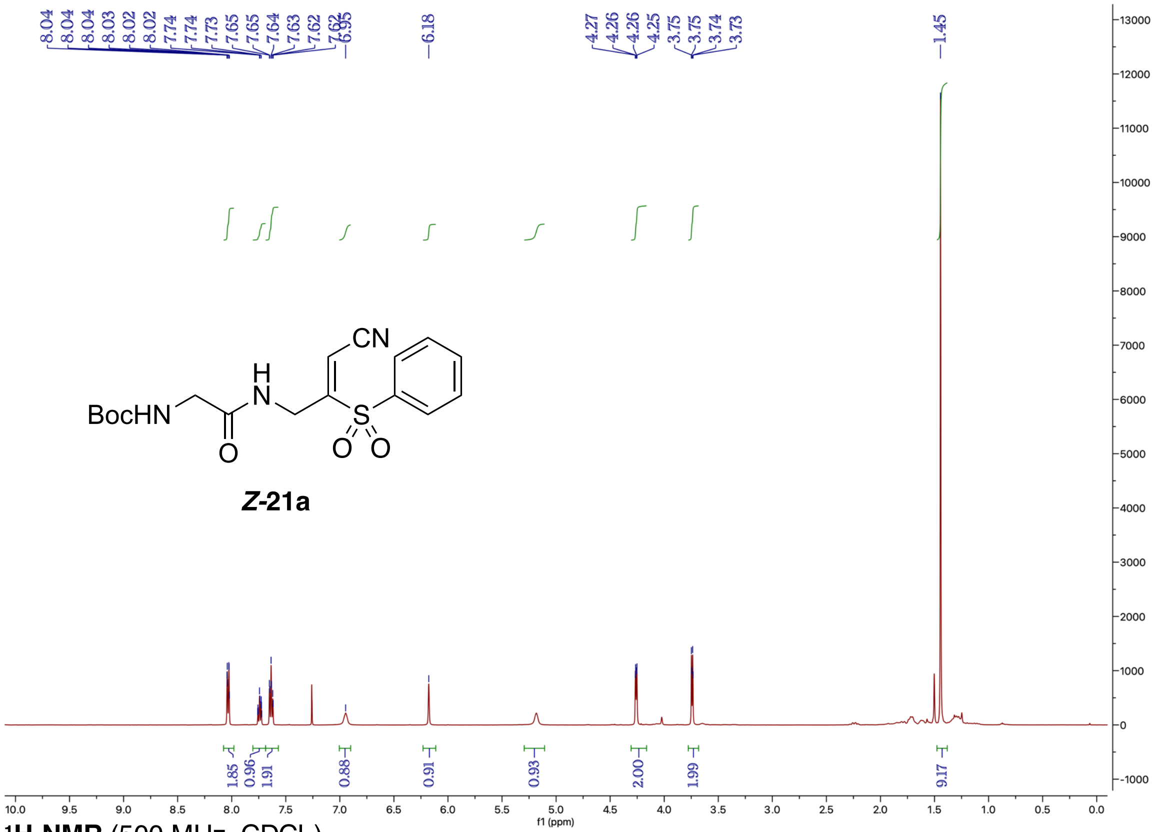

¹H-NMR (500 MHz, $CDCl_3$)

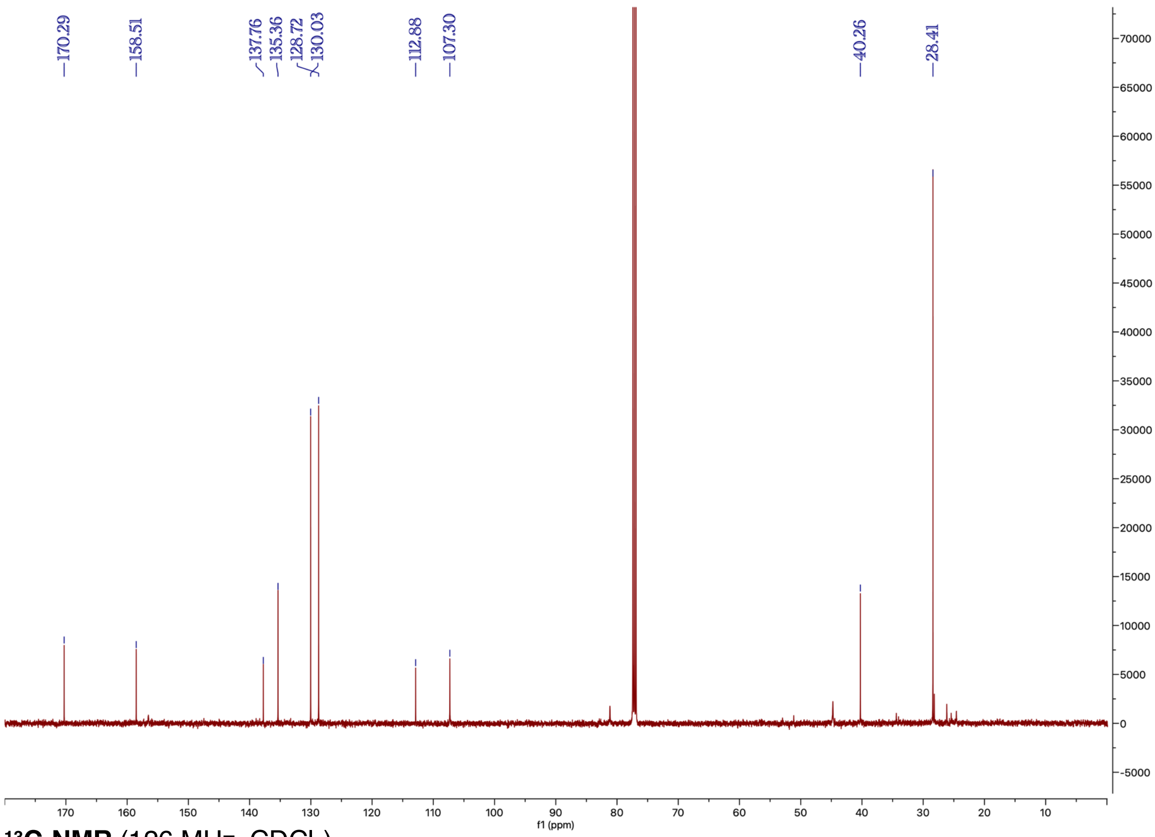

¹³C-NMR (126 MHz, $CDCl_3$)

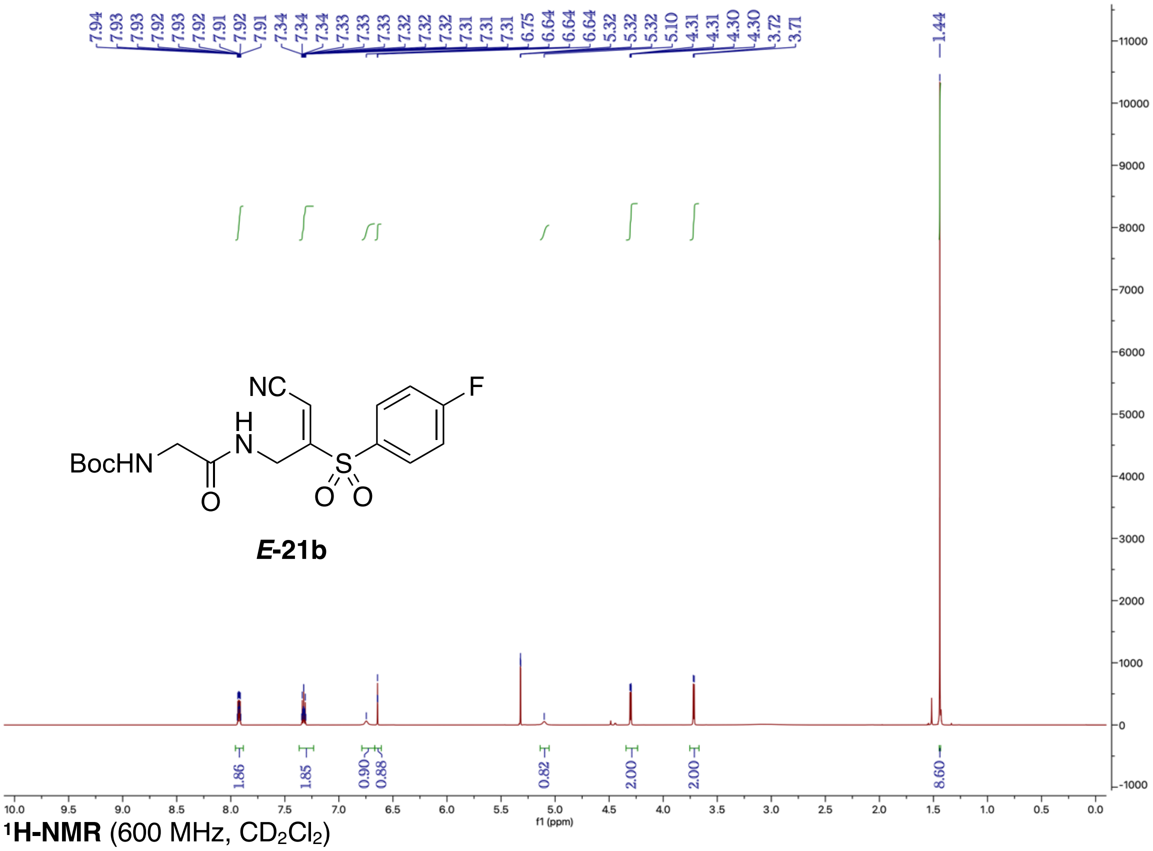

^{1}**H-NMR** (600 MHz, CD_2Cl_2)

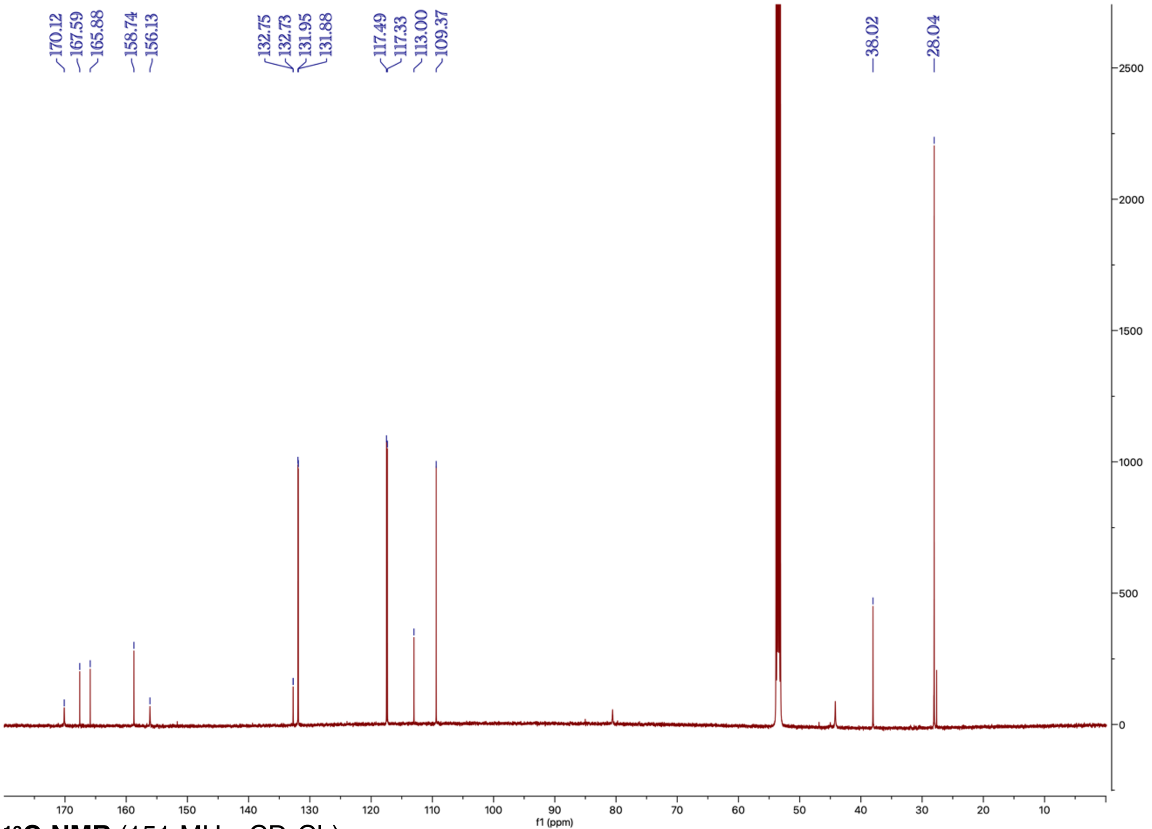

^{13}**C-NMR** (151 MHz, CD_2Cl_2)

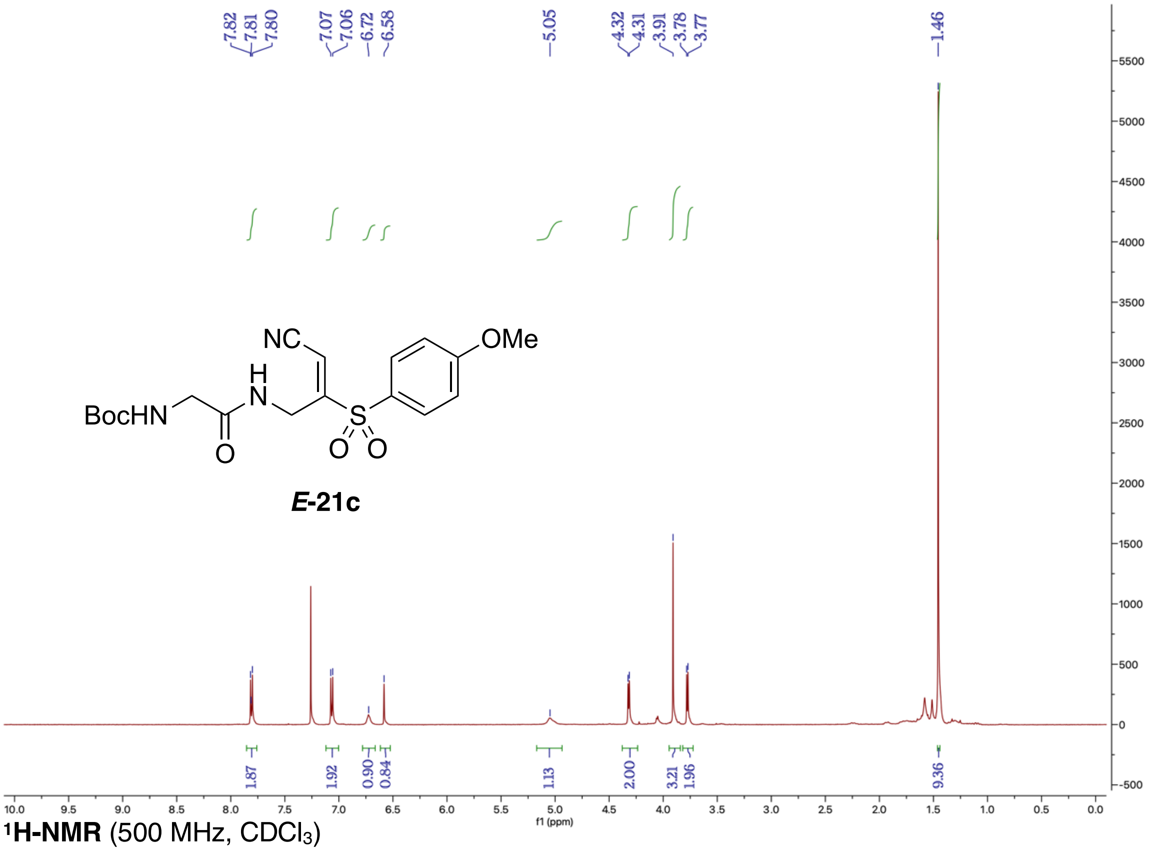

^{1}H-NMR (500 MHz, $CDCl_3$)

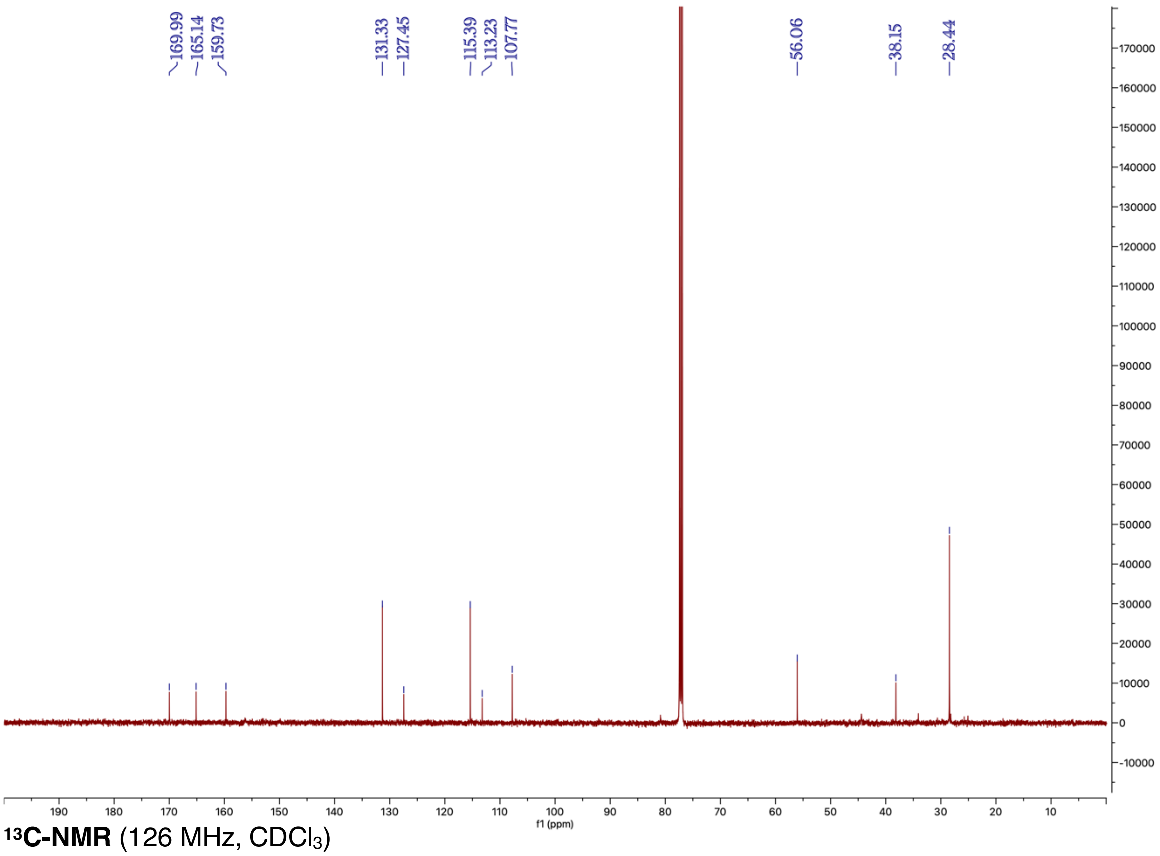

^{13}C-NMR (126 MHz, $CDCl_3$)

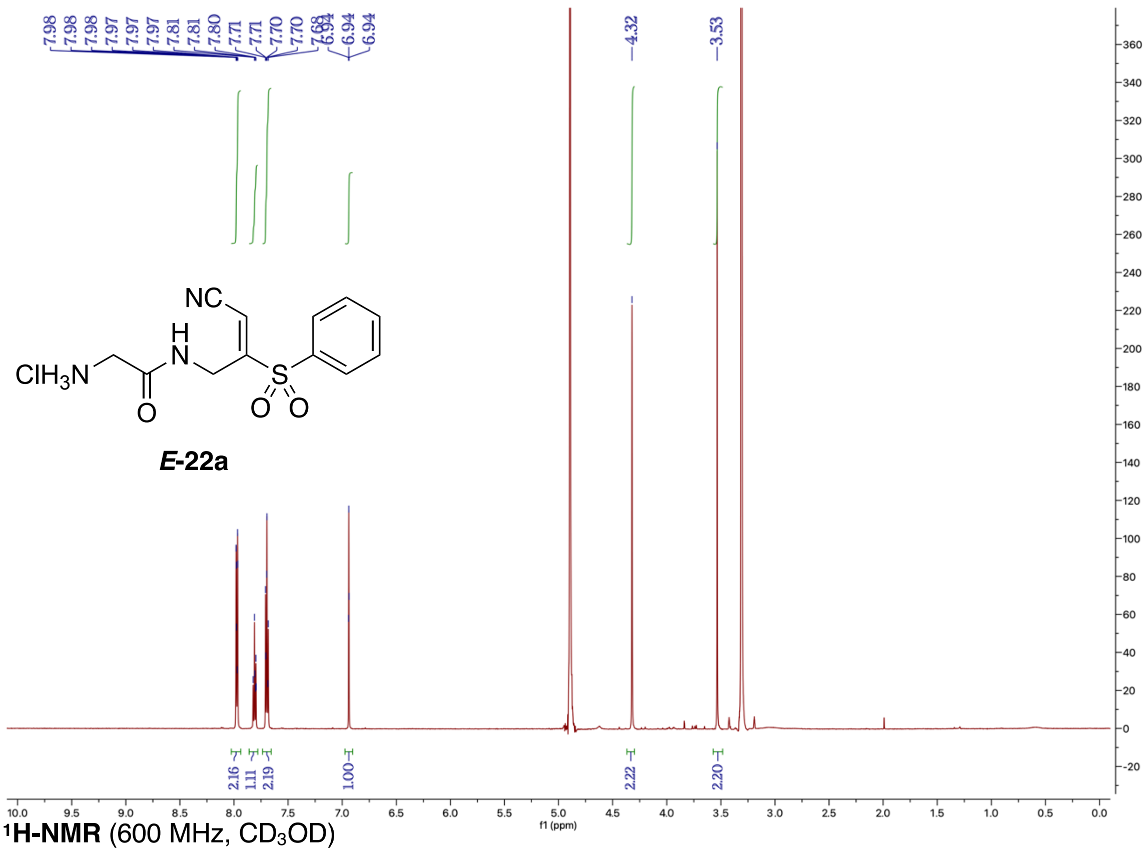

^{1}H-NMR (600 MHz, CD_3OD)

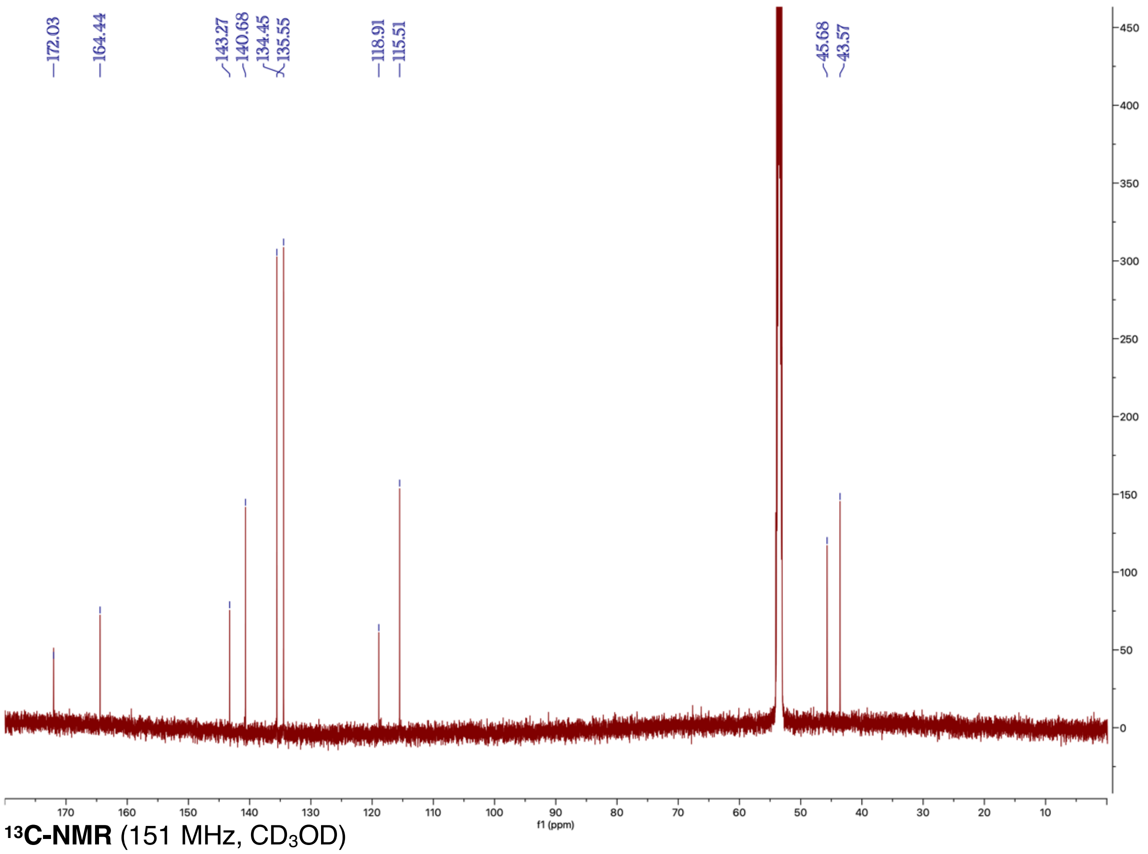

^{13}C-NMR (151 MHz, CD_3OD)

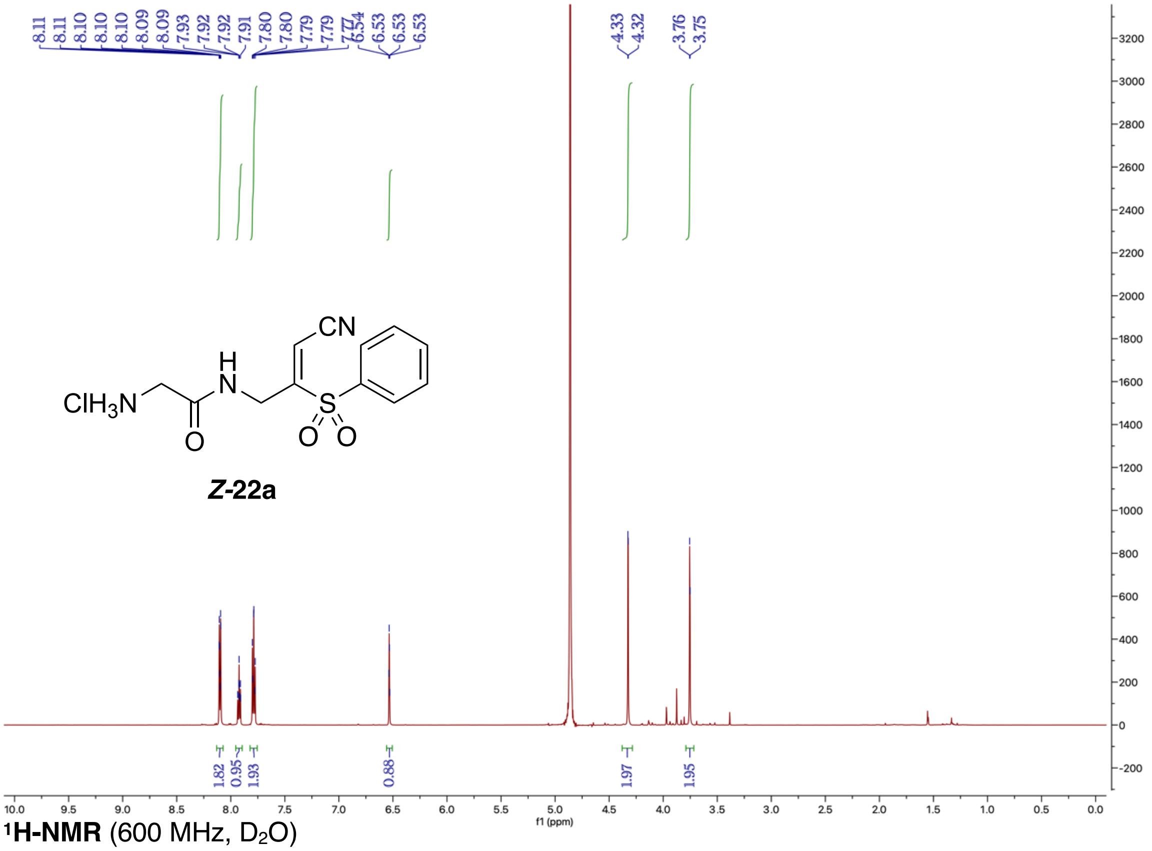

^{1}**H-NMR** (600 MHz, D_2O)

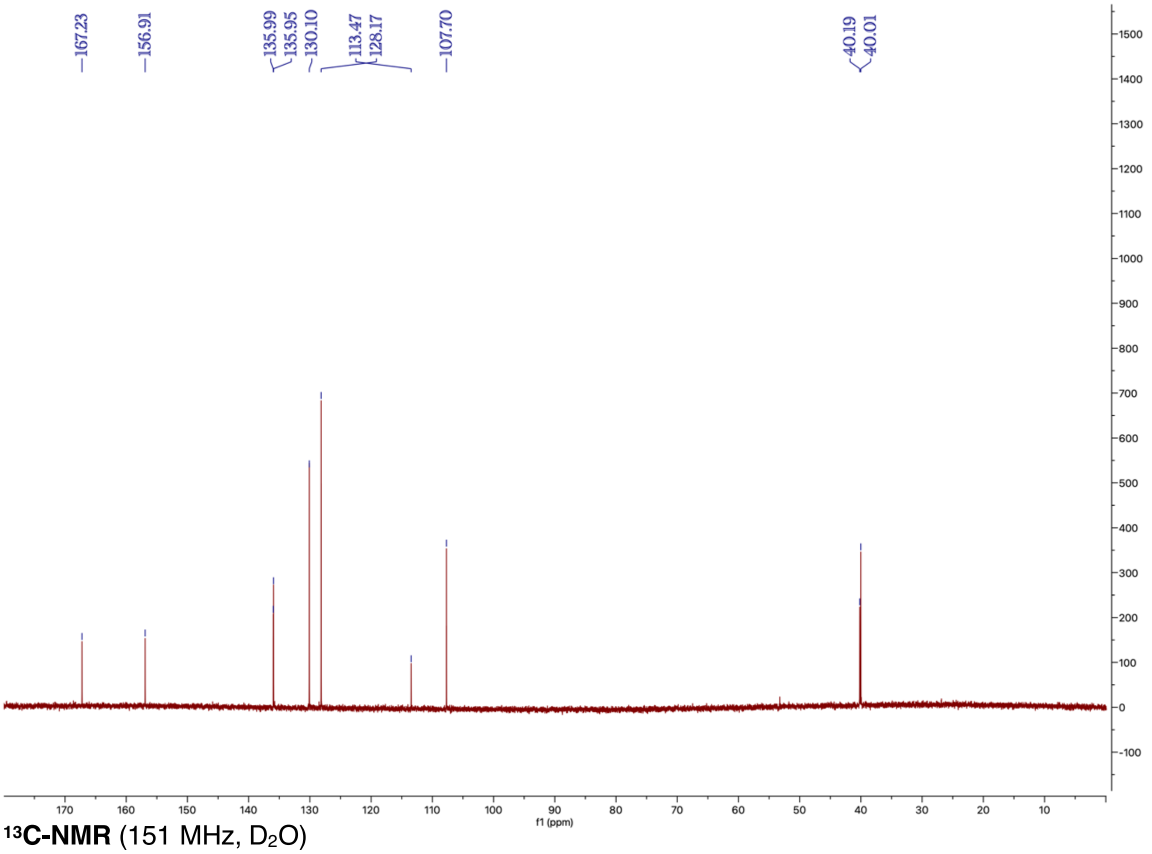

^{13}**C-NMR** (151 MHz, D_2O)

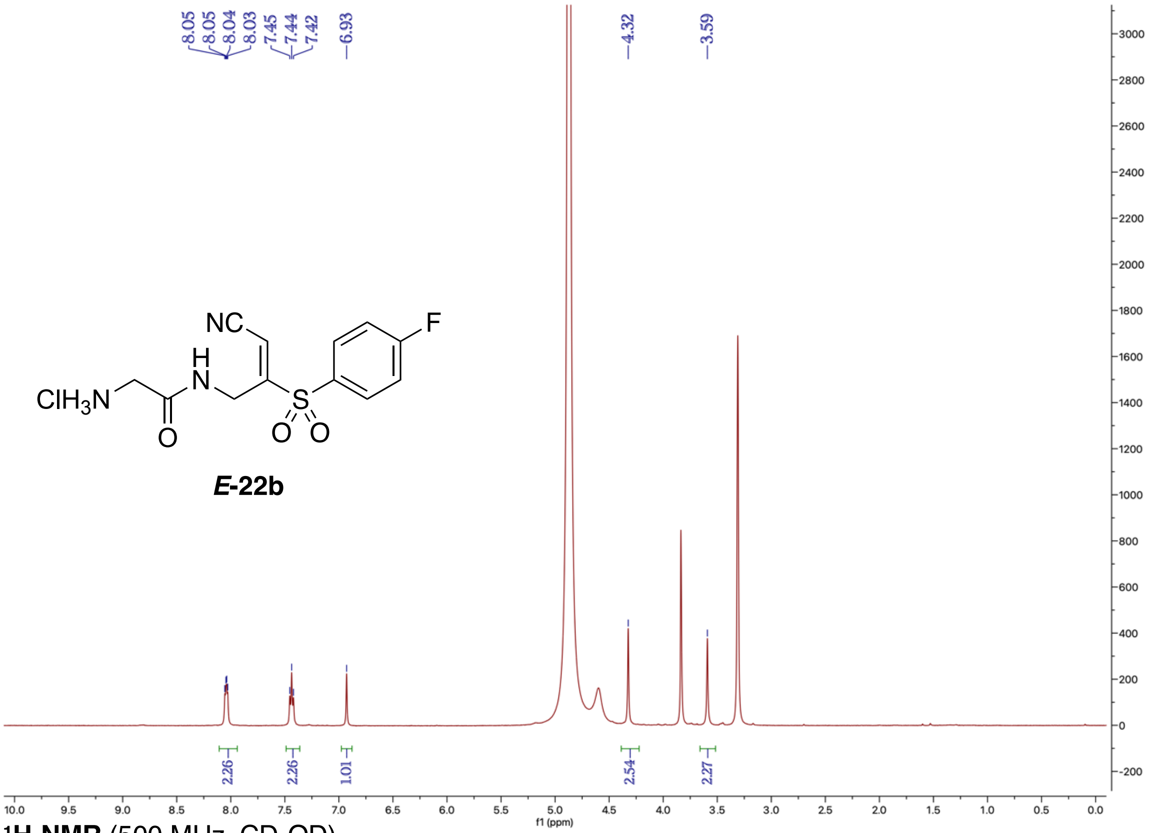

¹H-NMR (500 MHz, CD_3OD)

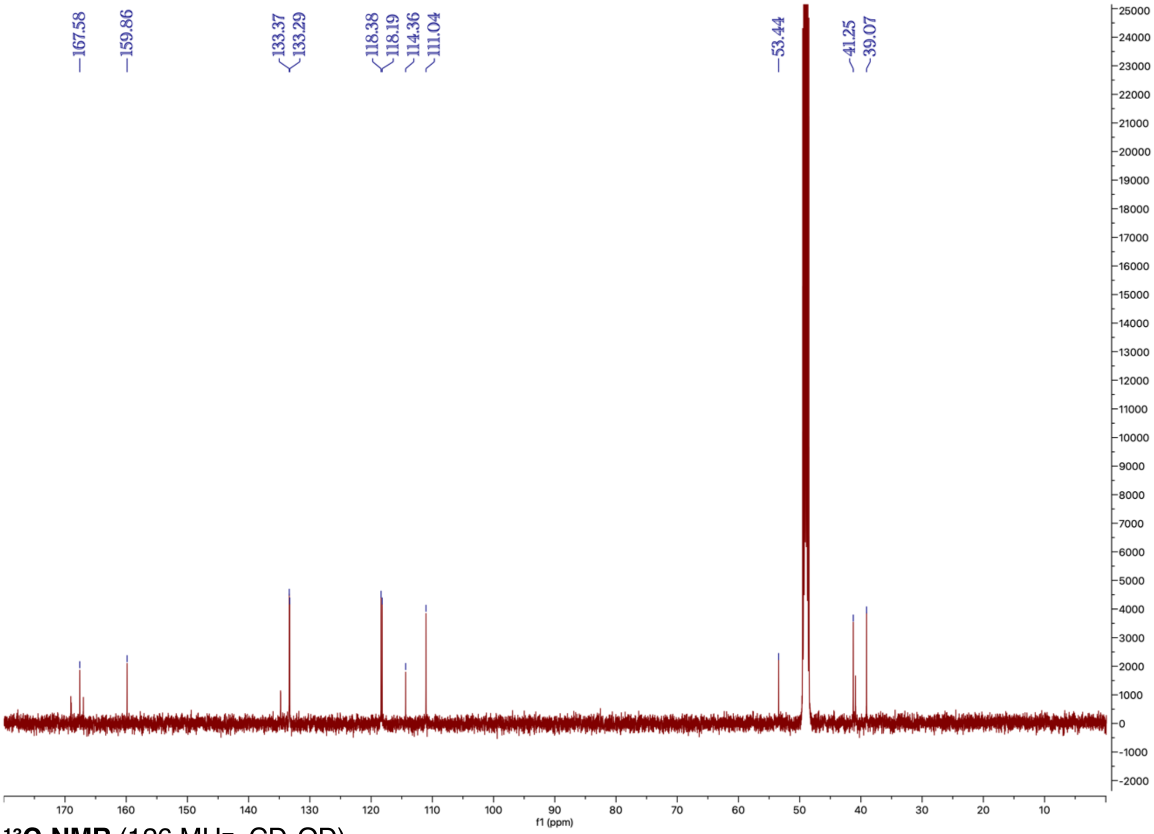

¹³C-NMR (126 MHz, CD_3OD)

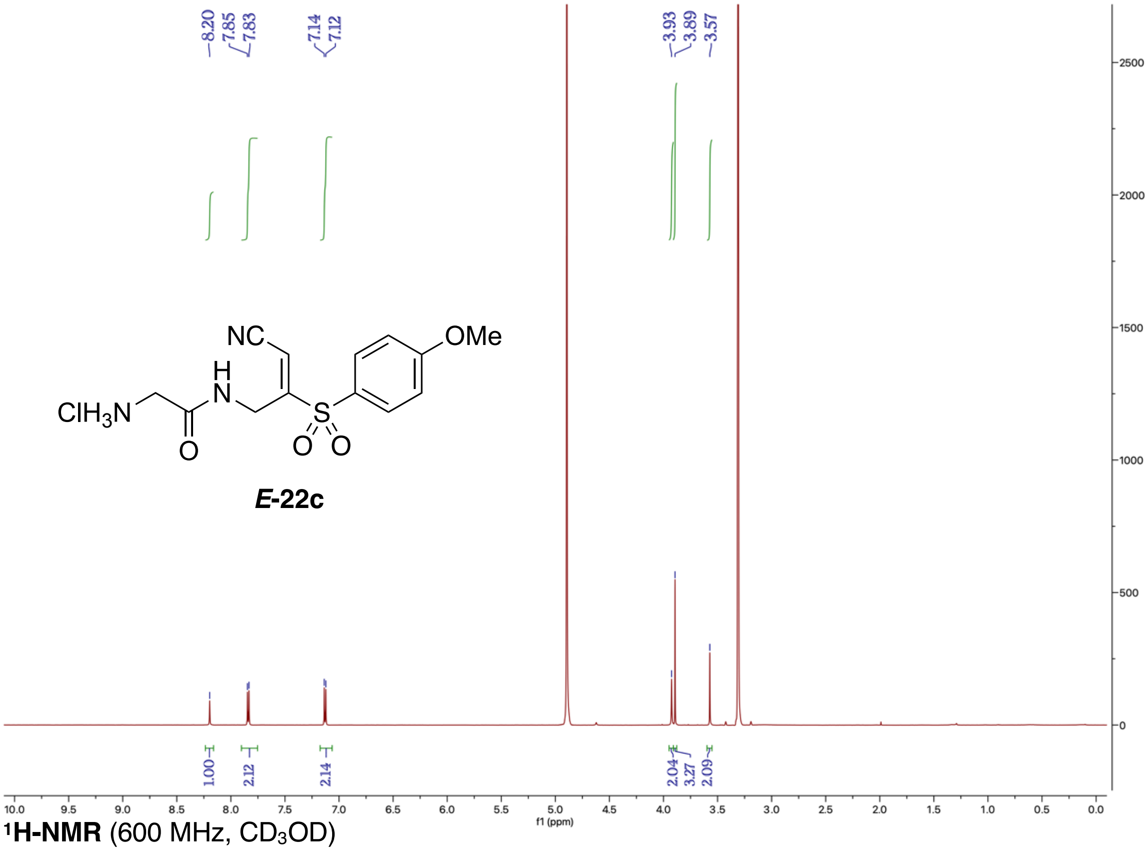

^{1}**H-NMR** (600 MHz, CD_3OD)

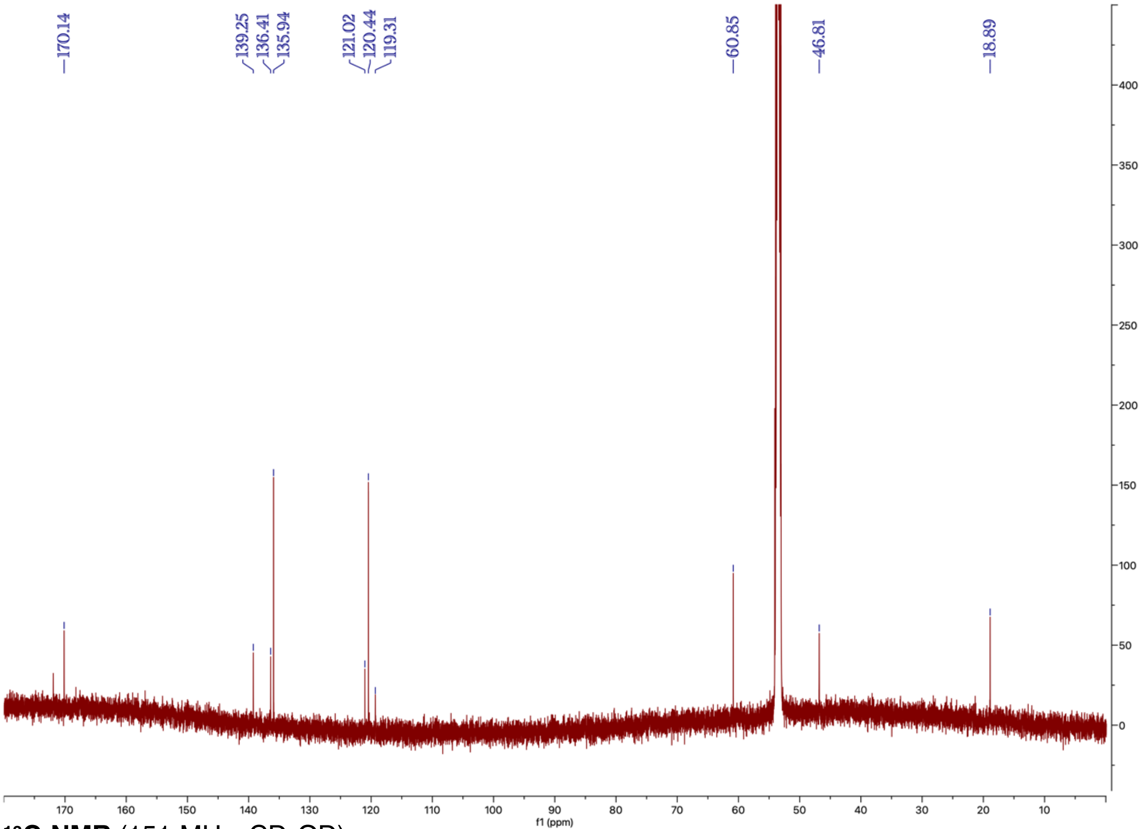

^{13}**C-NMR** (151 MHz, CD_3OD)

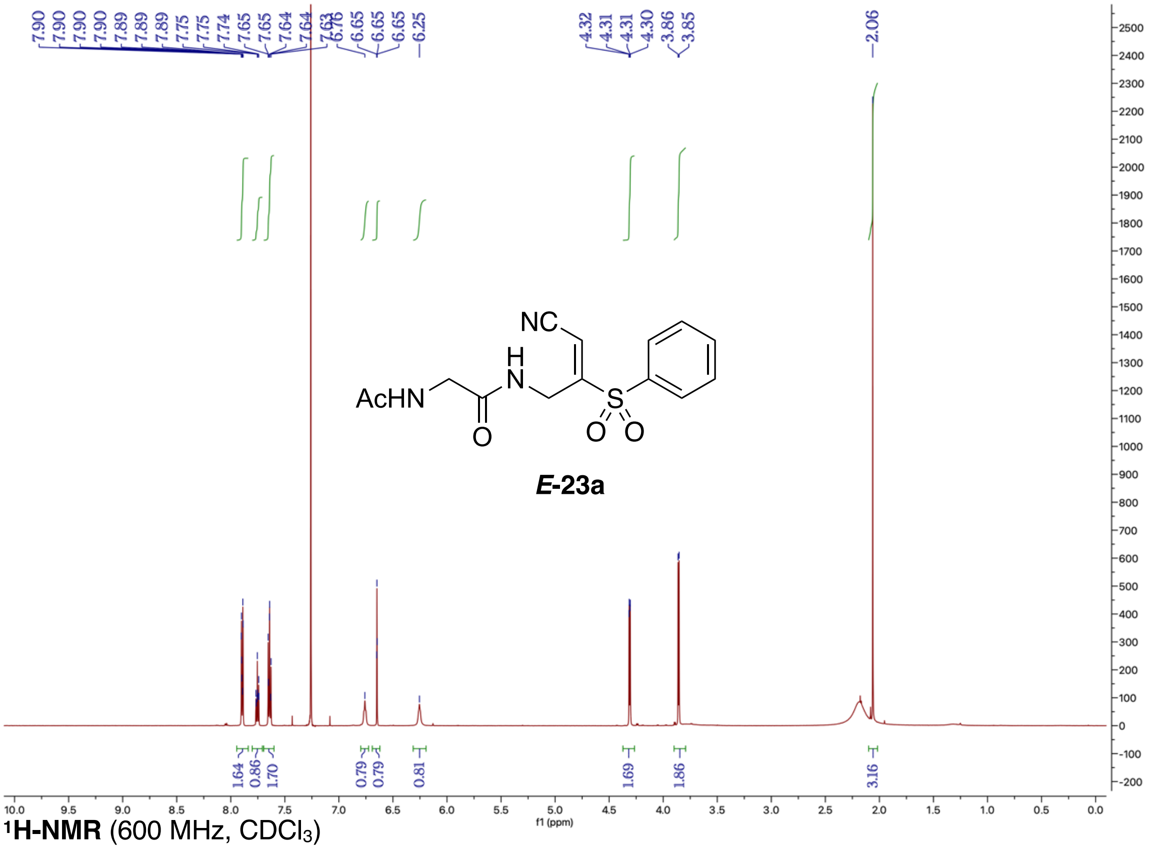

^{1}**H-NMR** (600 MHz, $CDCl_3$)

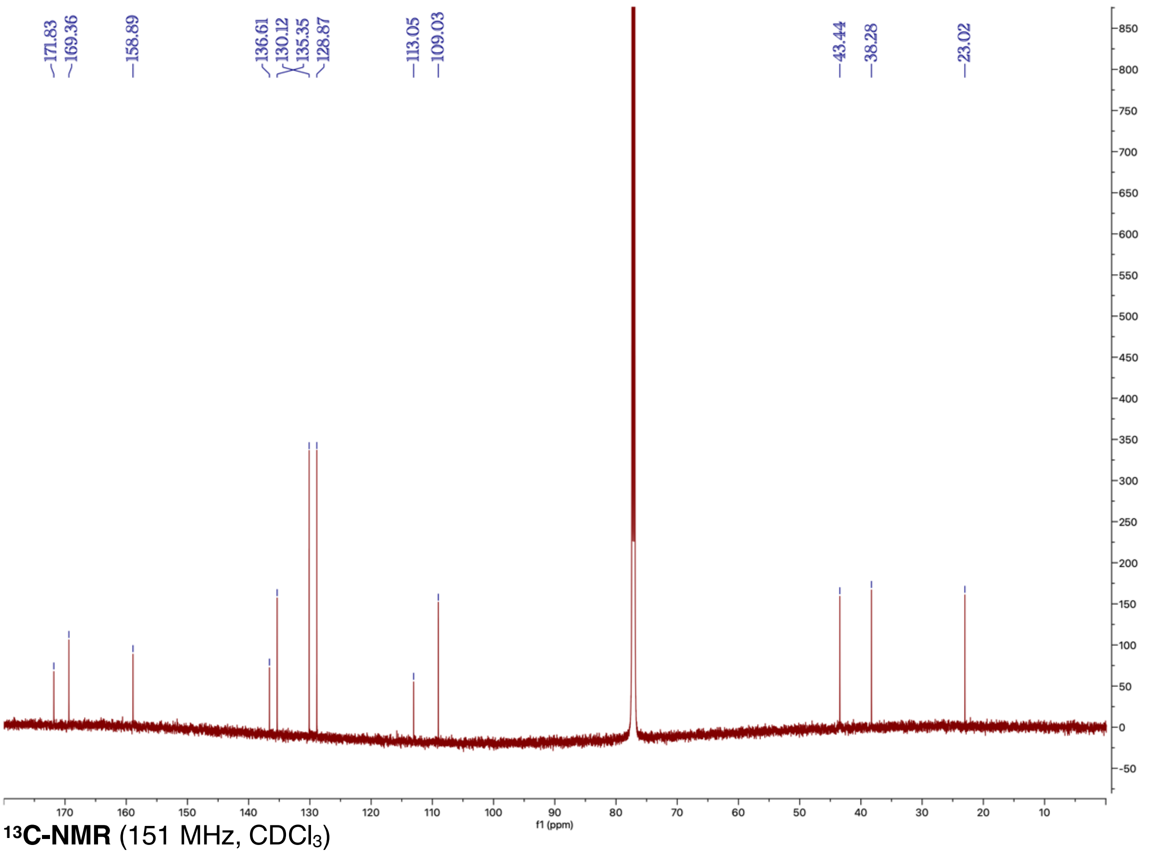

^{13}**C-NMR** (151 MHz, $CDCl_3$)

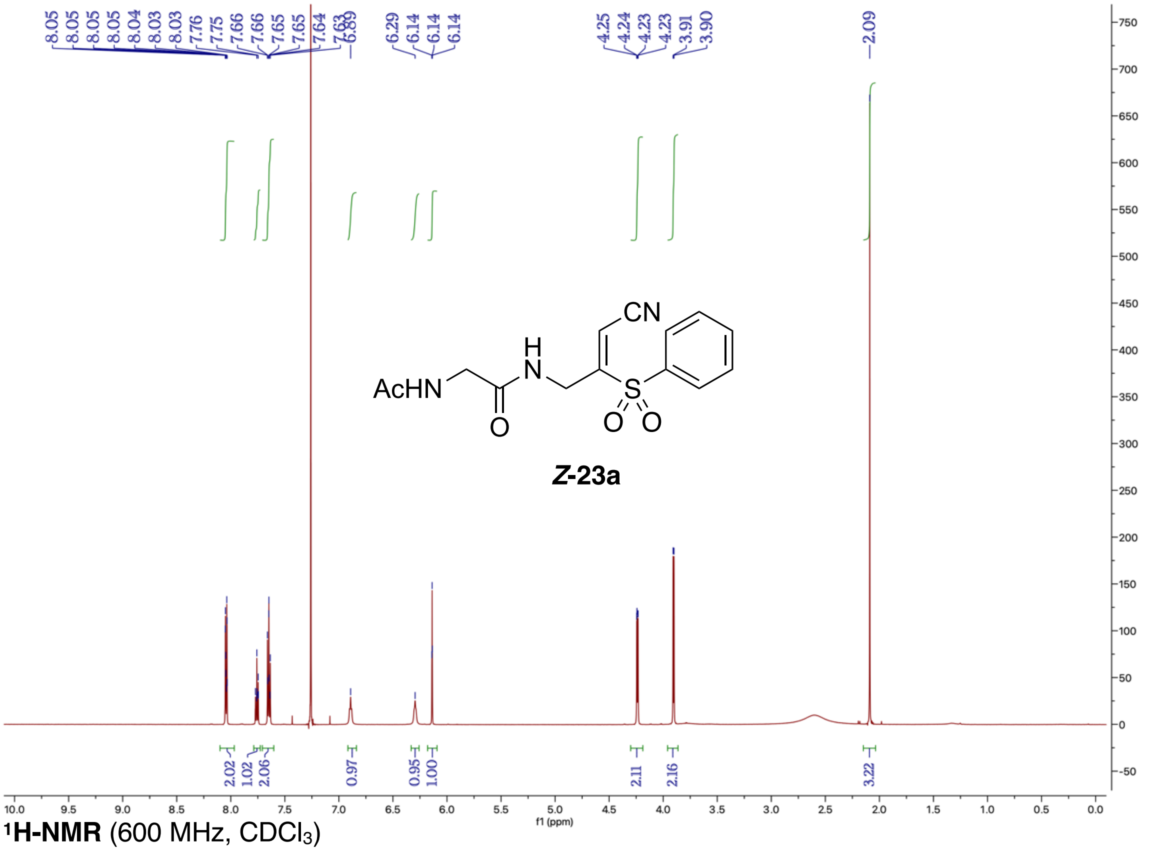

^{1}H-NMR (600 MHz, $CDCl_3$)

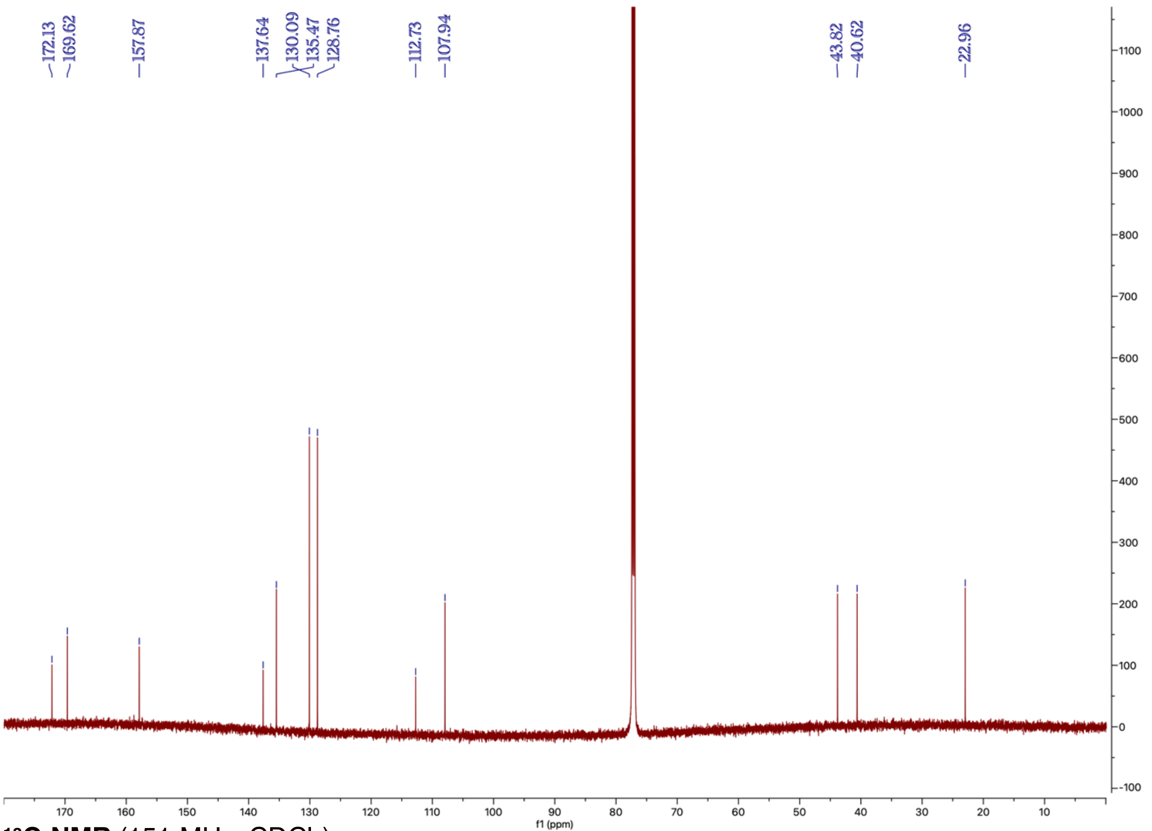

^{13}C-NMR (151 MHz, $CDCl_3$)

M. MASS SPECTRA (FULL SCANS FOR SUPPLEMENTARY FIGURES S7–S11)

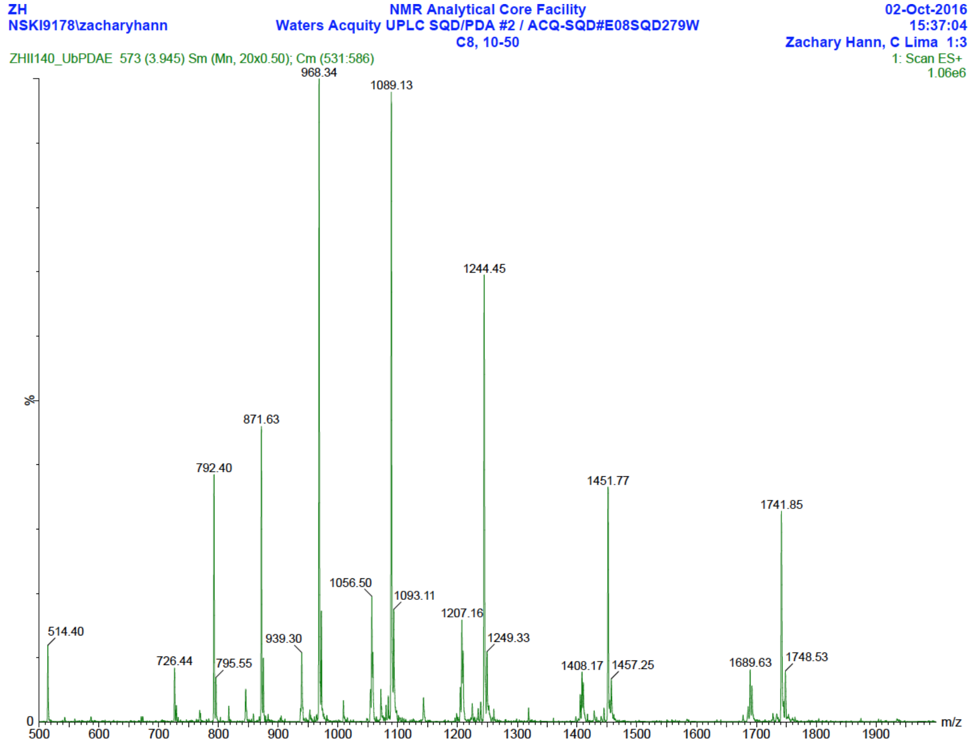

Full Scan Mass Spectra for Supplementary Figure S7 (top panel 1).

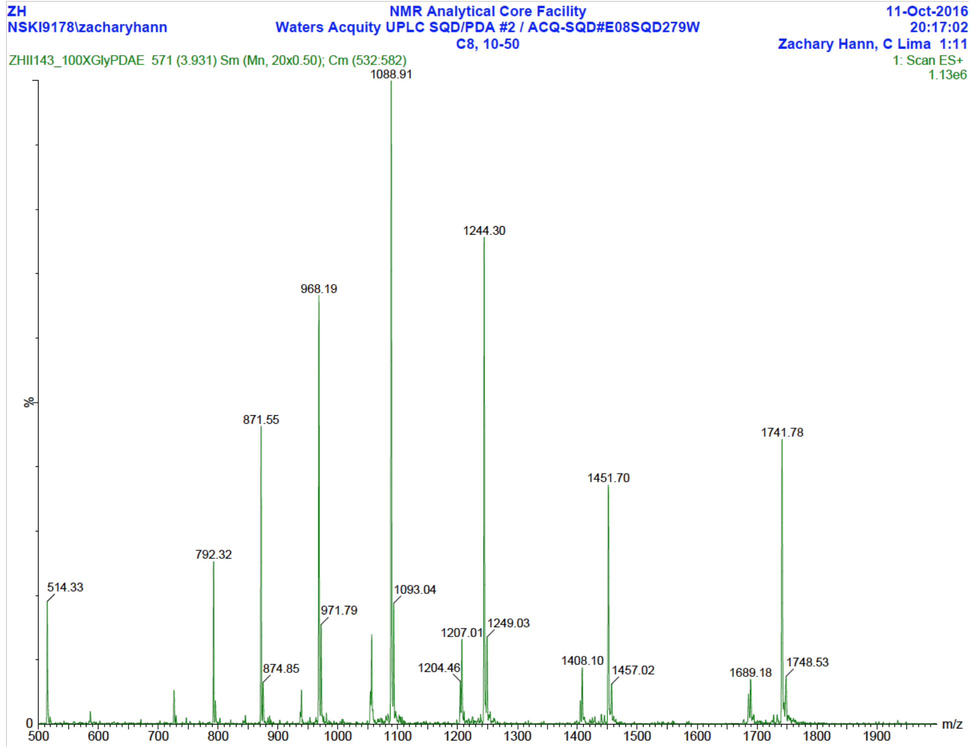

Full Scan Mass Spectra for Supplementary Figure S7 (top panel 2).

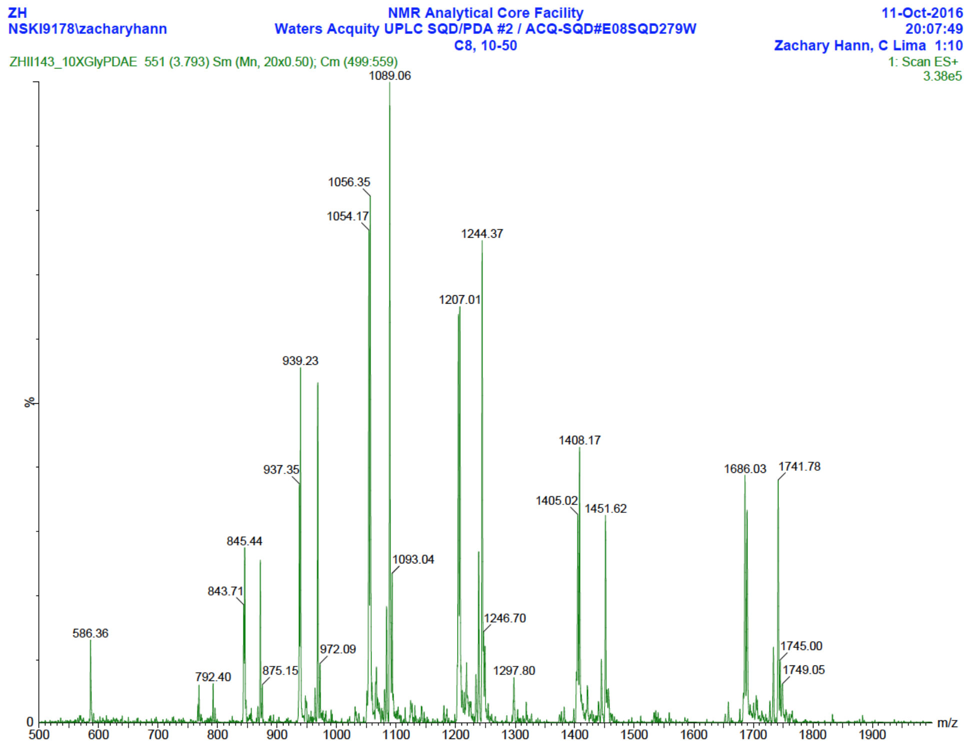

Full Scan Mass Spectra for Supplementary Figure S7 (top panel 3).

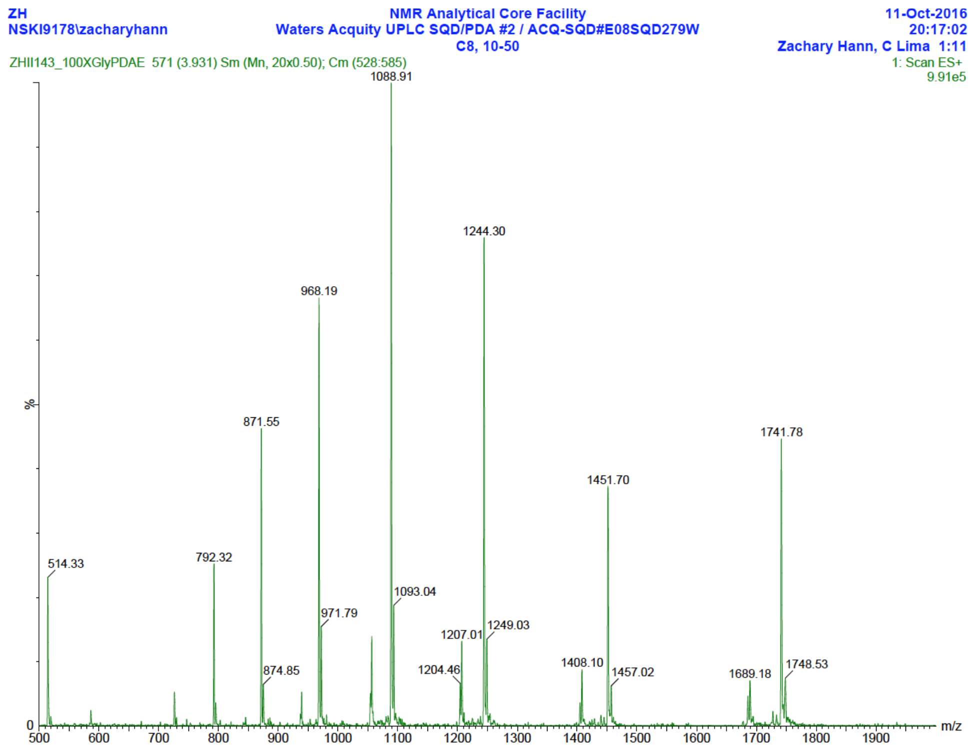

Full Scan Mass Spectra for Supplementary Figure S8 (top panel 1).

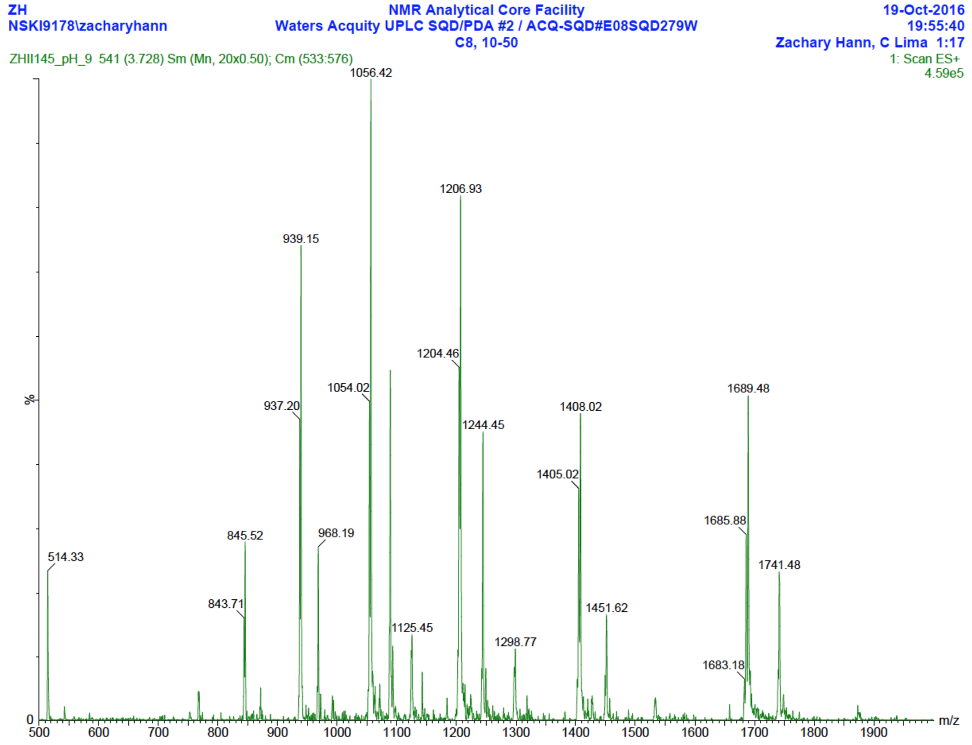

Full Scan Mass Spectra for Supplementary Figure S8 (top panel 2).

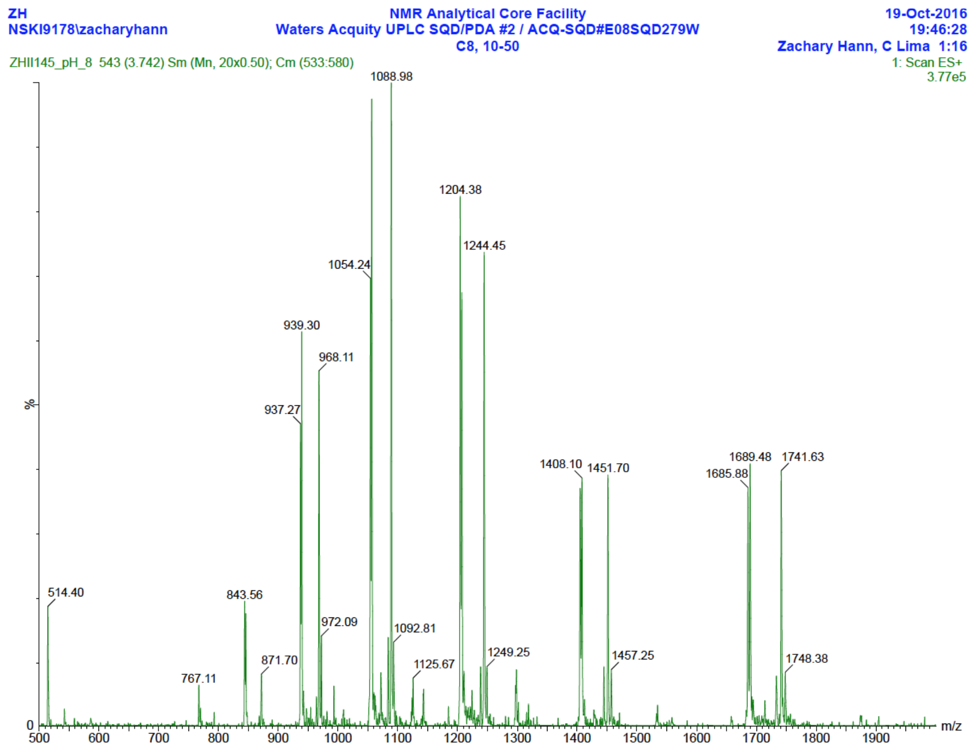

Full Scan Mass Spectra for Supplementary Figure S8 (top panel 3).

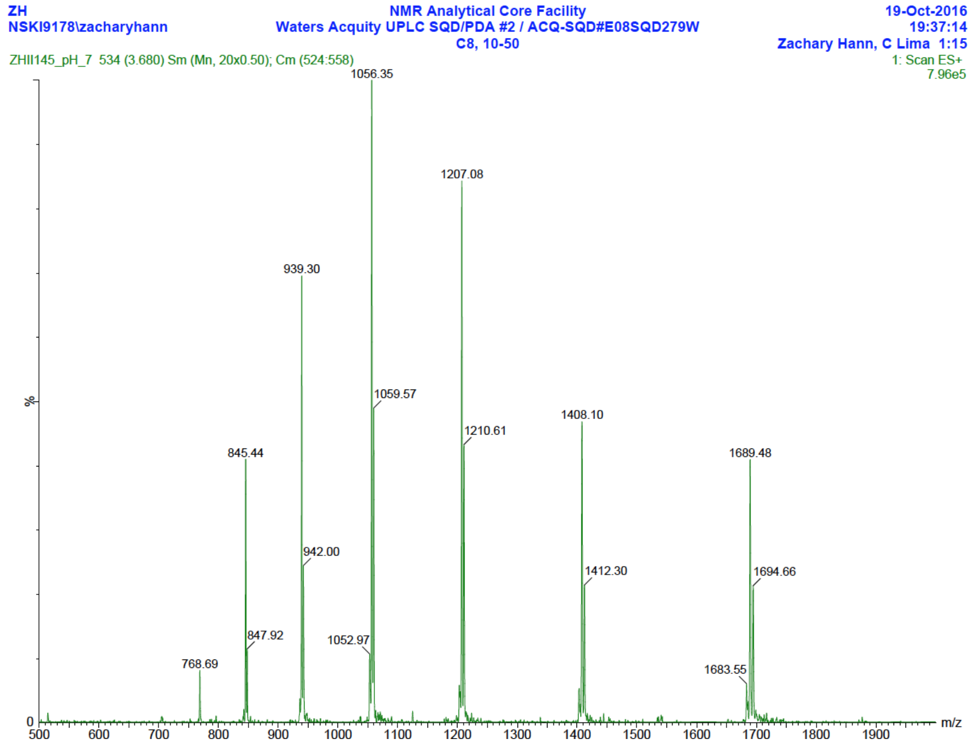

Full Scan Mass Spectra for Supplementary Figure S8 (top panel 4).

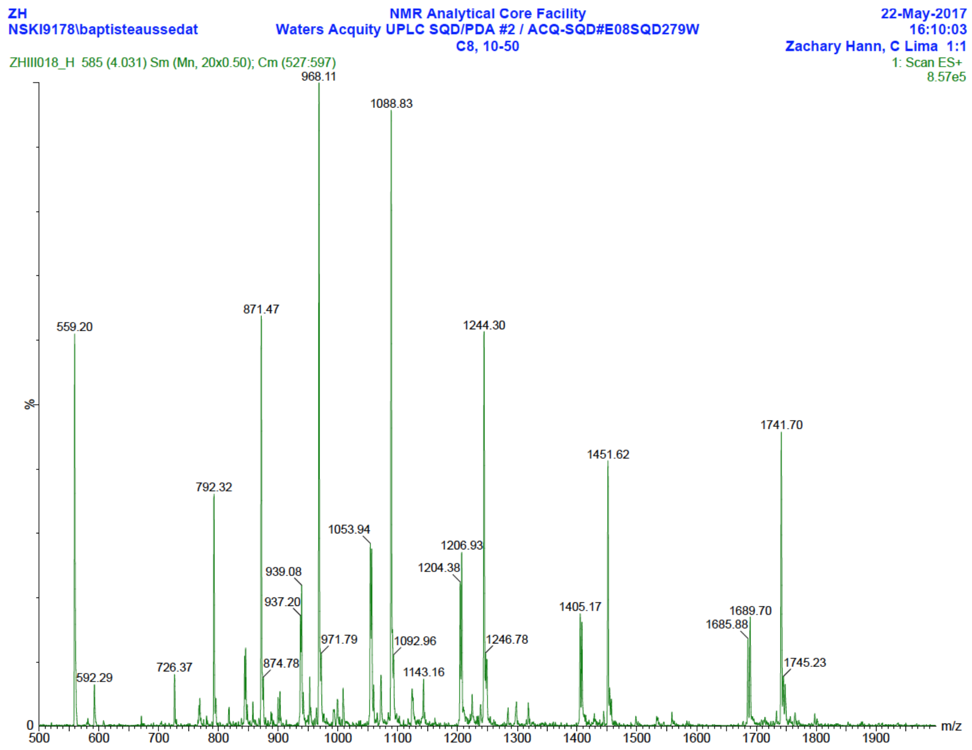

Full Scan Mass Spectra for Supplementary Figure S9 (top panel 1).

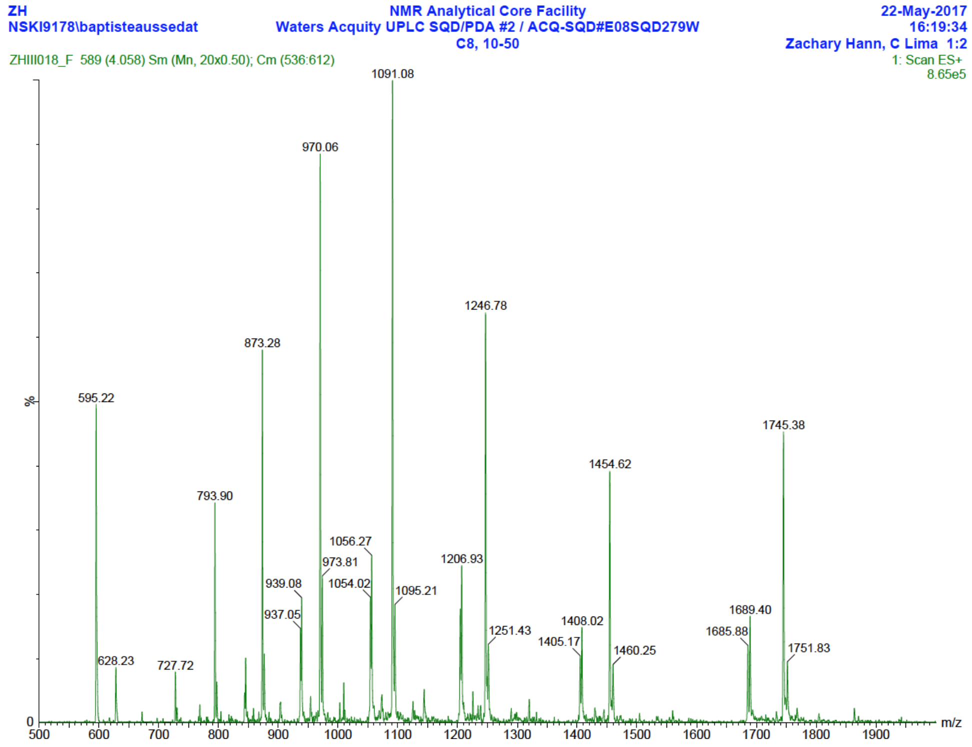

Full Scan Mass Spectra for Supplementary Figure S9 (top panel 2).

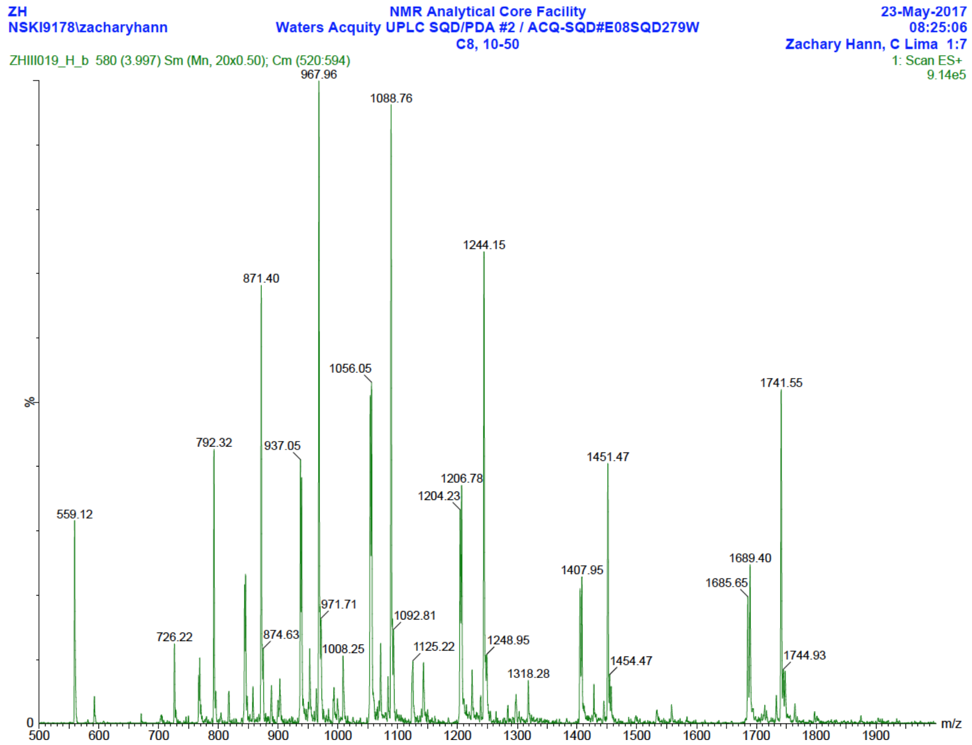

Full Scan Mass Spectra for Supplementary Figure S10 (top panel 1).

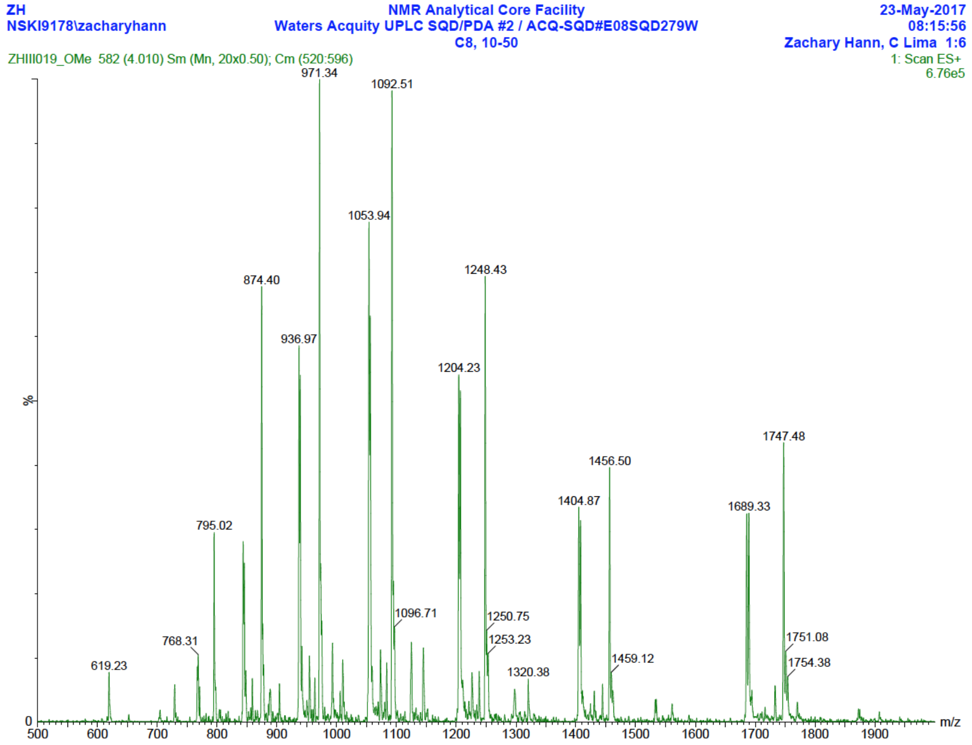

Full Scan Mass Spectra for Supplementary Figure S10 (top panel 2).

N. SDS-PAGE gels (source images for Figures 2b, 4b–d, S12, S13)

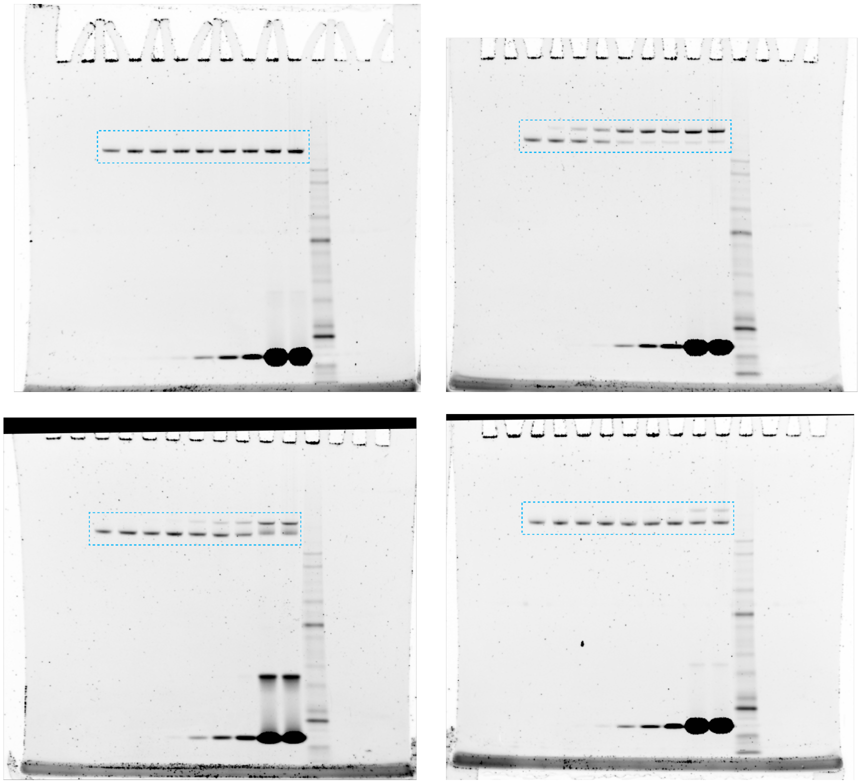

Source images for Figure 2b (1 h panels). Blue boxes indicate portions shown in main figure.

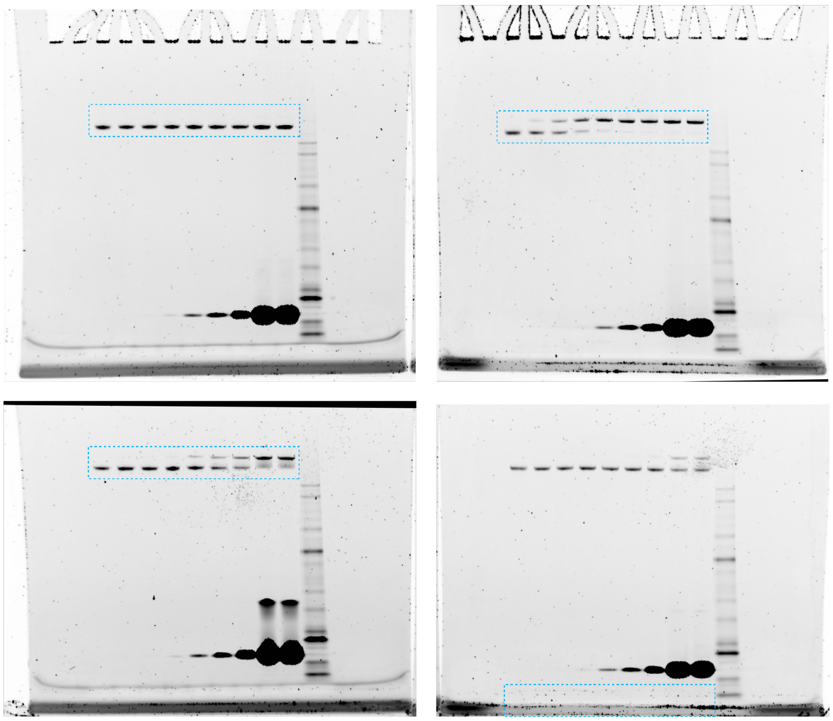

Source images for Figure 2b (15 h panels). Blue boxes indicate portions shown in main figure.

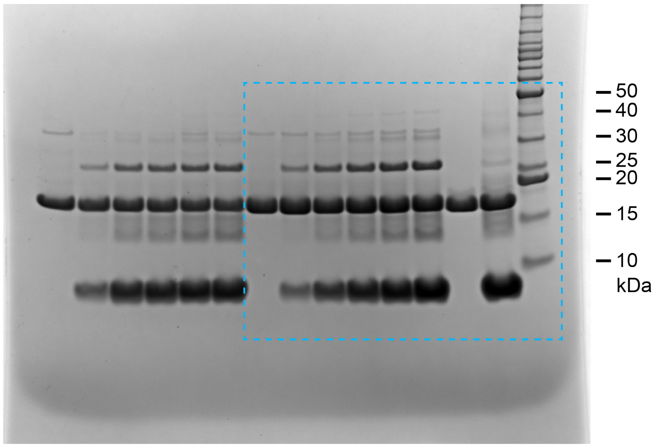

Source image for Figure 4b. Blue box indicates portion shown in main figure.

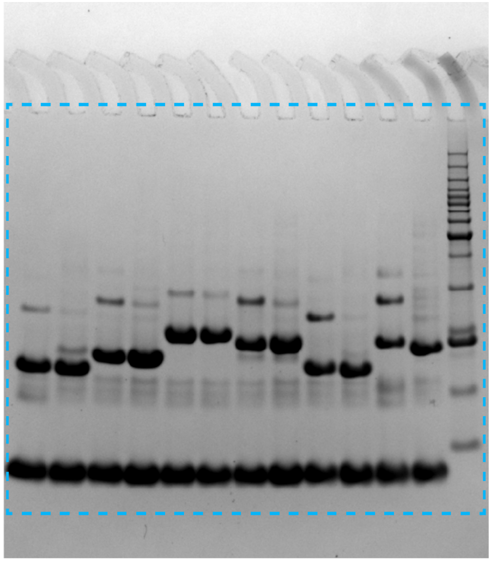

Source image for Figure 4c. Blue box indicates portion shown in main figure.

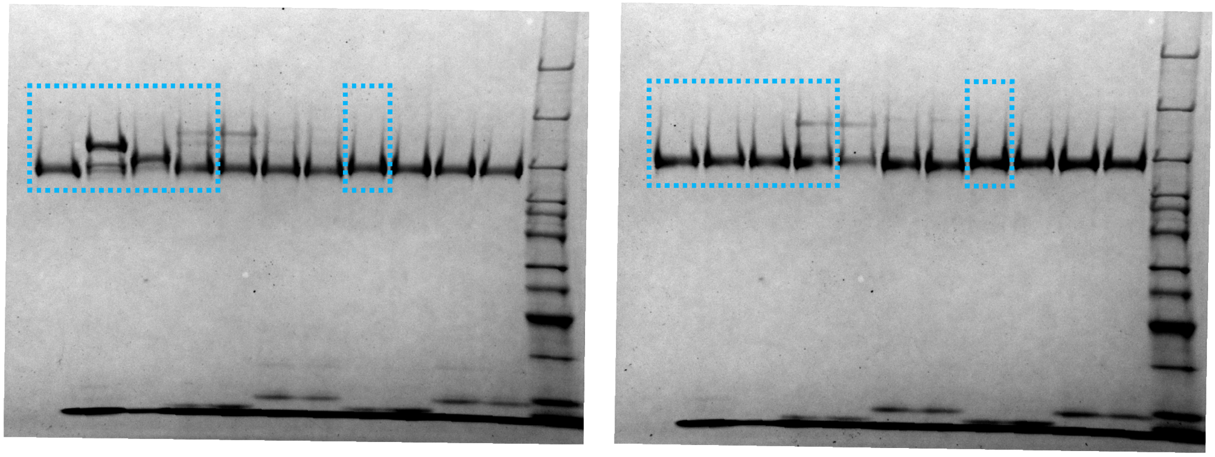

Source images for Figure 4d. Blue boxes indicate portions shown in main figure.

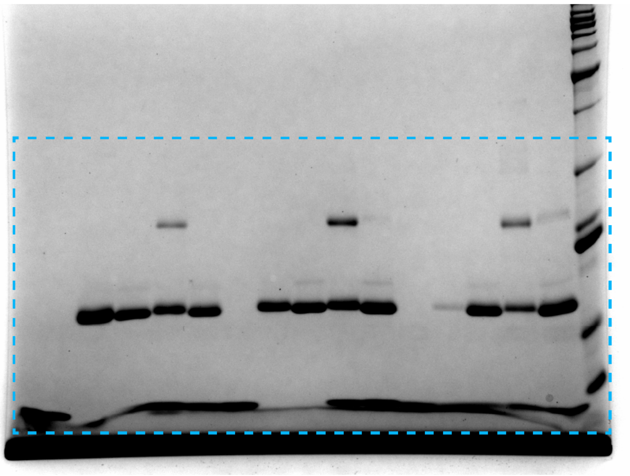

Source image for Supplementary Figure S12. Blue box indicates portion shown in main figure.

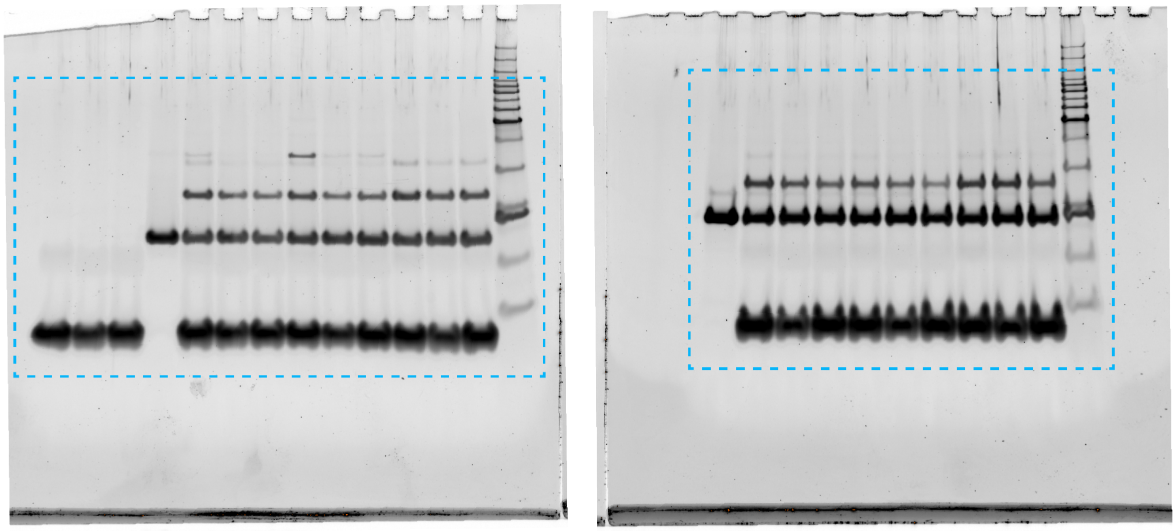

Source images for Supplementary Figure S13. Blue boxes indicate portions shown in main figure.
